# Competing effects of wind and buoyancy forcing on ocean oxygen trends in recent decades

**DOI:** 10.1038/s41467-024-53557-y

**Published:** 2024-10-26

**Authors:** Helene A. L. Hollitzer, Lavinia Patara, Jens Terhaar, Andreas Oschlies

**Affiliations:** 1 GEOMAR Helmholtz Centre for Ocean Research Kiel, 24148 Kiel, Germany; 2 Climate and Environmental Physics, Physics Institute, University of Bern, 3012 Bern, Switzerland; 3 Oeschger Centre for Climate Change Research, University of Bern, 3012 Bern, Switzerland; 4 Kiel University, 24118 Kiel, Germany

**Keywords:** Physical oceanography, Marine chemistry, Marine biology, Climate and Earth system modelling, Element cycles

## Abstract

Ocean deoxygenation is becoming a major stressor for marine ecosystems due to anthropogenic climate change. Two major pathways through which climate change affects ocean oxygen are changes in wind fields and changes in air-sea heat and freshwater fluxes. Here, we use a global ocean biogeochemistry model run under historical atmospheric forcing to show that wind stress is the dominant driver of year-to-year oxygen variability in most ocean regions. Only in areas of water mass formation do air-sea heat and freshwater fluxes dominate year-to-year oxygen dynamics. The deoxygenation since the late 1960s has been driven mainly by changes in air-sea heat and freshwater fluxes. Part of this deoxygenation has been mitigated by wind-driven increases in ventilation and interior oxygen supply, mainly in the Southern Ocean. The predicted slowdown in wind stress intensification, combined with continued ocean warming, may therefore greatly accelerate ocean deoxygenation in the coming decades. The fact that the model used here, along with many state-of-the-art forced ocean models, underestimates recent ocean deoxygenation indicates the need to use forcing fields that better represent pre-industrial conditions during their spin-up.

## Abstract (web summary)

Ocean deoxygenation since the late 1960s has been partially mitigated by wind-driven oxygenation, particularly in the Southern Ocean. The predicted slowdown in wind intensification may therefore accelerate ocean deoxygenation in the coming decades.

## Introduction

Oxygen (O_2_) is critical for sustaining marine life and regulates important elemental cycles in the ocean, such as those of nitrogen, phosphorus, and iron^1^. Observations indicate that the global ocean dissolved O_2_ inventory, currently estimated at 227.4 ± 1.1 Pmol^2^, has decreased by >1%^3^ and possibly >2%^2^ between 1960 and 2010, mainly in response to anthropogenic climate change. This deoxygenation is projected to continue in the coming decades, even under the low-emission, high-mitigation Shared Socioeconomic Pathways 1–2.6^4,5^ (SSP1–2.6) or a complete cessation of carbon dioxide emissions^6^. However, while there is a clear downward trend in the global oceanic O_2_ inventory, regional O_2_ responses to climate change vary widely across ocean basins and depths, implying spatial and temporal variability in the underlying drivers^2,7^.

Ocean oxygen distribution and changes are the result of a complex interplay of driving forces that either enhance or deplete O_2_. At the sea surface, ocean O_2_ is in direct contact with the atmosphere through air-sea gas exchange. Thus, waters within the mixed layer are, to first order, in equilibrium with the O_2_ partial pressure of the atmosphere, with the equilibrium oxygen concentration depending on the solubility of O_2_ in seawater and, therefore, primarily on the sea surface temperature (SST). Beneath the mixed layer, there are no significant sources of oxygen, and O_2_ can only be supplied by ventilation, defined as the physical processes by which (O_2_-rich) surface waters are transferred from the surface mixed layer into the ocean interior. In the interior ocean, these water masses remain isolated from the atmosphere over long timescales set by the interior transport patterns^8^, and their O_2_ content is consumed by the respiration of organic matter.

The supply of oxygen to the ocean interior is not homogeneous across the global ocean, but is largely concentrated in specific locations of water mass formation^8,9^. Ventilation is the result of a suite of interacting processes^10^, with the main atmospheric drivers being wind stress (i.e., the shear stress exerted on the ocean surface by wind) and air-sea heat and freshwater fluxes. Wind stress is a major driver of large-scale ocean circulation, and its divergence and convergence patterns force the gyre transports and the meridional overturning circulation^11,12^. Air-sea heat and freshwater fluxes regulate the transformation of surface water masses and deep ocean mixing^13^. Prominent regions of ocean interior oxygenation include the subpolar North Atlantic, where strong surface buoyancy loss triggers open-ocean convection^14^, the coastal regions around Antarctica, where Antarctic Bottom Water is formed^15,16^, and the regions of mode and intermediate water formation at mid-latitudes^17,18^, especially in the Southern Ocean. These intensely ventilated regions can all be traced as oxygen maxima throughout the ocean interior^19^. As opposed to these well-ventilated oxygen maximum zones, poorly ventilated sites often result in oxygen minimum zones (OMZs), mostly located in the eastern part of the tropical oceans^20,21^.

Anthropogenic climate change, manifested amongst other things by ocean warming^22^ and changing wind fields^23,24^, has had far-reaching effects on ocean oxygen concentrations in recent decades. Ocean warming directly reduces oxygen solubility and accounts for about 15% of the global oxygen loss^2,25^. Apart from the direct effect on solubility, anthropogenic warming also intensifies near-surface stratification^26^, which is suggested to reduce ocean oxygenation by impeding the transport of O_2_-rich surface waters into the permanent thermocline and by limiting the resurfacing of O_2_-poor deeper waters, thereby reducing the intensity of O_2_ uptake at the atmosphere-ocean interface^27^. On the other hand, stronger stratification may also mitigate deoxygenation by reducing the upward transport of nutrients, thereby limiting biological production in the euphotic zone and the subsequent export and oxygen-consuming respiration of organic matter^4,28^. Changing wind fields may also have the potential to both increase and decrease ocean oxygen supply. For example, the continued strengthening of the Southern Ocean westerlies^23,29^ has contributed to increased formation rates of oxygen-rich intermediate water masses, which according to models may enhance global ocean oxygen supply^30^. Conversely, the strengthening of the Pacific trade winds since the 1990s^24,31^ has led to intensified wind-driven nutrient upwelling, greater biological activity, and consequently increased O_2_ consumption below the surface ocean^32^.

Despite recent advances in understanding the drivers of regional O_2_ changes, our present understanding of the spatial distribution of O_2_ changes and their causes remains limited, partly due to the superposition of a number of forcings and mechanisms that complicate clear attribution^33^. While climate change is known to affect ocean oxygen by modifying wind fields and air-sea heat and freshwater fluxes^27^, the quantitative contribution of these factors to ocean deoxygenation remains poorly constrained in observations. Observations do not allow to separate the effect of the different drivers of oxygen changes, i.e., changing wind fields and changing heat and freshwater fluxes. Also, oxygen measurements in the deep ocean are sparse and gridded observation-based data products typically provide O_2_ estimates only to depths of 1000 m^34^ or 2000 m^35^, leaving oxygen dynamics below this depth poorly constrained.

One way to quantify the contribution of each driver and to deconstruct the superimposed mechanisms is the use of global ocean biogeochemistry models (GOBMs) run under historical atmospheric forcing. GOBMs have been shown to underestimate observationally estimated deoxygenation in the upper 700 m in the past two decades, attributed to their typical spin-up procedure that uses present-day atmospheric forcing also for pre-industrial conditions. This spin-up procedure leads to an overly warm ocean at the beginning of the hindcast period, resulting in an underestimation of the transient ocean heat uptake^36^ and associated deoxygenation^37^. While this spin-up bias does not exist in fully coupled earth system models (ESMs), ESMs do not have the same phasing of the internal climate variability as observed, so the simulated interannual variability of oxygen changes also differs from reality. Also, ESMs are typically run at low resolution^38,39^, which limits their ability to capture the effects of mesoscale eddies. Thus, despite the spin-up bias, GOBMs remain the only tool that allows isolating the role of different components of atmospheric forcing in driving historical regional and decadal changes in oxygen, albeit their underestimation of deoxygenation trends over the last two decades.

In this study, we investigate the interannual to decadal variability of O_2_ over the period 1967–2018 using a GOBM at 0.25^∘^ horizontal resolution, run under historical atmospheric forcing. To isolate the effects of changing wind stress and air-sea heat and freshwater fluxes on ocean O_2_, we perform a set of hindcast and sensitivity experiments run under different atmospheric forcing (see Methods). We identify the key drivers that have contributed to a sustained negative trend in the global oceanic oxygen inventory over recent decades. This trend is primarily due to changes in air-sea heat and freshwater fluxes, which affect gas solubility, ocean circulation, and ocean interior residence times. Although an increase in wind stress has led to increased ocean ventilation, particularly in the Southern Ocean, this only partially offsets the overall deoxygenation trend. Given the importance of recent changes in wind stress in mitigating global deoxygenation, a slowdown in wind stress intensification, as predicted by Earth system models^23,40^, may accelerate ocean deoxygenation in the coming decades.

## Results

### Trends in the simulated global ocean oxygen inventory

**Fig. 1:**
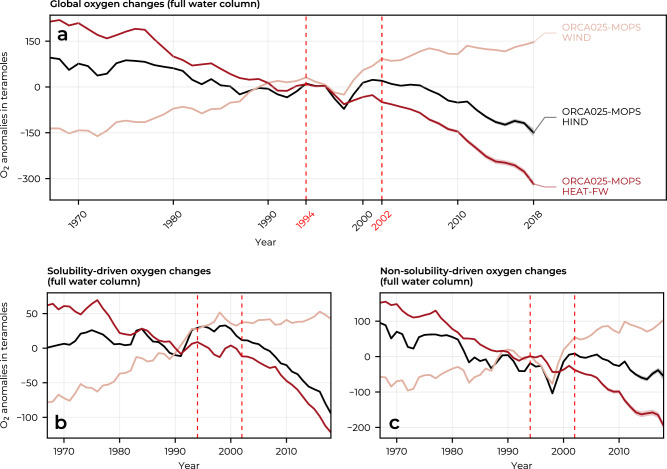
Change in the global oceanic oxygen inventory in the hindcast and sensitivity experiments. Globally integrated time series (1967–2018) of **a** oxygen inventory anomalies, **b** ${{{\rm{O}}}}_{2}^{{{\rm{sat}}}}$ anomalies, and **c** the residual between **a** and **b**, i.e., non-solubility-driven changes, for the HIND (black), WIND (rose), and HEAT-FW (red) experiments (see Methods). The lines represent averages over the two sets of experiments (see Methods), with the shading indicating the range between the minimum and maximum estimates. No uncertainty has been computed for the ${{{\rm{O}}}}_{2}^{{{\rm{sat}}}}$ anomalies in **b**, as each pair of experiments share the same physics and do not differ in their ${{{\rm{O}}}}_{2}^{{{\rm{sat}}}}$ estimates. All data are mean-centred using the 1967–2018 long-term mean. Red dashed vertical lines delineate the three periods of the oxygen inventory trajectory described in the results section. Note the different y-axis scales in the different panels. Source data are provided as a Source Data file.

The simulated trajectory of the global oceanic oxygen inventory from 1967 to 2018 can be separated into three major periods (Fig. 1a, Supplementary Table 1). In the first period, from 1967 to 1994, the global O_2_ inventory gradually decreases at a rate of –46.4 (±5.0, standard error of the estimated linear slope) Tmol O_2_ per decade (hereafter Tmol dec^−1^). In the second period, from 1994 to 2002, the simulated trend in the O_2_ inventory departs from the long-term declining trend, first falling anomalously fast until 1998 and then recovering rapidly until 2002. From 2002 to the end of the simulation period (2018), the simulated global oceanic oxygen inventory decreases continuously at an accelerated rate of −116.8 ± 6.6 Tmol dec^−1^.

**Fig. 2:**
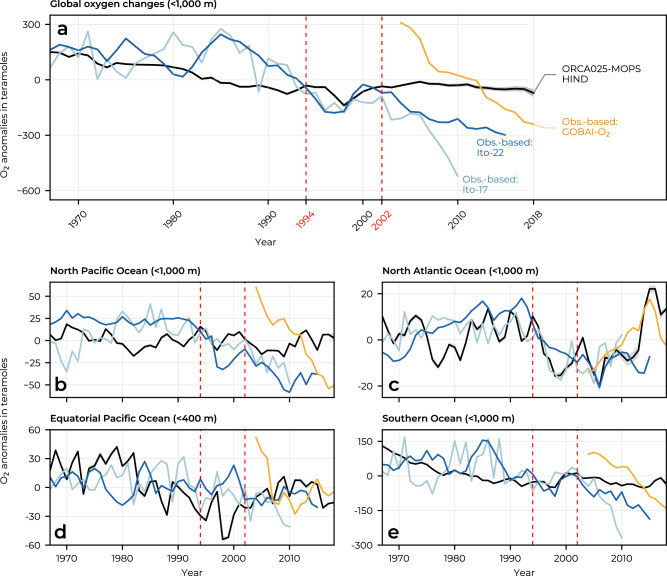
Change in global and regional oceanic oxygen inventories in the hindcast experiment and comparable observation-based data. Time series (1967–2018) of **a** globally integrated upper 1000 m oceanic oxygen inventory anomalies simulated by ORCA025-MOPS HIND (black) and observation-based data from Ito-17^35^ (light blue), Ito-22^43^ (dark blue), and GOBAI-O_2_^44^ (yellow). For the model data, averages over the two sets of experiments (see Methods) are shown, with the shading indicating the range between the minimum and maximum estimate. No uncertainty estimate is reported for Ito-17^35^. GOBAI-O_2_ includes uncertainty estimates from measurement, gridding and algorithmic sources^44^. Ito-22 includes uncertainty estimates from mapping errors, unresolved small-scale and high-frequency variability^43^. These uncertainties are not comparable to the uncertainties of our model estimates and are therefore not shown. For each dataset, the data are mean-centred using the long-term mean calculated over the time span plotted. **b**–**e** show the same as **a**, but for four sub-regions (Supplementary Fig. 9). Red dashed vertical lines delineate the three periods of the oxygen inventory trajectory described in the results section. Note the different y-axis scales in the different panels. Source data are provided as a Source Data file.

Ocean deoxygenation varies considerably with depth. Between 1967 and 1994, virtually all deoxygenation occurred in the upper 1000 m, as can be seen by comparing Fig. 1 (total water column O_2_ changes) and Fig. 2 (top 1000 m O_2_ changes), and as shown in Supplementary Table 2. Likewise, the transient low in the global oceanic O_2_ inventory between 1994 and 2002 was also almost entirely confined to the upper 1000 m and thus left deeper layers unaffected (Supplementary Table 2). From 2002 to 2018, both depth layers, above and below 1000 m, experienced a decrease in oxygen levels, with 34% of this loss occurring in the upper 1000 m and 66% below 1000 m (Supplementary Table 2). According to these model results, deeper waters are only recently affected by deoxygenation and have been left unchanged far longer than the first 1000 m.

**Fig. 3:**
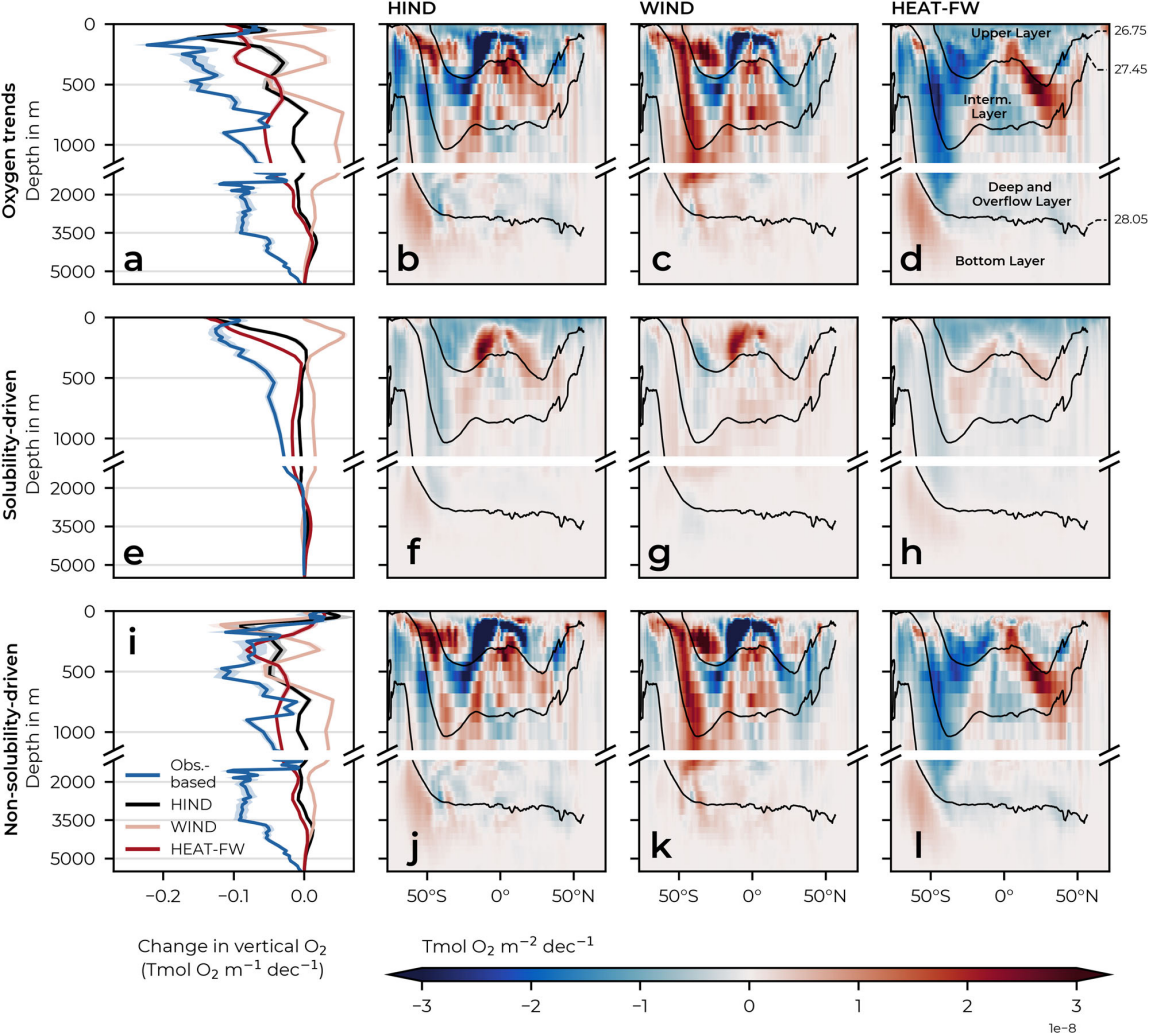
Trends in ocean oxygen by depth and latitude. Panels **a**–**d** show linear trends (1967–2018) in oxygen content as a function of **a** depth and **b**–**d** as a function of depth and latitude. The panels below show the same as **a**–**d**, but decompose the oxygen change into its **e**–**h** solubility-driven (${{{\rm{O}}}}_{2}^{{{\rm{sat}}}}$) and **i**–**l** non-solubility-driven components. Trends are shown for HIND, the sensitivity experiments WIND and HEAT-FW (see Methods), and for the observation-based data products Ito-22^43^ for oxygen (1967–2015) and EN4.2.2^104^ for ${{{\rm{O}}}}_{2}^{{{\rm{sat}}}}$ (and their combination ${{{\rm{O}}}}_{2}^{{{\rm{total}}}}$ - ${{{\rm{O}}}}_{2}^{{{\rm{sat}}}}$ to compute the non-solubility-driven component). The shading in the line graphs indicates the standard error of the estimated linear least-squares regression slopes and black contours show the the neutral density surfaces *γ*^*n*^ = 26.75, 27.45, and 28.05 kg m^−3^, i.e., the approximate boundaries of the upper, intermediate, deep, and bottom layers. Source data are provided as a Source Data file.

We further decompose O_2_ changes into changes in O_2_ saturation, ${{{\rm{O}}}}_{2}^{{{\rm{sat}}}}$ (i.e., the thermodynamic component driven by changes in solubility), and the remaining oxygen changes, which are not driven by changes in solubility but by changes in circulation and biological activities (see Methods). Globally, solubility and non-solubility-driven changes tend to co-evolve to first order (Fig. 1), but their relative contributions to the total oxygen change differ in time (Fig. 1) and with depth (Fig. 3). From the late 1960s to the late 1990s, non-solubility effects accounted for virtually all of the deoxygenation (Fig. 1b, c). Afterward, the model suggests that non-solubility-driven and solubility-driven changes each contribute about half of the global oxygen depletion. This shift from predominantly non-solubility-driven deoxygenation to a roughly equal contribution from solubility is consistent with observation-based estimates (Supplementary Table 3). This change coincides with an acceleration of the rise in global Ocean Heat Content (OHC), although the model underestimates this increase, as discussed in the following section. The highest rate of solubility-driven deoxygenation occurs within the upper 300 m of the water column (Fig. 3e), with non-solubility-driven changes becoming dominant below the thermocline in the model (Fig. 3i).

The simulated non-solubility-driven part of the global O_2_ decrease is mainly due to changes in ocean stratification and ventilation rather than changes in remineralisation. In our simulations, remineralisation rates gradually decrease throughout the deoxygenation period 1967–2018 (Supplementary Fig. 1), indicating a slight reduction in respiratory oxygen consumption rather than an increase. The overriding importance of ventilation changes in the non-solubility-driven part of global deoxygenation is consistent with projections from an Earth system model (1990s–2090s, RCP8.5), which show that a decrease in subduction contributes to a deoxygenation trend that outweighs the mitigating effect of reduced respiration^41^.

### Comparison to observation-based estimates of oxygen

We compare the simulated global O_2_ inventory with observation-based gridded data products of ocean oxygen, which use statistical and machine learning methods to interpolate for data gaps in space and time. Specifically, we use data from Ito et al.^34^ (Ito-17), T. Ito, 2022^42^ (Ito-22), and GOBAI-O_2_^35,43^. The time course of the simulated global O_2_ inventory of the surface ocean down to 1000 m depth is similar to the observation-based estimates Ito-17 and Ito-22 from 1967 to 1994 (Fig. 2a, Supplementary Table 4). In the mid-1990s, both simulated and observation-based estimates show a dip in the global oxygen inventory, albeit with a slightly different temporal phasing (Fig. 2a). Substantial discrepancies are found after 2002 between the observation-based and simulated estimates (Fig. 2a, Supplementary Table 4). After 2002, the observation-based data products Ito-17, Ito-22, and GOBAI-O_2_ show a strong decrease in oceanic O_2_, which far exceeds the deoxygenation rate observed between 1967 and 1994. In contrast, the model simulates a nearly stagnant global oceanic oxygen inventory for the upper 1000 m after 2002.

**Fig. 4:**
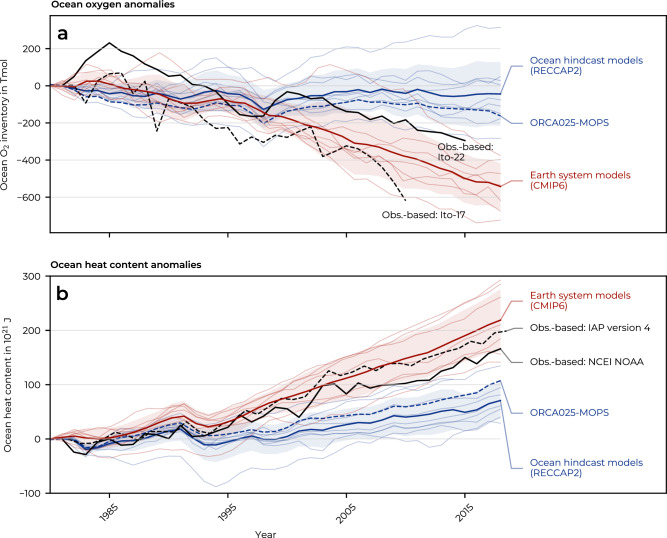
Change in ocean oxygen and ocean heat content estimated by observation-based and model-derived datasets. Annual time series of **a** ocean oxygen and **b** ocean heat content (OHC) anomalies in the upper 700 m for different observation-based and model-derived datasets. The model-based estimates are split into estimates from ocean hindcast models (datasets associated with the RECCAP2 effort^107^), shown in blue, and Earth system models from CMIP6^108–115^, shown in red. For each set, the multi-model mean is shown as a bold solid line, and the associated standard deviation is shaded. Thin coloured lines show individual model estimates, and the dashed blue line shows the ORCA025-MOPS model estimate (HIND). Observation-based data are shown in black, with ocean oxygen data sourced from Ito et al.^35^ and T. Ito^43^, and OHC data from the National Oceanic and Atmospheric Administration (NOAA), with data updated from Levitus et al.^106^, and IAPv4^105^. Anomalies are calculated with respect to 1980. Source data are provided as a Source Data file.

The misrepresentation of present-day deoxygenation in the model has been attributed to the spin-up procedure used by most GOBMs^44^, including this study. The reduced sensitivity to global warming (Supplementary Fig. 2) leads to an underestimation of OHC increase^36^ and of the closely-linked deoxygenation^37^. This issue is prevalent not only in the model analysed here, but also in the GOBMs participating in RECCAP2^45^ (Fig. 4), which contribute largely to the Global Ocean Carbon Budget^46^. The discrepancy between modelled and observationally estimated deoxygenation after 2002 due to the biased spin-up is evident in the misrepresentation of both solubility-driven (down to 2000 m depth) and non-solubility-driven (down to 400 m depth) components (Supplementary Fig. 3).

In both the Schmidtko et al.^2^ and EN4 datasets, solubility-driven deoxygenation spreads deeper into the water column, reaching ~2000 m depth. This deep solubility-driven deoxygenation is not captured by the model and accounts for the majority of the bias. Non-solubility-driven processes, primarily ventilation, contribute less to the bias, consistent with the simulated mixed layer depth being overestimated rather than underestimated (Supplementary Fig. 4). During the well-simulated period 1967–1994, both solubility-driven and non-solubility-driven components of the O_2_ trend agree well with observation-based estimates (Supplementary Fig. 5), with a notable exception: Ito-22 shows substantial non-solubility-driven deoxygenation also in the 1700–5000 m depth range (Fig. 3i) throughout the simulation period (1967–2018), mainly in the Southern Ocean, as shown in Supplementary Fig. 6. This trend is not captured by the model, but the observation-based estimates should be treated with caution due to the paucity of deep-ocean oxygen measurements, especially in the Southern Ocean.

While the model underestimates deoxygenation in the upper 1000 m of the water column between 2002 and 2018, Fig. 2a shows that also the observation-based data products suffer from substantial uncertainties. These data products differ greatly in their estimated deoxygenation rates, with GOBAI-O_2_ estimating a rate almost three times higher than that of Ito-22 (Supplementary Table 4) and highlights the need to consider the inherent uncertainties also in observation-based products when evaluating model performance^47^.

### Global drivers

The global deoxygenation since the 1970s has been driven by changes in air-sea heat and freshwater fluxes (hereafter buoyancy fluxes), while changes in wind stress have mitigated the overall oxygen loss (Fig. 1). Since 1967, air-sea buoyancy fluxes have led to an average decrease in the global oxygen inventory of −94 ± 3 Tmol dec^−1^ (Fig. 1a, Supplementary Table 1). Of this oxygen loss, 32% is attributed to reduced solubility, and the remaining 68% is attributed to accumulation of non-solubility-driven oxygen losses (Supplementary Table 1). This global ocean oxygen depletion has been partly counteracted by a steady increase in wind stress (Supplementary Fig. 7), which has increased the global ocean O_2_ inventory by about 64 ± 2 Tmol dec^−1^ since 1967 (Fig. 1a), through its effect on both oxygen solubility (Fig. 1b) and non-solubility-driven oxygen changes (Fig. 1c).

The acceleration of deoxygenation over the last two decades has been caused by a combination of smaller wind-driven O_2_ increases and acceleration of O_2_ losses due to changes in air-sea heat and freshwater fluxes (Fig. 1a). Oxygen depletion imposed by changes in buoyancy fluxes nearly doubled between 2002 and 2018 compared to the period between 1967 and 1994 (Supplementary Table 1), coinciding with an increase in ocean heat uptake around 2000^48^. As the model underestimates the ocean heat uptake over the last two decades, we expect this buoyancy-driven deoxygenation to be even higher in reality (Fig. 4). At the same time, although there is still some increase in ocean oxygen due to wind stress-driven processes, this increase is only about 40% the magnitude of the wind stress-driven oxygen increase estimated between 1967 and 1994 (Supplementary Table 1).

### Regional estimates

Trends and variability in ocean oxygen vary across ocean regimes, reflecting the complexity and regional differences in the underlying drivers. The highest levels of O_2_ short-term variability are found, for example, in strongly dynamic regions of water mass formation, strong ocean currents, and ocean-sea ice interaction (Supplementary Fig. 8). Instead, there is little variability in the centre of the subtropical gyres and in the Weddell and Ross gyres. These regional patterns of oxygen variance are effectively reproduced by the model when compared to observation-based estimates (Supplementary Fig. 8).

The spatial extent of regions of high O_2_ variability in observation-based products often appears to be larger than modelled, especially in the Southern Ocean. This relatively large spatial extent results from the scarcity of observations that have to be extrapolated over large spatial scales each year. As a consequence, the O_2_ values in each cell of the observational product are not always derived from observations at the same location, and the high variability regions of the observation-based product are stretched. The high-resolution model, on the other hand, correctly captures only the interannual variability at specific locations and does not need to rely on spatial extrapolation.

**Fig. 5:**
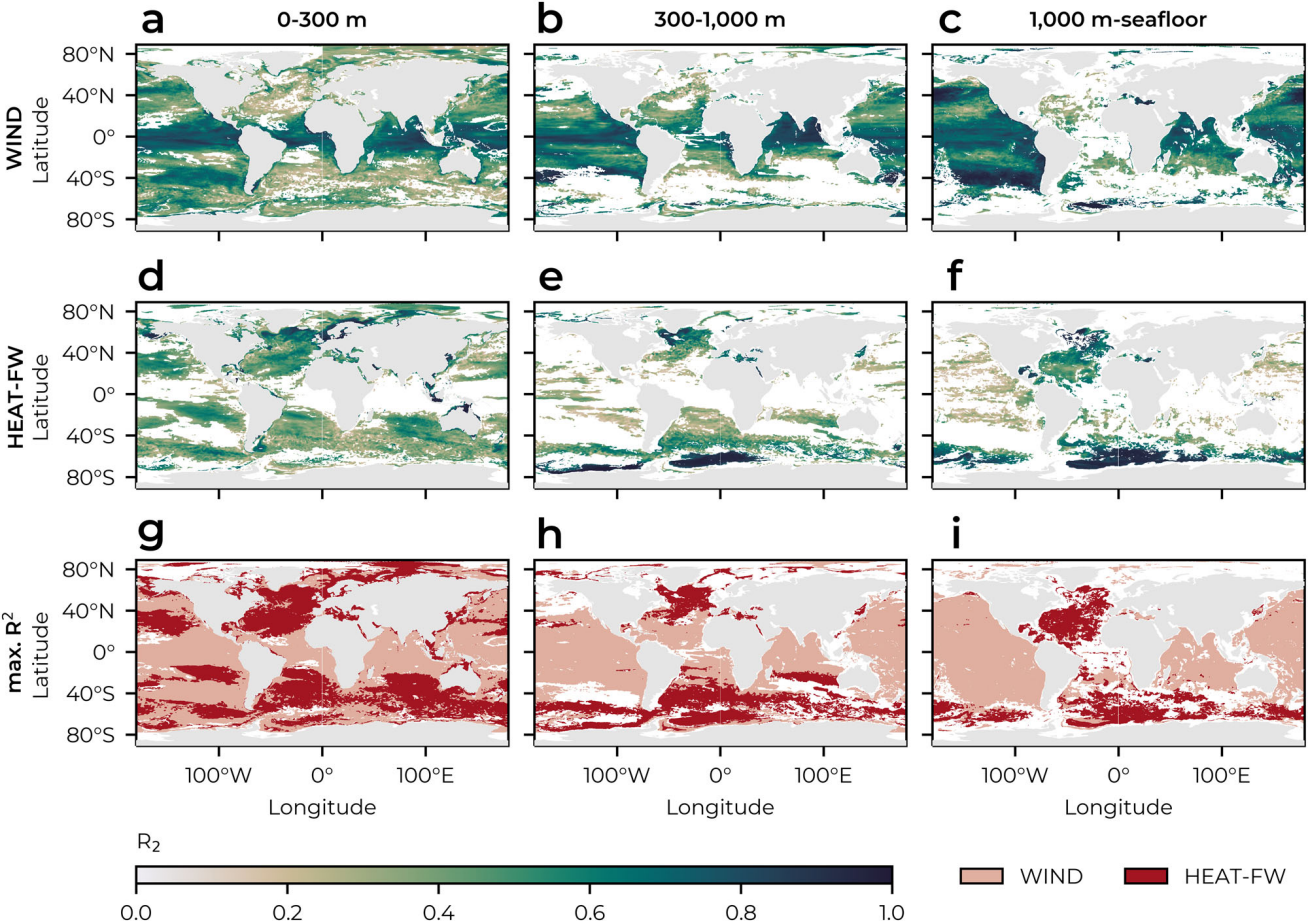
Correlation analysis between the oxygen inventory time series in the hindcast and those in the sensitivity experiments. **a**–**f** shows the coefficient of determination (*R*^2^) between the oxygen inventory time series (1967–2018) in HIND and those in **a**–**c** WIND and **d**–**f** HEAT-FW (see Methods) for the three depth horizons 0–300 m, 300–1000 m, and below 1000 m (columns). Regions, where the Pearson correlation coefficient is non-significant (*p* > 0.05), are shown in white, denoting non-significance. Based on the *R*^2^ values, **g**–**i** show, for each gridpoint, whether the oxygen in HIND aligns more closely with that in WIND (rose) or HEAT-FW (red). Regions where HIND is not significantly correlated with either sensitivity experiment are shown in white. This analysis captures the relationship between the experiments on timescales from interannual to multi-decadal. Source data are provided as a Source Data file.

We find that while changes in air-sea buoyancy fluxes are the main driver of the global long-term deoxygenation trend, wind stress is the dominant driver of the year-to-year variability of O_2_ in most ocean regions (Fig. 5). Only in areas of water mass formation, such as upper ocean mode waters in the subtropical gyres (Fig. 5g), mode and intermediate waters in the mid-latitude Southern Ocean (Fig. 5g, h), and deep and bottom waters in the North Atlantic and close to Antarctica (Fig. 5i), air-sea buoyancy fluxes are the main drivers of oxygen dynamics and explain a larger part of the interannual variability. Although the air-sea buoyancy flux affected areas are small, they largely affect the interior ocean O_2_, as the newly-formed water masses penetrate deep below the surface ocean.

We further analyse in more detail the oxygen inventories for four distinct regions (Fig. 2b–e, Supplementary Fig. 9): the North Pacific Ocean, the North Atlantic Ocean, the equatorial Pacific Ocean, and the Southern Ocean. By comparing these regional trends with observation-based estimates, we find that while global deoxygenation is generally underestimated, regional trends can be captured with greater accuracy (Fig. 2). In the North Atlantic, for example, the O_2_ estimates from Ito-17 and GOBAI-O_2_ are very similar to the O_2_ time series simulated by the model after 1990 and 2010, respectively (Fig. 2c).

Owing to its large volume, Northern Hemisphere oxygen trends in the upper 1000 m (Fig. 3b) are dominated by changes in the North Pacific Ocean (Supplementary Fig. 6n). This ocean region is known for its large regional and temporal variations in dissolved oxygen, which are strongly related to the Pacific Decadal Oscillation^49^. Substantial shifts in oxygen injection into the thermocline waters have been shown to result from variations in surface outcrops of different mode water masses, located primarily in the western North Pacific and affecting water masses lighter than about *γ*^*n*^ = 26.6 kg m^−3^ (generally found at depths  <500 m)^50^. For waters lighter than *γ*^*n*^ = 26.6 kg m^−3^, our model shows a pronounced wind stress-driven oxygen decline (Supplementary Fig. 6o), consistent with an observation-based analysis^51^. In addition, the buoyancy-driven simulated oxygen increase in heavier water masses (Supplementary Fig. 6p) at densities between *γ*^*n*^ = 26.6 kg m^−3^ and about *γ*^*n*^ = 27.6 kg m^−3^ (generally found at depths  <1000 m) is consistent with the observation-based analysis of Mecking and Drushka^52^. Although the simulated patterns of change are consistent with previous observational studies, the simulated deoxygenation, when integrated over the entire North Pacific, is too low compared to the observation-based estimates analysed here (Fig. 2b).

In the North Atlantic basin, we find that the model captures well the decadal O_2_ variability (Fig. 2c). Here, the O_2_ variability is largely driven by air-sea heat fluxes and associated subpolar convective activity and MOC strength (Supplementary Fig. 10) modulated by the North Atlantic Oscillation^53,54^. Similar to observation-based estimates, oxygen inventories are highest in the years of high convection in the 1990s^55^ and in 2014–2015^54^. In depth space, we find that deoxygenation in the upper ocean is superimposed on an oxygenation trend in the intermediate and deep layers (Supplementary Fig. 6f), driven mainly by changes in buoyancy fluxes. This pattern is consistent with observation-based data^53^ showing a deoxygenation trend between 1960–2009 in the mode and upper intermediate waters, driven by increased stratification, and an oxygenation trend in the lower intermediate waters and Labrador Seawater, driven by strong subpolar convection in the 1990s.

The equatorial regions show an overall deoxygenation that is strongest in the 100–400 m range, consistent with the observed expansion of the tropical OMZs^21^. Our findings indicate that this deoxygenation is primarily driven by wind stress-induced reductions in ventilation (Fig. 3k), predominantly originating in the Pacific Ocean (Supplementary Fig. 6). This is consistent with the concomitant weakening and shoaling of the OMZ-ventilating subtropical cells (STCs) in the model (Supplementary Fig. 11), and shown in observations^56^. The shoaling of the STCs creates a pattern of deoxygenation in the 100–400 m range and oxygenation below, possibly due to the influx of more oxygenated waters. A similar pattern occurs in the Indian Ocean, and past studies have found that subsurface oxygenation has been driven by a reduction in waters supplied by the Indonesian Throughflow in favour of high-oxygen waters supplied by the Southern Indian Ocean Gyre^57,58^.

The importance of wind stress in tropical regions, as shown in Fig. 5 and Supplementary Fig. 6b–d, is in line with previous studies showing that variations in the strength of tropical trade winds strongly influence oxygen concentrations locally^32,59^. The large dip in O_2_ in 1998, and to a lesser extent in 1983 and 2016, is consistent with the notion that during El Niño^60^, shallower and less intense upwelling may reduce the upward transport of low-O_2_ waters, thereby reducing the O_2_ influx from the atmosphere^59^. However, these dips in oxygen levels in the equatorial Pacific are not fully visible in the observational datasets analysed here (Fig. 2d).

The O_2_ temporal evolution in the Southern Ocean (Fig. 2e) closely reflects that found at a global scale (Fig. 2a), showing a similar trend in the model and observation-based datasets until the early 2000s (albeit with a different phasing of the year-to-year variability) and a strong divergence thereafter. The mismatch in interannual variability in the early period may be due to the fact that the precipitation dataset before 1979 does not contain interannual variability^61^, and water mass transformation in the Southern Ocean is known to be driven more by freshwater than heat fluxes^62,63^. The fact that the underestimation of global deoxygenation since 2002 is mainly due to the Southern Ocean is not surprising, since spin-up effects persist there for decades to centuries as large amounts of old water upwell in the Southern Ocean divergence zone. Similarly, the largest biases in the ocean carbon sink due to atmospheric CO_2_ in the spin-up are also in the Southern Ocean^64^.

At the same time, Southern Ocean observation-based estimates of deoxygenation must be interpreted with caution due to the paucity of oxygen measurements in this under-sampled region (see e.g. Supplementary Fig. 1 in ref. ^35^ and Fig. 1 in ref. ^43^). Large disagreements also exist between the trends estimated by observation-based datasets in the Southern Ocean (Fig. 2e). The differences between Ito-22 and Ito-17 can be attributed to three main factors^42^: data sources, interpolation methods, and mapping parameters. Ito-22 relies solely on WOD bottle O_2_ data, while Ito-17 combines both bottle and CTD data, resulting in different spatial coverage and data density. The restricted availability of source data, particularly in Ito-22, results in substantial data gaps that must be interpolated^42^, introducing uncertainty, especially in the under-sampled Southern Ocean. In comparison, GOBAI-O_2_ uses a broader data set, including both GLODAP bottle O_2_ data and Argo float measurements, and employs machine learning techniques, including random forest regression and feed-forward neural networks, for data set development^43^. The large discrepancies in the Southern Ocean, therefore, likely reflect the sensitivity of these observation-based datasets to different data sources and the challenges of interpolation in poorly sampled regions such as the Southern Ocean.

The paucity of oxygen measurements in the Southern Ocean also casts doubt on the accuracy of the representation of interannual oxygen changes in observation-based datasets, given the high agreement between model and observation-based estimates in a very well-sampled region such as the North Atlantic^34,42^ (Fig. 2b). The accuracy of observation-based estimates of interior O_2_ changes in the Southern Ocean is likely low, given that even observation-based estimates of the air-sea CO_2_ flux, which are based on a larger number of surface ocean observations than estimates of interior O_2_ change, have also been shown to overestimate variability in the Southern Ocean^65^ as well as trends^66^. Due to the shortcomings of model and observation-based estimates of O_2_ inventory changes in the Southern Ocean, we cannot conclude with certainty whether model or observation-based estimates are closer to the true O_2_ inventory changes in this ocean basin.

The wind stress-driven oxygenation trend found at a global scale (Fig. 1a) can be predominantly traced back to non-solubility-driven oxygenation at intermediate depths in the Southern Ocean (Fig. 3k). A steady strengthening of the Southern Hemisphere westerly winds in past decades^29,67^ is thought to have strengthened the upper cell of the MOC^68^ and to have increased the ventilation of Subantarctic Mode Water and Antarctic Intermediate Water^69–71^. In our model results, the upper cell of the Southern Ocean MOC also shows a long-term strengthening (Supplementary Fig. 11), much of which is attributed to the wind stress strengthening (Supplementary Fig. 7). Wind stress-driven oxygenation at intermediate levels has partly been counteracted by concomitant changes in air-sea heat and freshwater fluxes that have reduced ventilation^71^, likely leading to ventilation-driven deoxygenation at intermediate depths of the Southern Ocean (Fig. 3l).

## Discussion

In this study, we performed a set of sensitivity experiments with a high-resolution GOBM to decompose the contributions of air-sea heat freshwater fluxes and wind stress to global and regional patterns of oxygen changes. This series of experiments allowed us to identify the underlying drivers of a persistent negative trend in the global oceanic oxygen inventory since the late 1960s. We found that the overall oceanic deoxygenation was driven by changes in air-sea heat and freshwater fluxes, and was only partially counteracted by a concomitant wind stress-driven increase in ocean ventilation, mainly originating in the Southern Ocean.

While the model is generally consistent with observation-based oxygen trends until the early 2000s, the severity of observationally estimated deoxygenation from 2002 to 2018 is underestimated by a factor of about 10 compared to both Ito-17 and GOBAI-O_2_, and by a factor of about 4 compared to Ito-22. We found that biases in ${{{\rm{O}}}}_{2}^{{{\rm{sat}}}}$ drive most of the deoxygenation bias in in the model (Supplementary Fig. 3), while non-solubility-driven processes, chiefly ventilation, contribute less to the deoxygenation bias. A large fraction of the missing global deoxygenation in the model after 2002 originates in the Southern Ocean (Fig. 2). However, determining the precise magnitude of this underestimation is complicated by substantial uncertainties also in the observation-based data products (Fig. 2). This situation highlights the need to increase both the temporal and spatial coverage of oxygen measurements, ultimately strengthening the robustness of the essential observation-based oxygen data products. This is particularly important in the Southern Ocean, where oxygen measurements are scarce, but the region is key to global oxygen dynamics.

The misrepresentation of present-day deoxygenation, a common deficiency in state-of-the-art ocean models^37^, has been attributed to the spin-up procedure used by most GOBMs (including this study), which results in reduced sensitivity to global warming, underestimated OHC rise^36^ and associated deoxygenation^37^. This bias is particularly strong after 2000, which may be due to a stronger rise in the OHC after the 1990s, which is not accurately reproduced by the model (Fig. 4), especially at intermediate depths where the model is already overly warm (Supplementary Fig. 2). This discrepancy may result in less accurate simulations of oxygen trends in upwelling regions (Supplementary Fig. 12), because intermediate and deep waters have absorbed too much heat already during the spin-up phase and can therefore absorb less heat in the last 1958–2018 cycle. Conversely, and as also reported by Takano et al.^37^, coupled Earth System Models (ESMs) from CMIP6, which often share the ocean components with GOBMs, simulate upper 700 m deoxygenation and ocean warming trends that are consistent with observation-based estimates within uncertainties (Fig. 4). Future modelling studies should adjustthe forcing fields used during spin-up to be more representative of pre-industrial conditions, as successfully implemented by Huguenin et al.^36^.

While the deoxygenation trend since the early 2000s is underestimated in the model, the trends before 2002, which are less affected by the recent OHC rise, are better captured. Also, the trends in the WIND experiment, which by construction does not capture the direct effects of global warming (but only its indirect effects manifested through wind field changes), are less biased than the trends in HEAT-FW. At a regional scale, the model performs well in capturing the regional patterns of maximum O_2_ variability and the decadal O_2_ variability driven by North Atlantic convection. This ability can be explained by the fact that regionally most of the O_2_ variability is driven by its non-solubility component (Supplementary Fig. 13), so that the known ${{\rm{O}}}_{2}^{{{\rm{sat}}}}$ bias does not exert a significant influence. Ventilation and convection are intimately linked to ocean circulation, which in turn is driven by wind stress and air-sea heat and freshwater fluxes. Since these processes depend critically on the ability of the model to accurately simulate the large- and small-scale circulation features^72^, we argue that high-resolution GOBMs, such as the one used here, are better suited to capture the regional patterns of O_2_ changes than coarse-resolution ESMs.

A key region for global oxygen trends is the Southern Ocean, where the formation of mode and intermediate waters is a key conduit for oxygen, nutrients, and anthropogenic carbon and heat into the interior ocean^73,74^. In this region, westerly winds have strengthened in recent decades due to increasing greenhouse gas emissions and stratospheric ozone depletion^29^, which have been shown to have significant and distinct effects on biogeochemistry^75^. Our results suggest that the wind strengthening has counteracted the deoxygenation caused by widespread surface warming and mid-depth freshening^76,77^. The poleward shift and implicit movement of the sinking branch of intermediate water masses into denser water may have further accelerated intermediate water mass formation^68^, while increasing oxygen solubility in subducted waters. By modulating the strength of the Southern Ocean upper circulation cell^68^ and setting the formation rates and regions of oxygen-rich intermediate water masses^70,71^, the Southern Hemisphere westerly winds are critical for global oxygen supply^30^.

Recent studies suggest that the current intensification of westerlies in the Southern Hemisphere may slow or cease altogether in the coming century under low to moderate emissions scenarios that align with or approach the temperature targets of the Paris Agreement^23,40^. In these scenarios, the slowdown in wind stress intensification and the concomitant vanishing of wind-driven oxygen enrichment is attributed to the recovery of stratospheric ozone by mid-century^78^. The reduction in ventilation-driven oxygen supply under these low emissions scenarios is likely to coincide with additional losses caused by ocean warming, even if CO_2_ emissions are stopped^79–81^. The ocean will continue to warm to equilibrate with the warmer atmosphere and thus continue to lose oxygen^6,82,83^. Our study raises the question of whether the stabilisation or reversal of the past intensification of wind stress, together with continued ocean warming, will accelerate oxygen loss in the future, particularly in the Southern Hemisphere intermediate waters and in regions fed by these water masses, such as the equatorial OMZs^84^.

Our analysis demonstrates the complex interplay between the counteracting forces of wind stress and air-sea heat and freshwater fluxes, emphasising their importance in determining changes in oceanic oxygen. Given the centrality of atmospheric drivers in influencing oceanic oxygen trends, we stress the importance of accurately representing wind stress and air-sea heat and freshwater fluxes in reanalysis data, including pre-industrial conditions used during spin-up to assess past deoxygenation with GOBMs. In addition, a robust projection of wind stress and air-sea heat and freshwater fluxes in coupled ESMs is essential for a robust prediction of future changes in O_2_ and OHC. This analysis identifies the drivers of deoxygenation and their regional patterns. It contributes to a much-needed improved mechanistic understanding of O_2_ changes, critical to better predict and anticipate future global and regional O_2_ inventory changes and their potential consequences for marine ecosystems.

## Methods

### ORCA025-MOPS model

We used a global configuration of the ocean-sea ice model NEMO-LIM2^85^ with a horizontal resolution of 0.25° (ORCA025) and 46 unequally spaced vertical levels that increase with depth^86^. The model resolution is sufficient to capture much of the mesoscale eddy spectrum^87^, so no eddy parameterisation was used. The ocean-sea ice model was coupled to the marine biogeochemical model MOPS^88,89^, which simulates the lower trophic levels of the ecosystem and the associated nutrient cycling using nine tracers: phosphate (P), nitrate (N), O_2_ (O), dissolved inorganic carbon, alkalinity, and fixed-stoichiometry representations of phytoplankton, zooplankton, detritus, and dissolved organic matter. We find that the choice of biogeochemical parameters, and in particular the stoichiometric O_2_:P ratio, can influence the long-term trends and adjustment times of ocean oxygen^88,90^. To evaluate the uncertainty associated with the choice of the O_2_:P ratio, we employ two different model configurations with different values of the parameter (O_2_:P ratio of either 150 or 162).

ORCA025-MOPS was initialised by a spin-up performed with the 0.5° resolution model ORCA05-MOPS. ORCA05-MOPS was initialised with climatological temperature and salinity from Levitus98^91^, with World Ocean Atlas 2013 conditions^92,93^ for PO_4_, NO_3_, and O_2_, and with GLODAPv2 conditions^94^ for alkalinity and *C*_nat_ (pre-industrial carbon). The model was forced by the JRA55-do runoff dataset (version 1.1, at 0.25^∘^ horizontal resolution) and JRA55-do atmospheric forcing dataset (version 1.4, at 0.25^∘^ horizontal and 3-hourly temporal resolution) from 1958 to 2018^61^. ORCA05-MOPS was run under three cycles of JRA55-do atmospheric forcing, amounting to a total spin-up of 183 years. The end of the third cycle of ORCA05-MOPS provided the biogeochemical initial conditions for a spin-up with the 0.25^∘^ resolution model ORCA025-MOPS, run under one cycle of JRA55-do atmospheric forcing. For technical reasons, the physics had to be restarted from Levitus98. The end of the fourth cycle provided the initial conditions for the experiments analysed in this study.

Errors in the representation of biogeochemical processes in the model may bias the results, particularly in the low-oxygen zones along the eastern margins where biogeochemical processes are of high importance in altering the oxygen demand^95^. It has been suggested that the temperature dependence of remineralisation may impact O_2_ concentrations through shoaling of remineralisation profiles^96^. In MOPS, remineralisation is temperature-independent, so these effects are neglected. The distribution of phytoplankton in the surface layer (~6 m) in ORCA025-MOPS is generally in good agreement with observation-based estimates (Supplementary Fig. 14), crucial for accurately representing remineralisation. The strongest discrepancies are found in coastal regions, where modelled phytoplankton biomass is typically below that observed (Supplementary Fig. 14), possibly due to simplifications in the representation of terrestrial nutrient runoff, including the absence of additional and increasing nutrient inputs from anthropogenic sources^97^. In the pelagic ocean, modelled phytoplankton biomass is typically higher than observed (Supplementary Fig. 14), likely due to the neglect of iron limitation in the model^89^. While the calibrated parameters of ORCA025-MOPS may mitigate this deficiency somewhat^90^, this omission may still lead to an overestimation of primary production and concomitant remineralisation and oxygen loss below the sea surface, especially in the Southern Ocean and upwelling regions where iron is a limiting nutrient^98,99^. Although zooplankton is less homogeneously distributed than phytoplankton, the spatial differences from observation-based estimates are similar, with both being overestimated around the equator and in the Southern Ocean at 40^∘^S. The sparse data on zooplankton biomass, however, makes a robust assessment difficult (Supplementary Fig. 14).

### Simulations

For both model configurations (O_2_:P ratio of 150 or 162), four experiments were run. A hindcast (HIND) experiment, performed under interannual forcing of JRA55-do, simulates changes in the O_2_ inventory due to climate change and climate variability. However, HIND may also contain O_2_ changes introduced by a spurious model drift, as the model is not expected to be in equilibrium after the relatively short spin-up time compared to deep-ocean equilibration timescales. To quantify this model drift, we performed a repeated-year-forcing (RYF) experiment, which was integrated by repeating the JRA55-do forcing of a single year (1 May 1990 to 30 April 1991), most neutral in terms of the major climate modes^100^. The RYF experiment does not include changes in the O_2_ inventory due to climate change nor due to climate variability, so any ongoing changes are caused by the model drift. To correct for such model drift, the O_2_ inventories in RYF were removed (gridpoint-wise and for each year) from those in HIND, under the assumption that the drifts in HIND and RYF are the same. As RYF simulates neither climate change nor climate variability, the difference between the two experiments results in O_2_ inventory anomalies caused by climate change and variability only. This procedure is commonly used to differentiate between steady-state and non-steady-state components of ocean biogeochemical tracers^45,46^, including O_2_^101^. Depending on the selected value of the O_2_:P ratio, the drifts in the two RYF experiments are +0.23 and −0.13 Pmol O_2_ per decade (Supplementary Fig. 15a). The choice of the O_2_:P ratio does not, however, substantially influence the HIND minus RYF O_2_ anomalies. The O_2_ anomalies are nearly identical between the two experiment twins (Supplementary Fig. 15b), suggesting that the biogeochemical parameterisation does not substantially influence the decadal climate-driven O_2_ trends and variability that are the focus of this paper.

Following the strategy used in Patara et al.^71^, two complementary sensitivity experiments were performed to isolate the effects of changing air-sea heat and freshwater fluxes and wind stress on oxygen dynamics. In the air-sea heat and freshwater fluxes experiment (HEAT-FW), the interannual variability of the wind stress was suppressed, while the interannual variability of all variables needed to compute the air-sea fluxes of heat, freshwater, and oxygen was preserved. The opposite is true for the wind stress experiment (WIND), where only the interannual variability of wind stress was retained. The atmospheric variables used to compute the air-sea fluxes of heat, freshwater and oxygen are wind speed, air temperature, humidity, incoming solar radiation, outgoing longwave radiation, and precipitation. Similarly, RYF was subtracted from HEAT-FW and WIND to isolate the effects of interannual variability and climate change in air-sea heat and freshwater fluxes (for HEAT-FW) and in wind stress (for WIND) on oceanic O_2_. In the text, HIND, HEAT-FW, and WIND refer to the average of the drift-corrected results from each of the two model configurations. Note that in all four experiments, the years 1958 to 1967 were not included in the analyses performed due to the initial shock and associated recovery phase that the model undergoes when the forcing abruptly jumps from 2018 back to 1958^36,37,44^.

Changes in oceanic dissolved oxygen were further decomposed into two fractions for analysis of the underlying mechanisms: (1) solubility-driven changes and (2) non-solubility-driven changes (i.e., oxygen changes driven by changes in respiration or ventilation). O_2_ solubility in seawater was approximated by ${{{\rm{O}}}}_{2}^{{{\rm{sat}}}}$, calculated from potential temperature and salinity^102^, and represents the O_2_ concentration that a water mass has reached when in equilibrium with the O_2_ partial pressure of the overlying atmosphere. Non-solubility-driven oxygen changes were calculated by subtracting the solubility component from the total oxygen anomaly^34^, thereby isolating the fraction of oxygen changes that cannot be explained by solubility changes, and corresponds to the opposite of the commonly used Apparent Oxygen Utilisation (AOU) diagnostic.

### Observation-based datasets

We compare the simulated oxygen trends with observation-based estimates. Specifically, we use the observation-based data products GOBAI-O_2_^35,43^ (2004–2022), and oxygen concentration anomalies developed by Ito et al.^34,42^, referred to as Ito-17 (1950–2015) and Ito-22 (1965–2015), respectively. Due to the relatively low sampling density in Ito-17 before 1960 and after 2010^34^, only data from 1960–2010 were used. As Ito-17 and our simulations provide O_2_ anomalies, GOBAI-O_2_ inventories were also converted to anomalies by subtracting the long-term mean. To compare the simulated ${{{\rm{O}}}}_{2}^{{{\rm{sat}}}}$ trends with observation-based estimates, we calculate ${{{\rm{O}}}}_{2}^{{{\rm{sat}}}}$ from the EN4 temperature and salinity data^103^ (version EN.4.2.2).

Additionally, in Supplementary Text 2 and Supplementary Figs. 16–18 in the Supplementary Materials, we compare observation-based climatological distributions of dissolved oxygen and ${{{\rm{O}}}}_{2}^{{{\rm{sat}}}}$ against the corresponding model outputs.

Further, we compare the simulated trend in global OHC with observation-based estimates. Specifically, we use the observation-based data products IAPv4^104^, and OHC data from the National Oceanic and Atmospheric Administration (NOAA, updated from Levitus et al.^105^).

### RECCAP2 models

To compare the ORCA025-MOPS simulations with other hindcast simulations, we used nine GOBMs (CESM-ETHZ, CNRM-ESM2-1, EC-Earth3, FESOM-REcoM-LR, MOM6-Princeton, MRI-ESM2-1, NorESM-OC1.2, ORCA025-GEOMAR, and ORCA1-LIM3-PISCES) from RECCAP2^45,106^. Only models evaluated in the model evaluation chapter of RECCAP2 were used, as the remaining models either had drift problems when branching from another simulation with coarser model resolution, had too large salinity biases, or had incomparable historical and control simulations due to their setup^64^. Consistent with the analysis of ORCA025-MOPS, we calculated anomalies in OHC and O_2_ by subtracting the simulation without climate change and variability from the historical simulation with climate change and variability.

### CMIP6 earth system models

Additionally, we have analysed all seven ESMs from CMIP6 to compare hindcast simulations with fully coupled simulations. The ESMs used here are ACCESS-ESM1-5 (ensemble member r1i1p1f1)^107^, CanESM5 (r1i1p1f1), and CanESM5-CanOE (r1i1p2f1)^108^, CNRM-ESM2-1 (r1i1p1f2)^109^, GFDL-CM4 (r1i1p1f1) and GFDL-ESM4 (r1i1p1f1)^110–112^, and MPI-ESM1-2-LR (r1i1p1f1)^113,114^. We used all ESM outputs for which O_2_ and potential temperature were available for the historical simulation, the SSPs 5–8.5 (SSP5–8.5) simulation^115^, and the pre-industrial control simulation. The SSP5–8.5 simulations were used for the years 2015 to 2018 because the historical simulation in CMIP6 stopped after 2014. From 2015 to 2018, SSP5–8.5 was chosen because it is the pathway for which most ESMs provide results. Although it is a high-emission pathway, the radiative forcing in these years is almost identical for all SSPs in the first year, and the marginal differences in radiative forcing are too small to distinguish the climate in the first years between SSPs. Here the anomaly has been calculated as the difference between the pre-industrial control run and the historical run. As the pre-industrial run still simulates internal climate variability, this difference only quantifies the effect of climate change and externally forced variability, such as volcanoes, and not the effect of internal climate variability. The remaining internal climate variability in the anomaly is a superposition of the internal climate variability from the historical simulation and that from the pre-industrial control simulation and has no scientific relevance.

## Supplementary information

Supplementary information

Peer Review File

## Data Availability

A subset of the output data generated in this study is available through GEOMAR at https://hdl:20.500.12085/a4d451d5-a68f-401b-b58d-68792a5a0820^116^. All other model outputs presented here are available upon request. Source data for all figures, including Supplementary Figs., are provided with this paper and are available at the same address [https://hdl.handle.net/20.500.12085/a4d451d5-a68f-401b-b58d-68792a5a0820]. The Earth system model output used in this study is available via the Earth System Grid Federation [https://esgf-node.ipsl.upmc.fr/projects/esgf-ipsl/]. The RECCAP2 model output used in this study is openly accessible in the Zenodo database under record number 7990823. All observational data used for model evaluation are openly available to the public at the following links: Oxygen data from Ito et al.^34^ are available at [https://o2.eas.gatech.edu/data.html] and from GOBAI-O_2_^43^ at [10.25921/z72m-yz67]. Oxygen data from T. Ito^42^ are available from the Biological & Chemical Oceanography Data Management Office (BCO-DMO) at [10.26008/1912/bco-dmo.816978.1] and [https://www.bco-dmo.org/dataset/816978]. EN4 temperature and salinity data^103^ (version EN.4.2.2) were obtained from [https://www.metoffice.gov.uk/hadobs/en4/]. OHC data from NOAA are available at [https://ncei.noaa.gov/access] and from IAPv4^104^ at [www.ocean.iap.ac.cn].

## Code availability

The Python scripts used for all analyses and figures are available through GEOMAR at https://hdl:20.500.12085/a4d451d5-a68f-401b-b58d-68792a5a0820^116^.
