# Supplementary material for: Competing effects of wind and buoyancy forcing on ocean oxygen trends in recent decades
Source: Nat Commun. 2024 Oct 26;15:9264. doi: 10.1038/s41467-024-53557-y (PMC11513149; doi:10.1038/s41467-024-53557-y)
Supplement: Supplementary file 1 — Supplementary information [file 41467_2024_53557_MOESM1_ESM.pdf]

Supplementary information to "Competing effects of wind and buoyancy forcing on ocean oxygen trends in recent decades"

Helene A. L. Hollitzer*[1,2,3], Lavinia Patara[1], Jens Terhaar[2,3], and Andreas Oschlies[1,4]

[1]GEOMAR Helmholtz Centre for Ocean Research Kiel, 24148 Kiel, Germany
[2]Climate and Environmental Physics, Physics Institute, University of Bern, 3012 Bern, Switzerland
[3]Oeschger Centre for Climate Change Research, University of Bern, 3012 Bern, Switzerland
[4]Kiel University, 24118 Kiel, Germany

Corresponding author (*): Helene A. L. Hollitzer (helene.hollitzer@unibe.ch)

S1 Supplementary Methods

S1.1 Additional observational data sets

Oxygen and O_2^{sat} climatologies. For further model evaluation, observational climatological distributions of dissolved oxygen and O_2^{sat} were compared against the corresponding model outputs using the World Ocean Atlas 2023 [1]. Specifically, for comparison we used the 30-year averages (1971-2000 climate normals) of dissolved oxygen and O_2^{sat} as provided by the World Ocean Atlas 2023 (Supplementary Figs. S16, S17, and S18). Data are accessible at www.ncei.noaa.gov/access/world-ocean-atlas-2023/.

Mixed layer depth. To assess simulated mixed layer depths, monthly climatology (2000-2021) mixed layer depth data (m) calculated from Argo profiles were used (Supplementary Fig. S4b), available on a 1° x 1° grid [2]. The climatology is calculated using a hybrid algorithm for the determination of mixed layer depths described in Holte et al. [3]. For comparison against model results (Supplementary Fig. S4c), the climatological field was interpolated onto the remapped ORCA025-MOPS grid and model results were averaged over 2000-2018. In the model, the mixed layer depth is defined as the ocean depth at which sigma-theta has increased by 0.01 kg m^{-3} relative to the near-surface value at 10 metres depth.

AMOC strength To assess changes in the simulated strength of the upper cell of the Atlantic MOC at 26°N, 12-hourly data (since 2004) from the RAPID array [4] were used. Data from the RAPID AMOC monitoring project is funded by the Natural Environment Research Council and are freely available from www.rapid.ac.uk/rapidmoc.

Phytoplankton For the assessment of simulated phytoplankton distribution, monthly climatology (2002-2017) sea surface chlorophyll-a data (mg Chl-a m^{-3}) available at 9 km resolution derived from satellite remote sensing (MODIS-Aqua [5]) were used. After averaging to annual mean, chlorophyll-a was converted to carbon using the algorithm derived by Sathyendranath et al. [6] and to phosphorus assuming a C:P ratio of 117 mol C: 1 mol P. For comparison against simulations, model outputs were averaged over 2002-2017.

Zooplankton To assess the simulated zooplankton distribution, quasi-climatological (including data points between 1932-2010) mesozooplankton biomass data (MARE-DAT [7]) were used, comprising 42,245 data points of monthly mean mesozooplankton biomass (μg C L^{-1}) averaging to annual mean mesozooplankton biomass, total of 23,533 data points were obtained for the upper 200 m. Carbon was converted to phosphorus assuming a C:P ratio of 117 mol C : 1 mol P. For comparison against simulations, model outputs were averaged over 1958-2010. Some zooplankton species perform diel vertical migrations, so that they are more abundant near the surface at night and in mid-water depths (~200-600 m) during the day [8, 9]. This process is not included in the model and may cause an overestimation of the simulated zooplankton biomass in the upper 200 m. Furthermore, the biogeochemical model does not differentiate between micro- and mesozooplankton, but aggregates both types into a single component. Due to sparse data for microzooplankton biomass, and following the approach adopted by Chien et al. [10], a fixed approximate micro-to-mesozooplankton ratio of one was assumed, thus doubling the observed concentrations of mesozooplankton biomass for comparison with model results.

S1.2 MOC strength computation (model)

The MOC stream function can be expressed in both depth or density coordinates. While the MOC calculated in density coordinates is better suited to characterise water mass transports, in the North Atlantic, the description in depth coordinates is still often used.

For Supplementary Fig. S10a, the global MOC stream function was computed based on monthly output in density coordinates following the methodology outlined by Farneti et al. [11] and using the `cdfmocsig` routine of `CDFTOOLS` (https://github.com/meom-group/CDFTOOLS). Meridional volume transports are classified according to potential density classes referenced to 2,000 m depth (σ_2). The time series in Supplementary Fig. S10a are obtained by first computing an annual average of the stream function and then taking the maximum MOC stream function value between 35°S and 60°S and between σ_2 levels of 34.4750 - 37.4750 kg m^{-3}. The time series therefore provides a measure of the strength of the clockwise (positive) upper cell of the MOC in the Southern Ocean.

For Supplementary Fig. S10b, we take the maximum 26.5°N of the annually averaged AMOC stream function computed in depth coordinates. This diagnostic is a measure of the strength of the upper cell of the AMOC and is directly relatable to the RAPID array (https://rapid.ac.uk).

S2 Model performance

The climatology of oceanic oxygen (O_2) concentration simulated by ORCA025-MOPS HIND (averaged over the two sets of experiments; see Methods) is evaluated using the observational data product World Ocean Atlas 2023 [1]. ORCA025-MOPS simulates a reasonable mean state compared to the World Ocean Atlas 2023 (Supplementary Figs. S16 and S17). It generally reproduces the observed pattern of sea surface O_2 concentrations, largely reflecting accurately simulated O_2^{sat} (Supplementary Fig. S18), which is primarily determined by temperature and secondarily by salinity. Exceptions are (1) overestimated surface O_2 in the North Atlantic, which may be caused by errors in the representation of Gulf Stream dynamics, with the North Atlantic Current being too zonal [12] and (2) underestimated surface O_2 in the Arctic Ocean (Supplementary Figs. S16d and S18b), potentially due to unrealistically deep vertical mixing resulting in an excessively high surface salinity in the Arctic Ocean [13, 14].

In contrast to the general agreement at the sea surface, discrepancies between simulated and observed O_2 concentrations increase in the subsurface and deep ocean (Supplementary Fig. S17d-f). Interior O_2 biases include an underestimation in mode and intermediate waters, an overestimation in deep water masses, and an underestimation in bottom waters, especially in the Atlantic Basin. In the ocean interior, the origin of the model bias is more complex, with contributions from both ocean circulation and particulate organic carbon flux [15]. While the observed O_2 concentration minimum averages about 1,000 m depth, the model shows a shallower minimum at about 500 m depth. This behaviour has been noted in other models (e.g. Bao et al. [15]) and may be attributed to a misrepresentation of remineralisation processes with overly high vertical attenuation of the particulate organic carbon flux.

The positive bias in deep waters, which are dominated by North Atlantic Deep Water (NADW), may result from overestimated mixed layer depths at subpolar latitudes of the North Atlantic (with respect to ARGO observations) increasing the injection of O_2 into the deep ocean (Supplementary Fig. S4c). Since surface ocean O_2 is overestimated in the North Atlantic (see above), this may add to the bias. On the other hand, the transport of the Atlantic Meridional Overturning Circulation (AMOC) at 26°N (around 13 Sv) is not overestimated with respect to the RAPID array [16]. The positive O_2 anomaly in NADW may also propagate into the Southern Ocean, where it might contribute to the overestimated O_2 concentrations in the deep water masses and, once upwelled at the subpolar divergence, to the overestimates in the subpolar upwelling regions (Supplementary Fig. S17). This may limit the simulated deoxygenation of the Southern Ocean in the model. The Southern Ocean is also characterised by underestimated O_2 concentrations in

intermediate water masses, possibly due to a sluggish upper cell of the Meridional Overturning Circulation (MOC) compared to reality. Conversely, the mixed layer depth is generally overestimated, especially in the southeast Pacific (Supplementary Fig. S4c), and thus cannot explain the underestimated O_2 concentrations along the Antarctic Intermediate Water pathway.

Finally, the biased mean conditions are also the result of underlying spurious drifts, which depend critically on the choice of the stoichiometric O_2:P ratio (Supplementary Fig. S15).

Supplementary figures

Source data for all figures, including the supplementary figures contained in this document, are supplied with this paper and are available through GEOMAR at hdl:20.500.12085/a4d451d5-a68f-401b-b58d-68792a5a0820 [17].

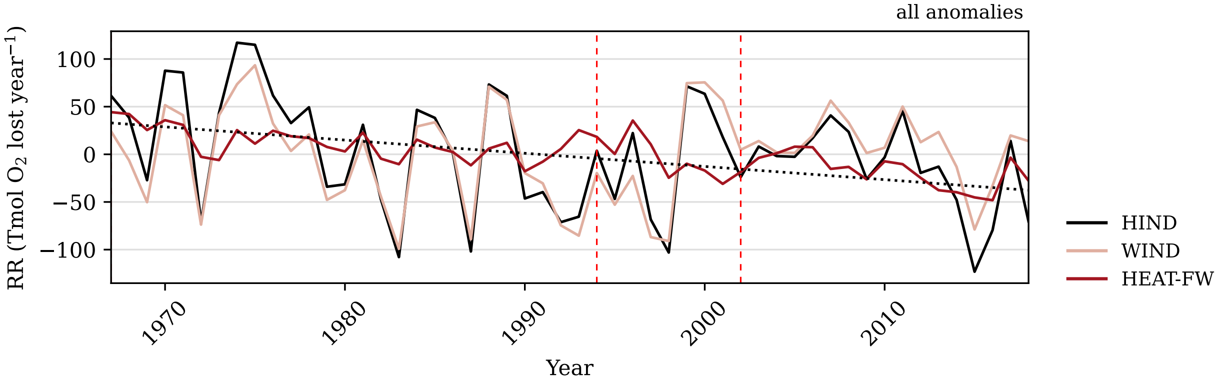

Figure S1: Change in global remineralisation rate in the hindcast and sensitivity experiments. Time series (1967-2018) of globally integrated remineralisation rate (RR) anomalies in ORCA025-MOPS HIND (black), WIND (rose), and HEAT-FW (red). Data are integrated until the ocean bottom and shown as anomalies with respect to the 1967-2018 mean. The black dotted line shows the linear regression for the HIND data fit between 1967 and 2018 (slope ± se = -1.38 ± 0.52, Adj. $R^2 = 0.108$, $F(1,50) = 7.199$, p = 0.01). Red dashed lines delineate the three periods of the oxygen inventory trajectory described in the Results section of the main text.

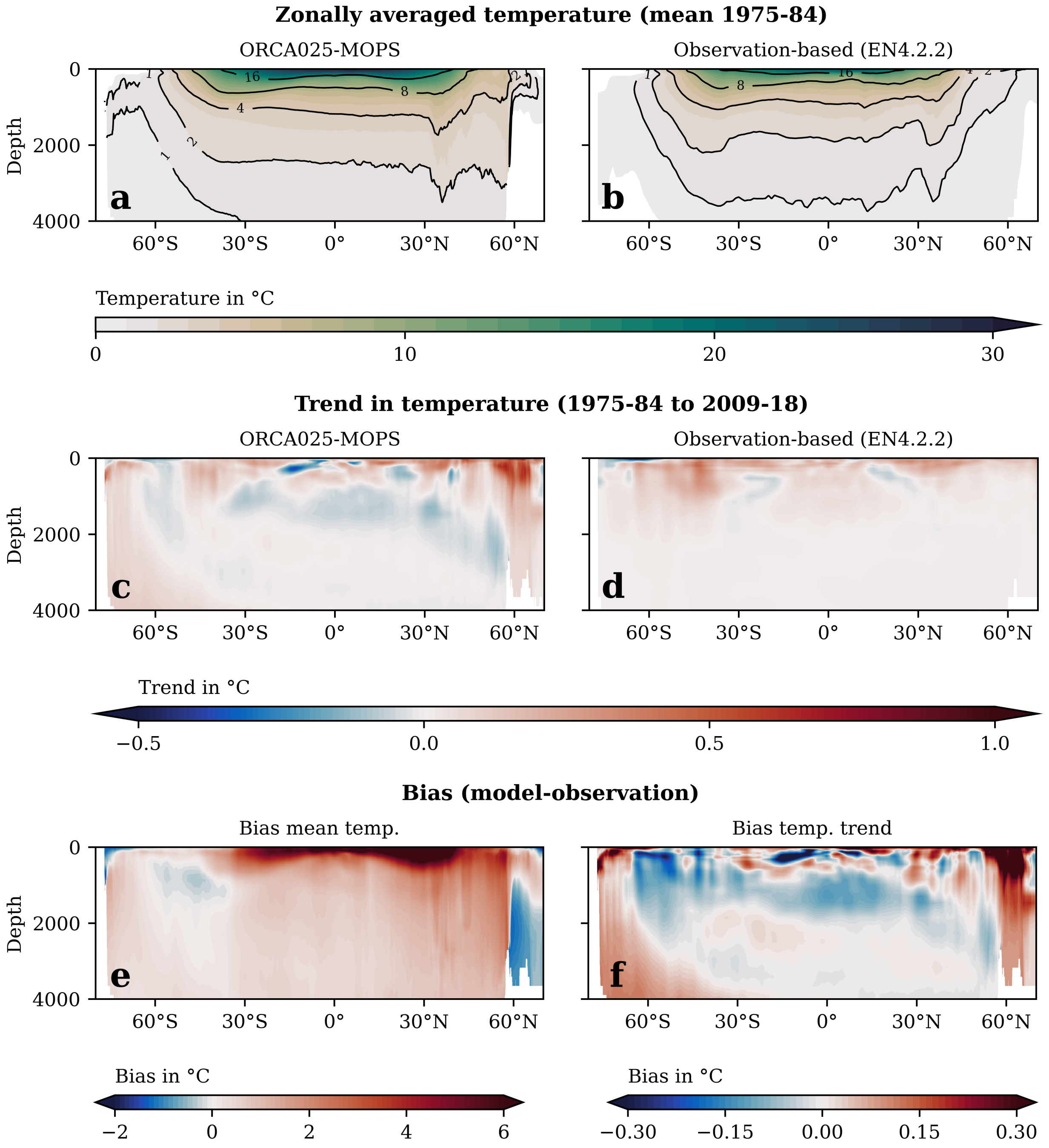

Figure S2: Ocean temperature and trends simulated by the model compared to observation-based data. Meridional sections of (a-b) ocean temperature and (c-d) trends in ocean temperature (1975-84 versus 2009-18) for the hindcast experiment (HIND) and observation-based data from EN4 [18] (version 4.2.2). Panels (e) and (f) show the biases in ocean temperature and ocean temperature trends, respectively.

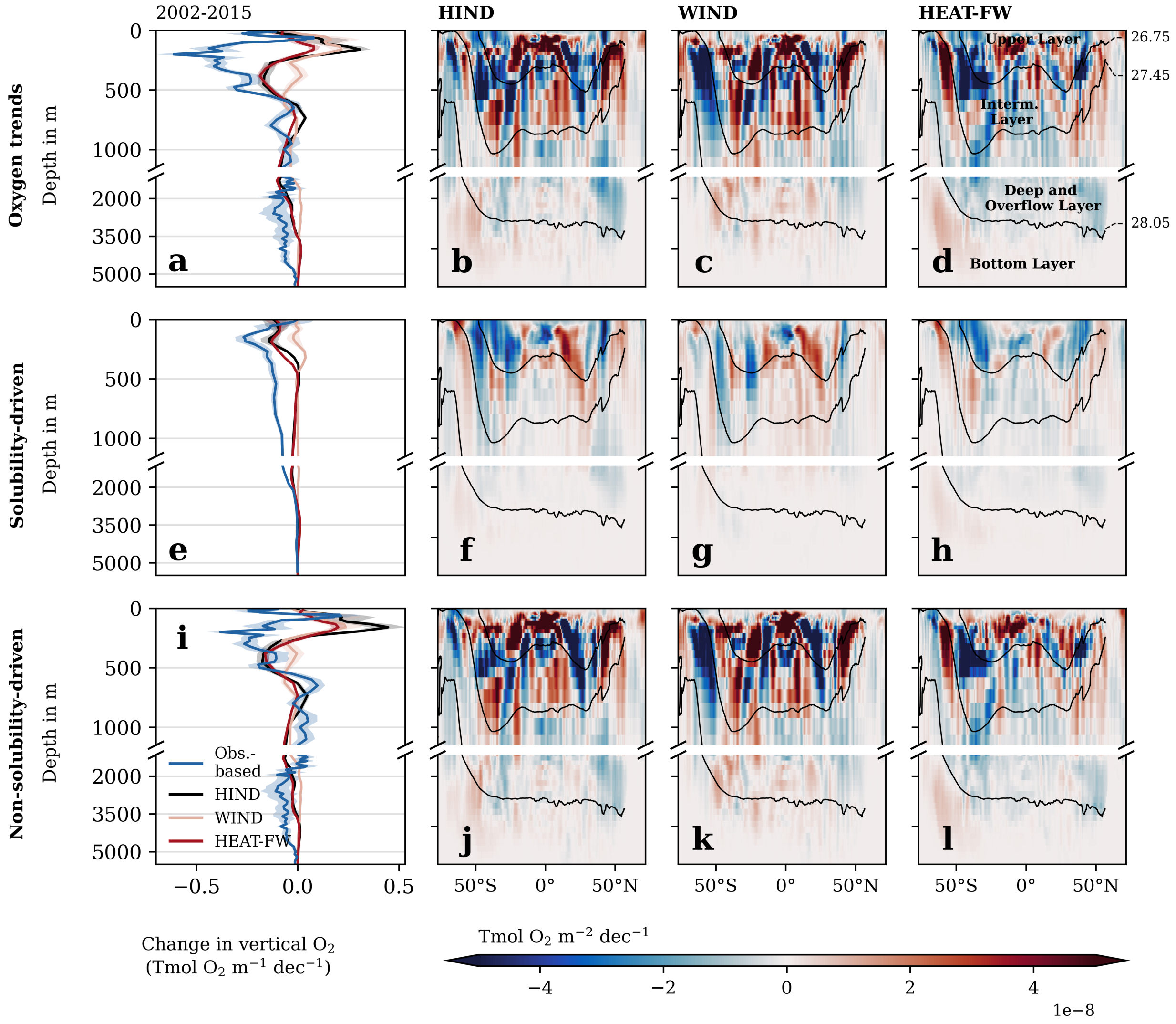

Figure S3: Ocean oxygen trends from 2002-2018 by depth and latitude. The upper panels show linear trends in oxygen content as a function of (a) depth and (b-d) as a function of depth and latitude. The panels below show the same as (a-d), but decompose the oxygen change into its (e-h) solubility-driven (O_2^{sat}) and (i-l) non-solubility-driven components. Trends are shown for the hindcast (HIND), the sensitivity experiments WIND and HEAT-FW, and for the observation-based data products Ito-22 [19] for oxygen (1967-2015) and EN4.2.2 [18] for O_2^{sat} (and their combination O_2^{total} - O_2^{sat} to compute the non-solubility-driven component). The shading in the line graphs indicates the standard error of the estimated linear least-squares regression slopes and black contour lines show the the neutral density surfaces γ^n = 26.75, 27.45, and 28.05 kg m^{-3}.

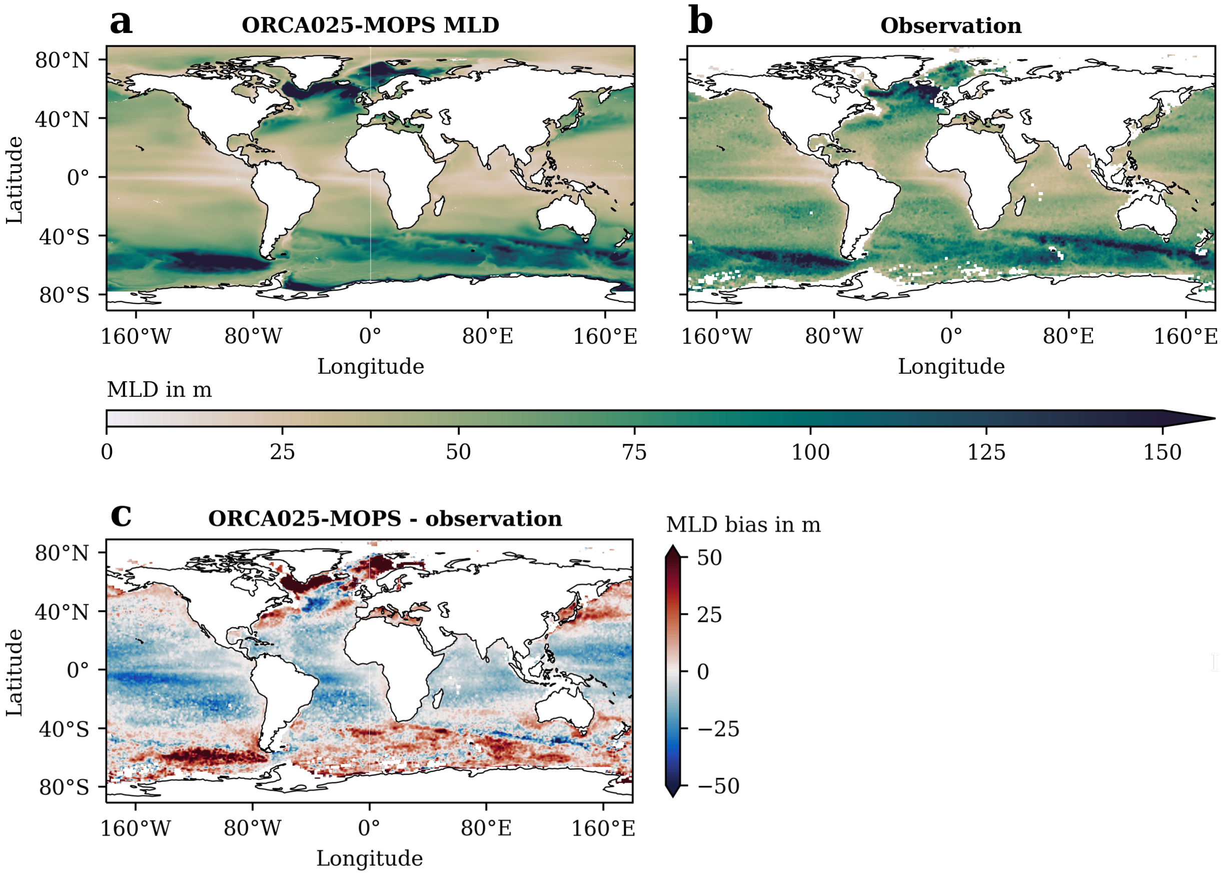

Figure S4: Mixed layer depth climatology maps simulated by the model compared to observation-based data. Mixed layer depth climatology in (a) ORCA025-MOPS HIND from 2000 to 2018 and (b) corresponding observational data from 2000 to 2021. The differences between model and observations are shown in (c) The observational data are derived from Argo profiles [2], with additional details in Section S2.3.

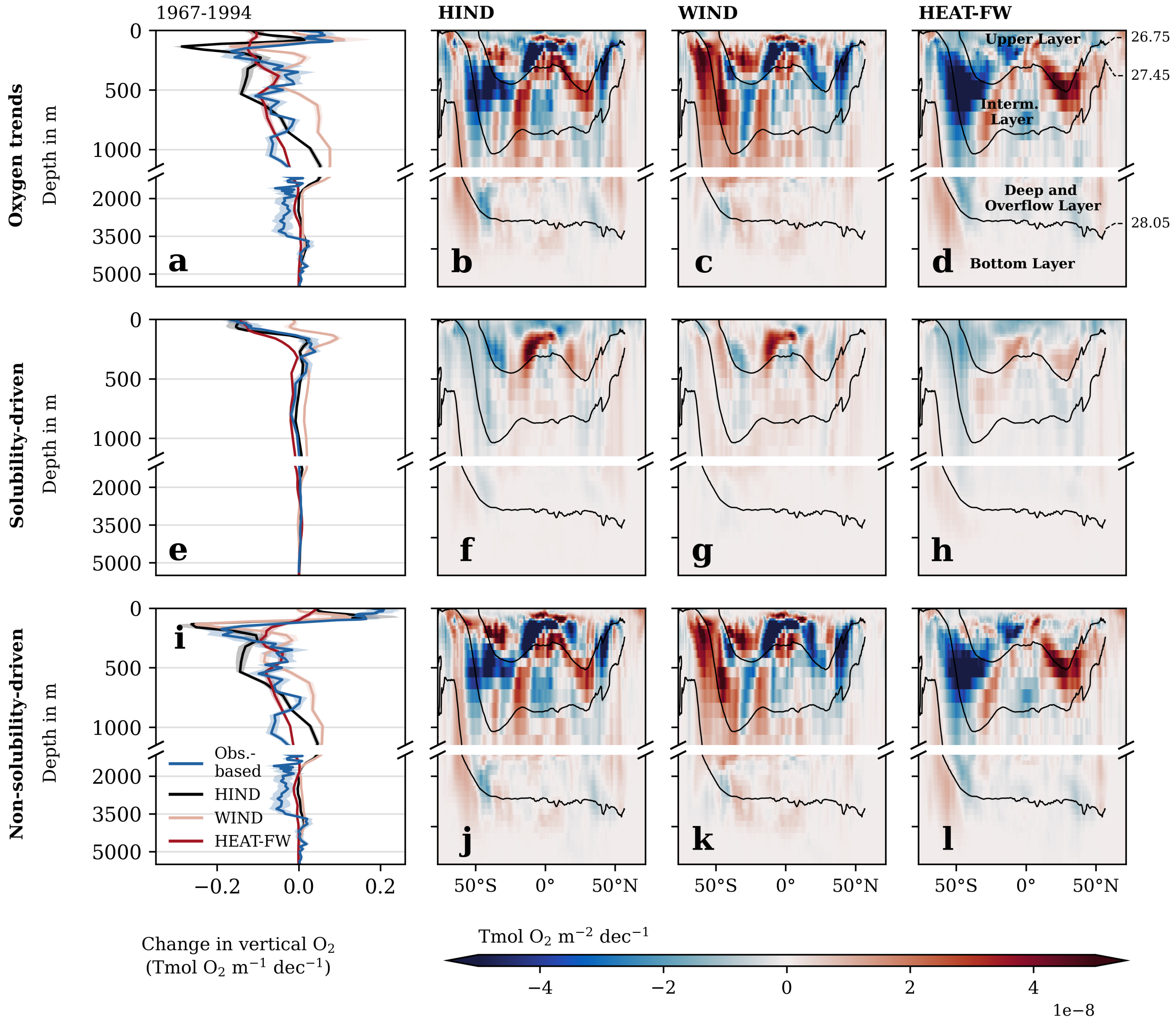

Figure S5: Ocean oxygen trends from 1967-1994 by depth and latitude. Same as Supplementary Fig. S3, but for the period 1967-1994. The upper panels show linear trends in oxygen content as a function of (a) depth and (b-d) as a function of depth and latitude. The panels below show the same as (a-d), but decompose the oxygen change into its (e-h) solubility-driven (O_2^{sat}) and (i-l) non-solubility-driven components. Trends are shown for the hindcast (HIND), the sensitivity experiments WIND and HEAT-FW, and for the observation-based data products Ito-22 [19] for oxygen (1967-2015) and EN4.2.2 [18] for O_2^{sat} (and their combination O_2^{total} - O_2^{sat} to compute the non-solubility-driven component). The shading in the line graphs indicates the standard error of the estimated linear least-squares regression slopes and black contour lines show the the neutral density surfaces γ^n = 26.75, 27.45, and 28.05 kg m^{-3}.

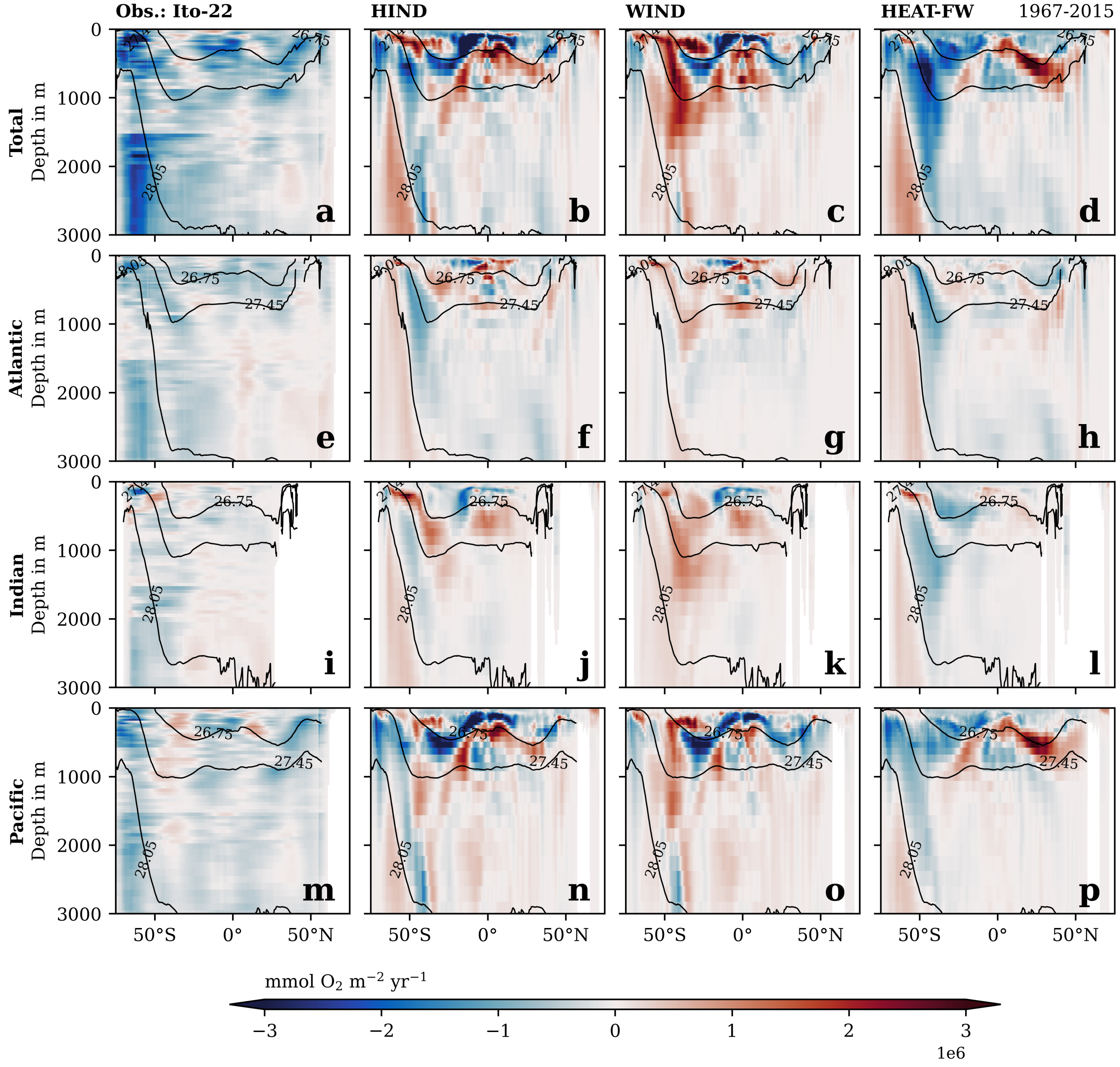

Figure S6: Ocean oxygen trends by depth and latitude, separated by basin. Trends in ocean oxygen during the deoxygenation period 1967-2015 by depth and latitude. The top panels (a-d) show linear trends in oxygen content integrated over all longitudes. The panels below show trends separately for (e-h) 75°W to 25°E (corresponding approximately to the Atlantic Ocean), (i-l) 25°E to 125°E (corresponding approximately to the the Indian Ocean), and (m-p) 125°E to 75°W (corresponding approximately to the Pacific Ocean). Trends are shown for observation-based data developed by Ito et al.[19], the hindcast experiment (HIND), and the sensitivity experiments WIND and HEAT-FW. Contours show the the neutral density surfaces γ^n = 26.75, 27.45, and 28.05 kg m^{-3} for each respective basin (simulated by HIND).

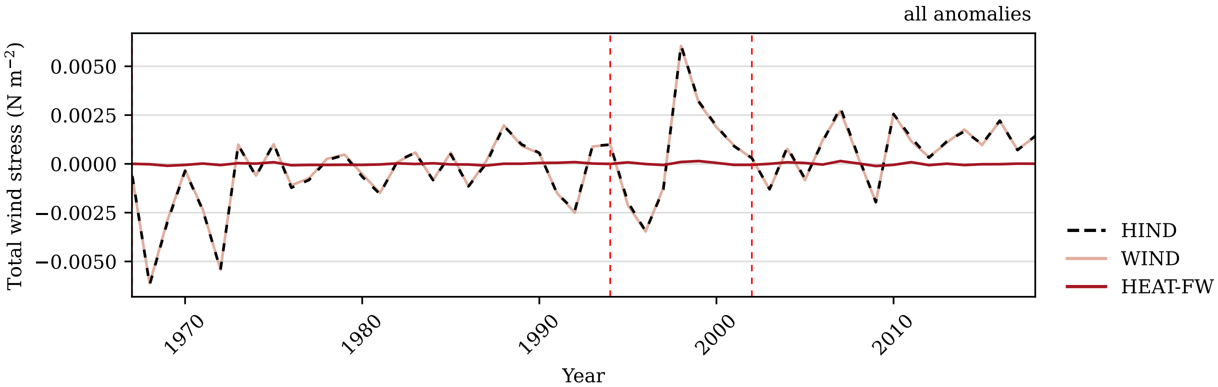

Figure S7: Change in globally averaged wind stress in the hindcast and sensitivity experiments. Time series (1967-2018) of globally averaged total wind stress anomalies in ORCA025-MOPS HIND (black), WIND (rose), and HEAT-FW (red). Data are mean-centered with respect to the 1967-2018 mean. Red dashed lines delineate the three periods of the oxygen inventory trajectory described in the Results section of the main text.

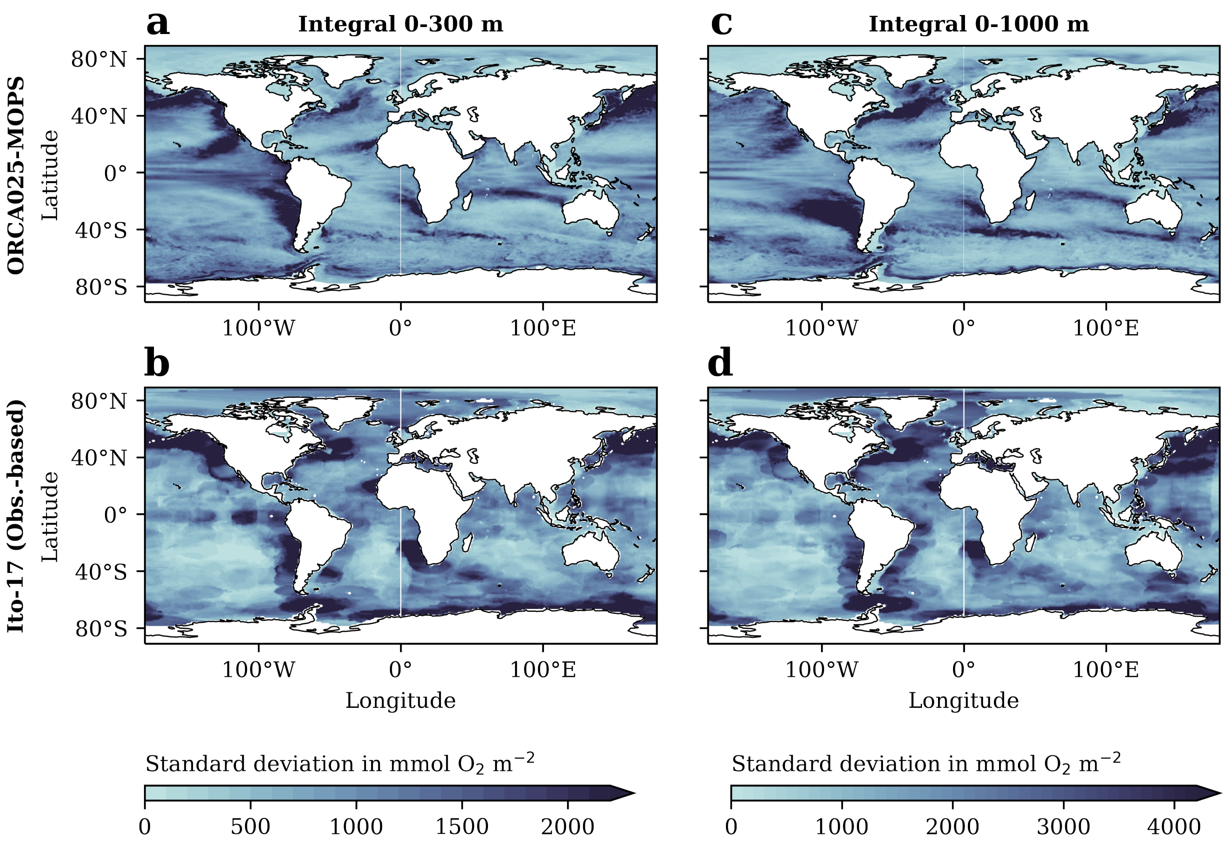

Figure S8: Oxygen variability maps in the hindcast experiment and comparable observation-based data. Variability in dissolved oxygen from 1967-2010. Dissolved oxygen variability at each grid point is estimated by the standard deviation of the high-pass filtered (15-point running mean [window function: Hanning window] subtracted) annual O_2 anomaly time series (1967-2010), integrated to (a-b) 300 m depth and (c-d) 1,000 m depth. Estimates are shown for ORCA025-MOPS (HIND) and the observation-based product Ito-17 [20].

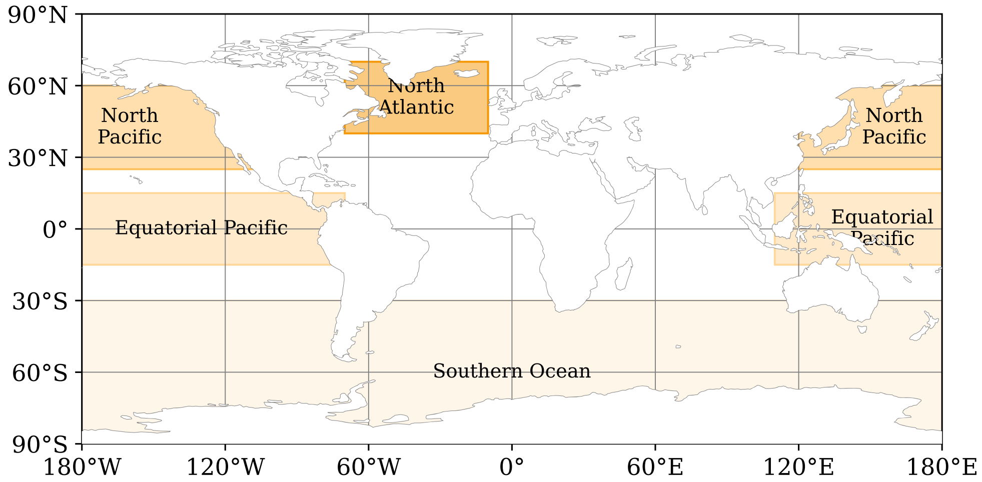

Figure S9: Overview of the sub-region boundaries. The sub-regions are delimited as follows: (1) the North Atlantic Ocean: 40°N to 70°N; 70°W to 10°W, the equatorial Pacific Ocean: 15°S to 15°N; 110°E to 70°W, the Southern Ocean: extending from the Antarctic coastline to 30°S, and (4) the North Pacific Ocean: 25°N to 60°N; 110°E to 100°W.

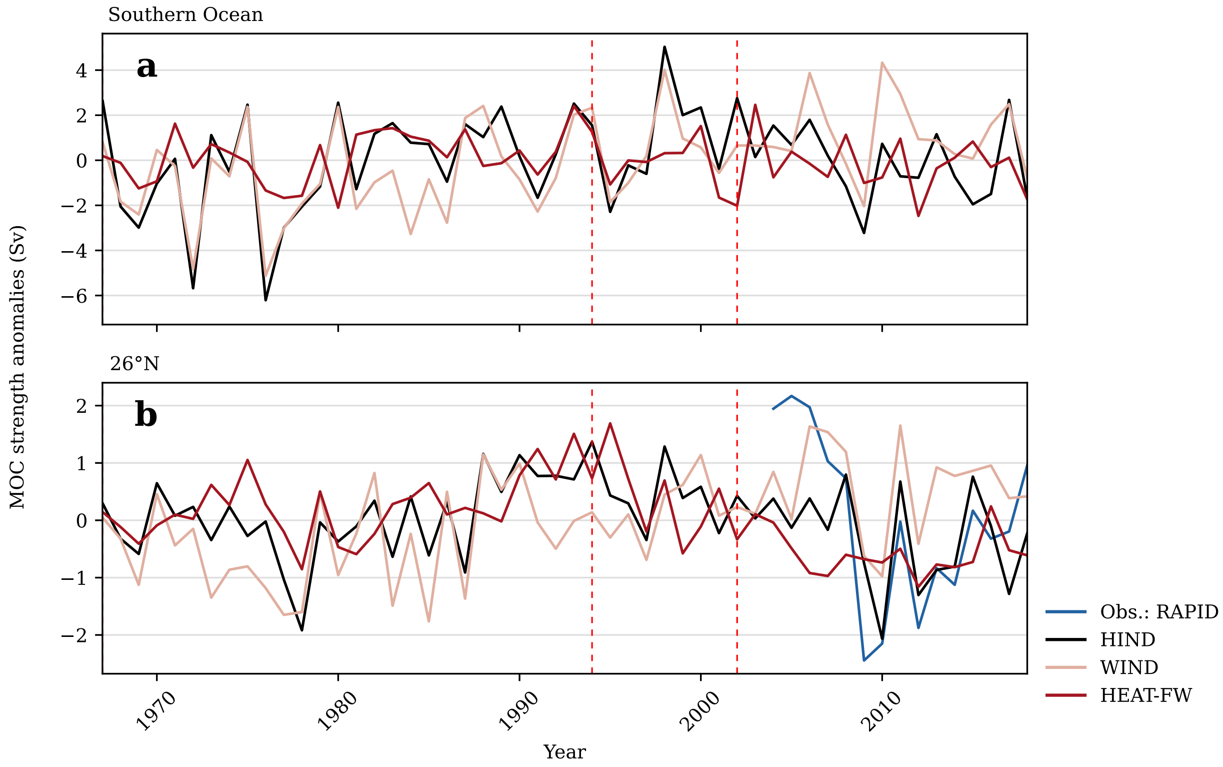

Figure S10: Change in meridional overturning circulation strength in hindcast and sensitivity experiments, as well as observations. Time series (1967-2018) of (a) the strength of the clockwise upper cell of the meridional overturning circulation (MOC) in the Southern Ocean and (b) the strength of the upper cell of the Atlantic MOC at 26°N in ORCA025-MOPS HIND (black), WIND (rose), HEAT-FW (red), and observations from the RAPID AMOC monitoring project [4] in blue. For information on the computation of the MOC strength, see Section S2.1 above. Data are shown as anomalies and mean-centred with respect to the 1967-2018 mean. Red dashed lines delineate the three periods of the oxygen inventory trajectory described in the Results section of the main text. Values are in Sverdrup (Sv) $= 10^6 \frac{m^3}{s}$.

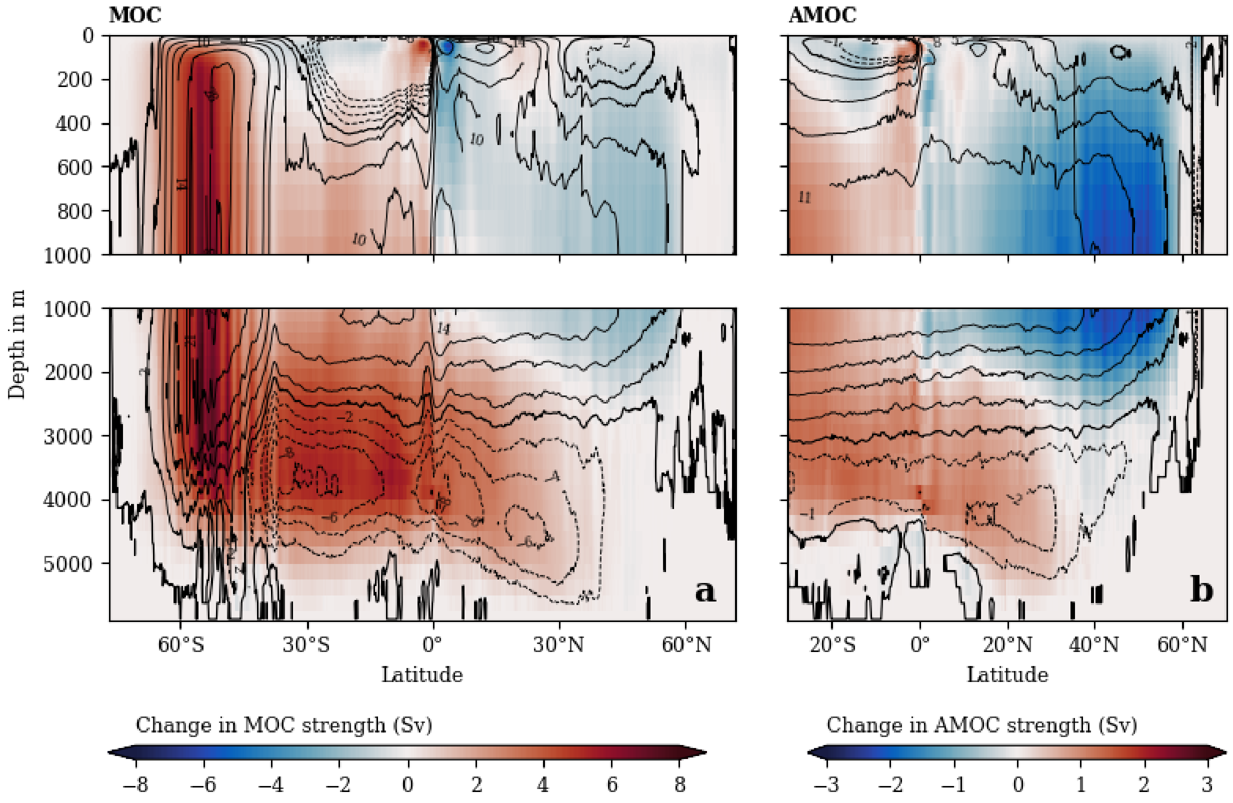

Figure S11: Trend in meridional overturning circulation strength in hindcast experiment. Change between 1965-1974 and 2009-2018 of (a) the global meridional overturning circulation (MOC) and (b) the Atlantic MOC in ORCA025-MOPS HIND. Positive values indicate a clockwise circulation. The contours show the mean values between 2009 and 2018. For information on the computation of the MOC strength, see Section S2.1 above. Values are in Sverdrup (Sv) = $10^6 \frac{m^3}{s}$.

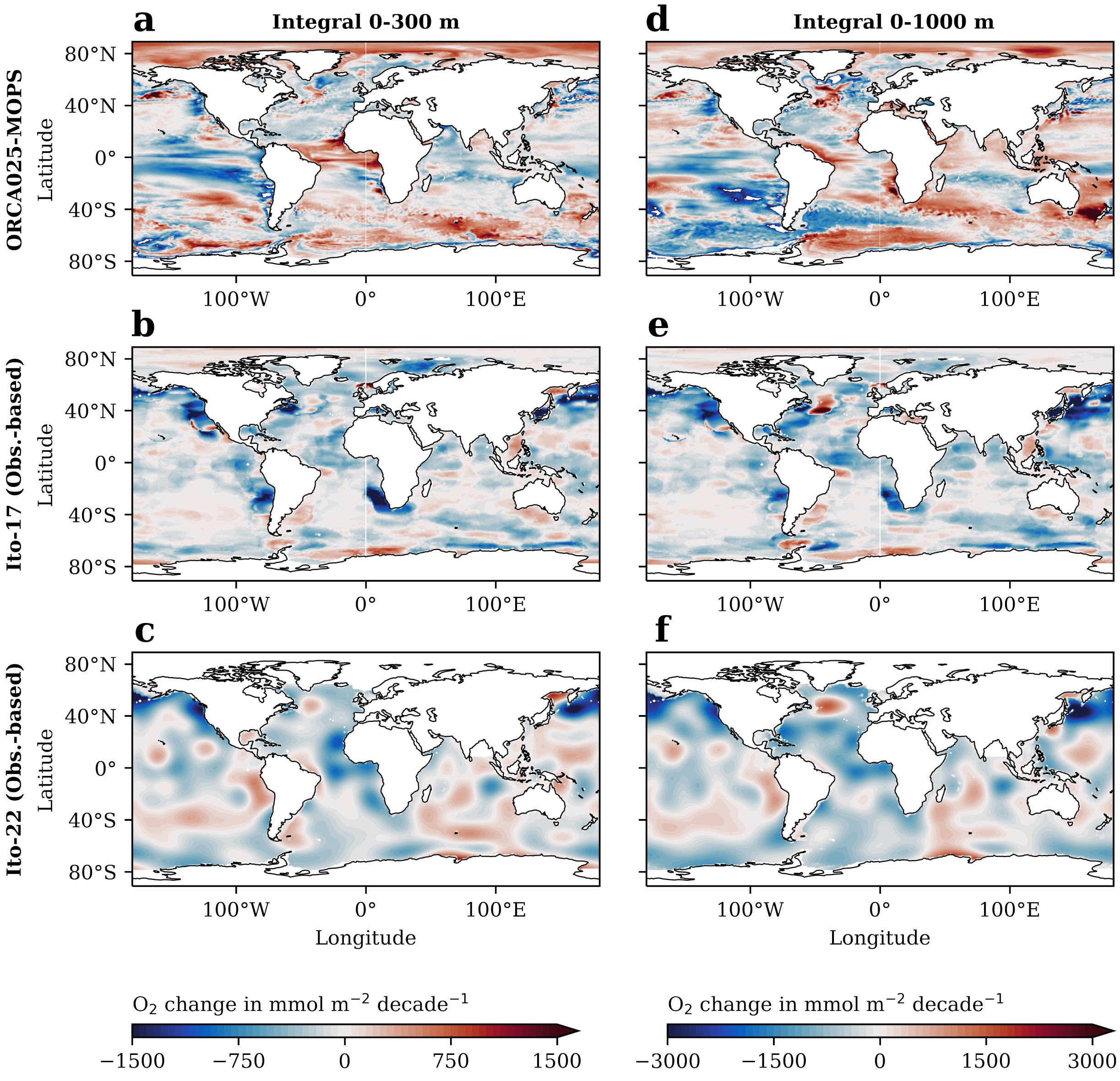

Figure S12: Oxygen trend maps in the hindcast experiment and comparable observation-based data. Linear trends in dissolved oxygen (1967-2010) from model and observation-based estimates integrated to (a-c) 300 m depth and (d-f) 1,000 m depth. Estimates are shown for ORCA025-MOPS (HIND), and the observation-based products Ito-17 [20] and Ito-22 [19].

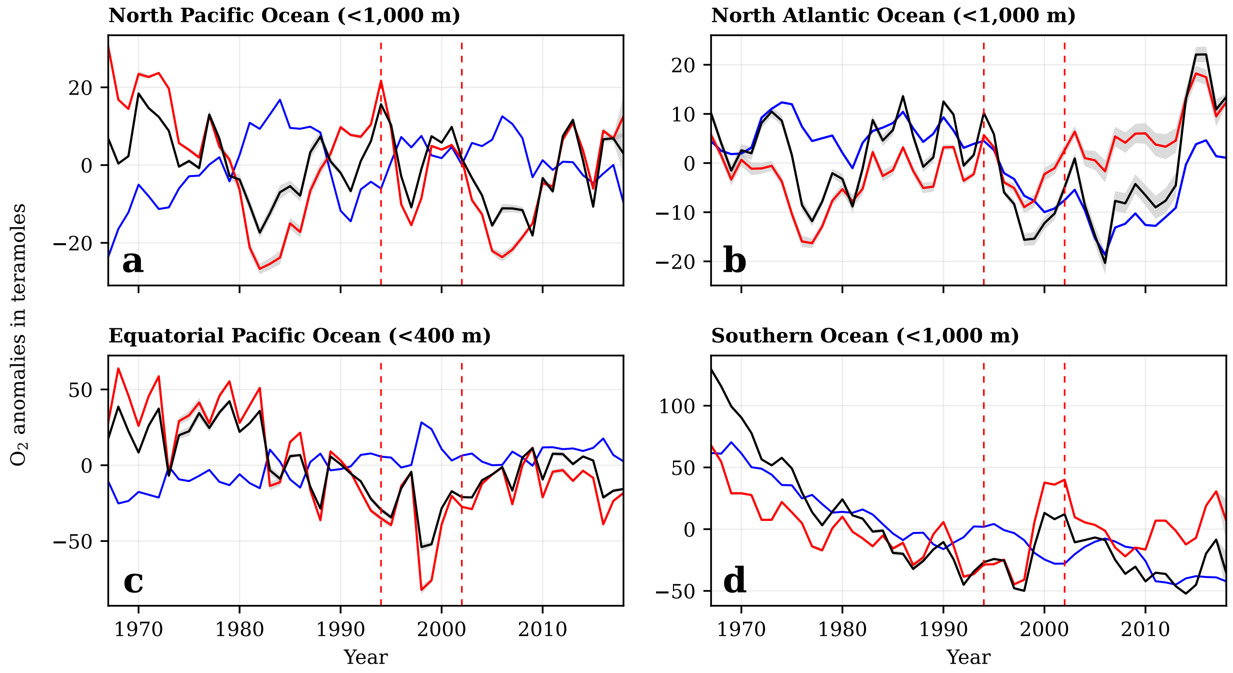

Figure S13: Changes in regional ocean oxygen, oxygen at saturation and its residual in the hindcast experiment. Time series of O_2 anomalies (black), O_2^{sat} anomalies (blue), and the residual between total oxygen anomalies and O_2^{sat} anomalies, i.e. non-solubility-driven changes (red) in HIND for (a) the North Pacific Ocean, (b) the North Atlantic Ocean, (c) the equatorial Pacific Ocean, and (d) the Southern Ocean. The lines represent averages over the two sets of experiments (see Methods), with the shading indicating the range between the minimum and maximum estimates. No uncertainty is computed for the O_2^{sat} anomalies, as each pair of experiments share the same physics and do not differ in their O_2^{sat} estimates. All data are mean-centred using the 1967-2018 long-term mean. Red dashed vertical lines delineate the three periods of the oxygen inventory trajectory described in the main text. Note the different y-axis scales in the different panels.

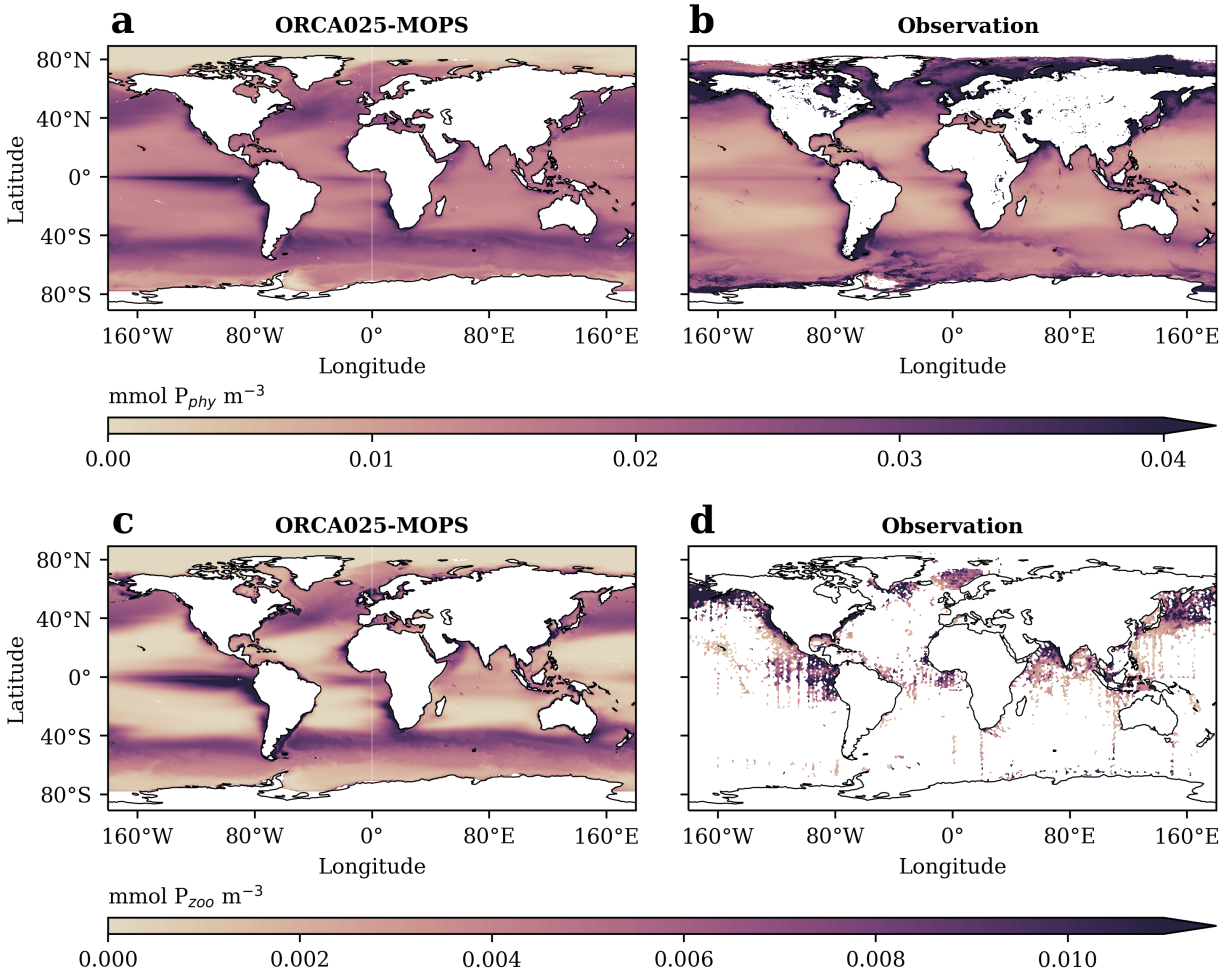

Figure S14: Distribution of phyto- and zooplankton in the hindcast experiment and comparable observation-based data. Organic tracer concentrations for (a-b) the first layer ($\sim$ 6 m) of phytoplankton and (c-d) 0–200 m of zooplankton climatologies from 2002-2017 and 1932-2010 (1958-2010 for model results), respectively, in HIND and observation-based data. Observation-based data of phytoplankton and zooplankton are derived from chlorophyll-a (MODIS-Aqua [5]) and mesozooplankton (MAREDAT [7]), respectively. For details of the observation-based data, including details on the conversion to phosphorus units, refer to Section S1.1 above.

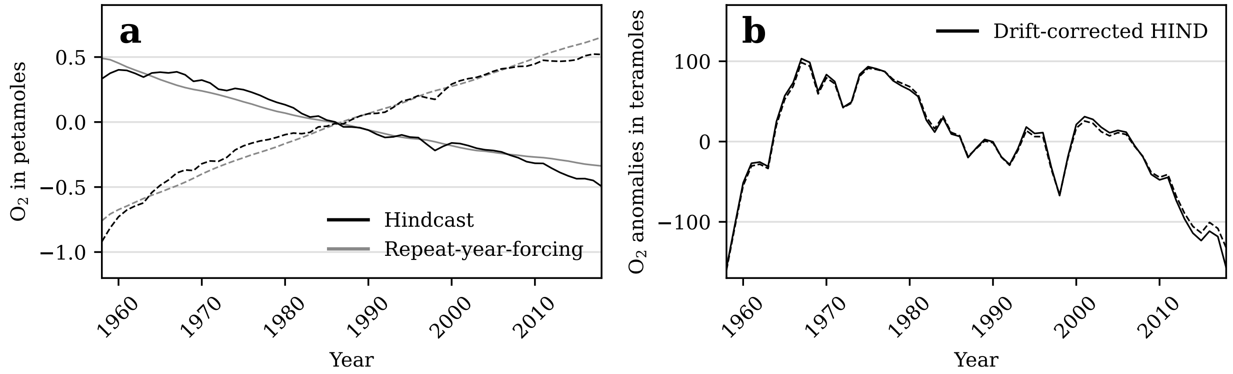

Figure S15: Spurious drift elimination procedure. Visualisation of the spurious drift elimination procedure using the repeat-year-forcing simulations. (a) Time series (1958-2018) of the global oceanic oxygen inventory in the two hindcast (HIND; black) and repeat-year-forcing (RYF; grey) experiments. The two RYF-HIND sets differ in their configuration of biogeochemical parameters, namely their stoichiometric O_2:P ratio. The runs with an O_2:P ratio of 150 are shown as solid lines and the dashed lines are the estimates from the runs with an O_2:P ratio of 162. (b) Global O_2 inventory anomalies relative to the RYF experiments, obtained by the grid-point-wise subtraction of the O_2 estimates in RYF from that in HIND.

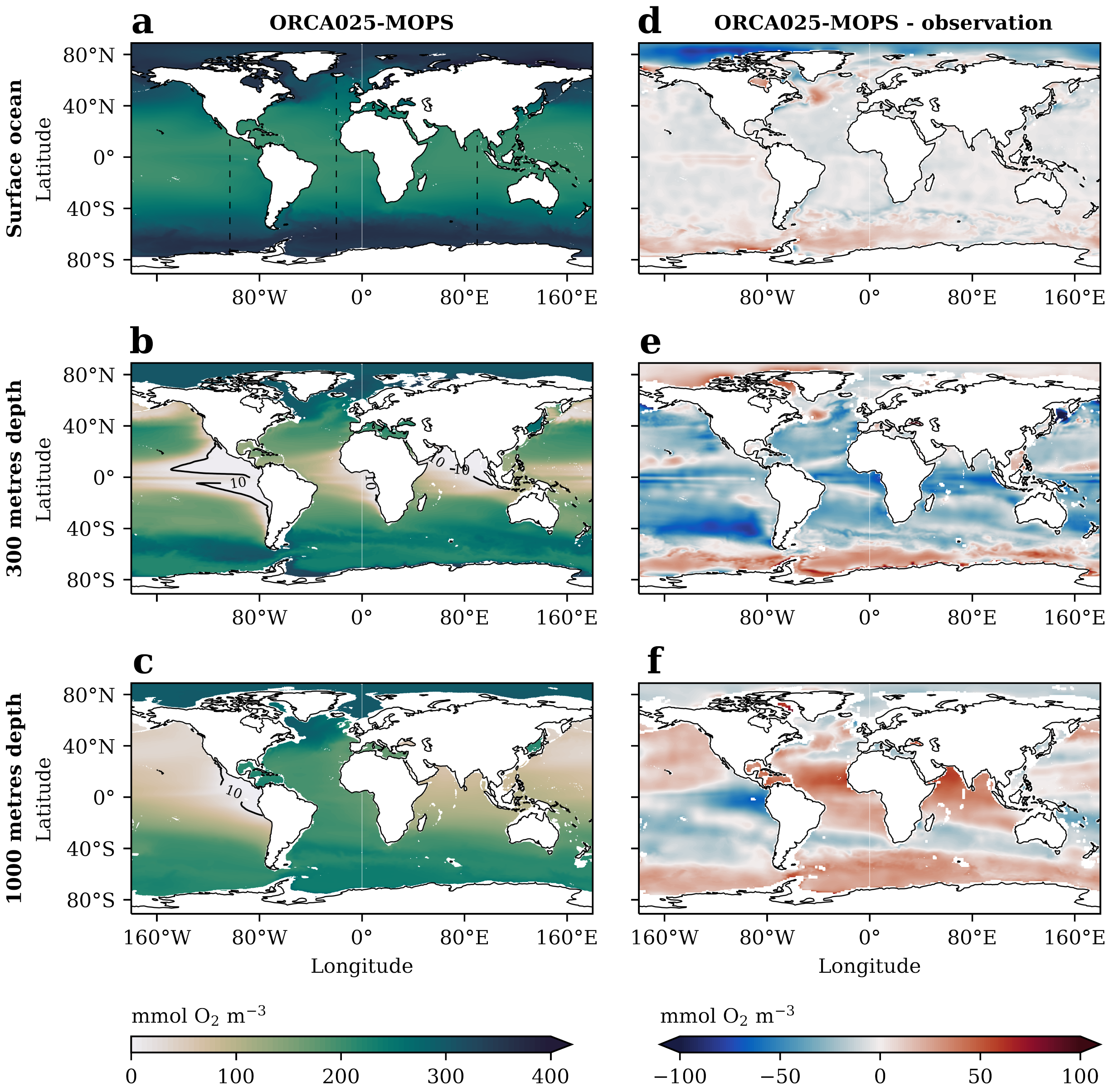

Figure S16: Ocean oxygen climatology maps simulated by the model compared to observation-based data. O_2 climatology from 1971-2000 in ORCA025-MOPS HIND for (a) the surface ocean, (b) at 300 m, and (c) at 1,000 m depth. The corresponding differences between model and observations are shown in (d-f), respectively. The observation-based data used are from the World Ocean Atlas 2023 [1], accessible at www.ncei.noaa.gov/access/world-ocean-atlas-2023/. Contour lines in (a-c) correspond to an O_2 concentration of 10 mmol m^{-3}. The dashed lines in (a) indicate the sections shown in Supplementary Fig. S17 below.

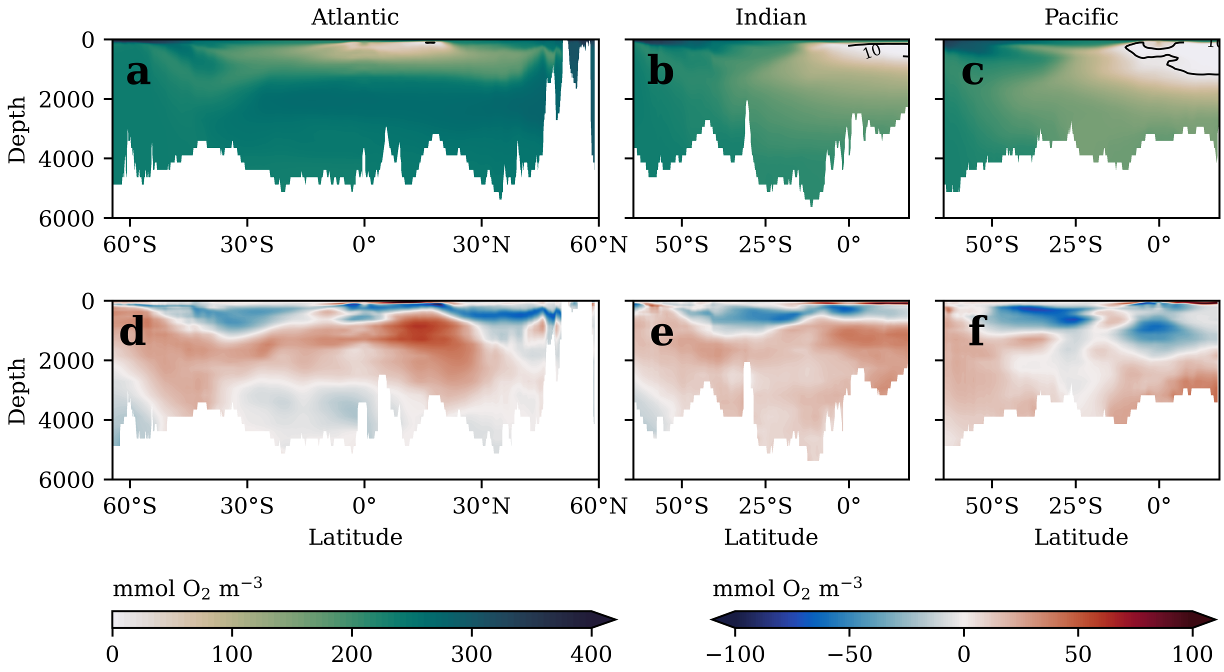

Figure S17: Ocean oxygen climatology sections simulated by the model compared to observation-based data. O_2 climatology from 1971-2000 in ORCA025-MOPS HIND across the (a) Atlantic (20°W), (b) Indian (90°E), and (c) Pacific (103°W) Oceans. The corresponding differences between model and observations are shown in (d-f), respectively. The observation-based data used are from the World Ocean Atlas 2023 [1], accessible at www.ncei.noaa.gov/access/world-ocean-atlas-2023/. Contour lines in (a-c) correspond to an O_2 concentration of 10 mmol m^{-3}.

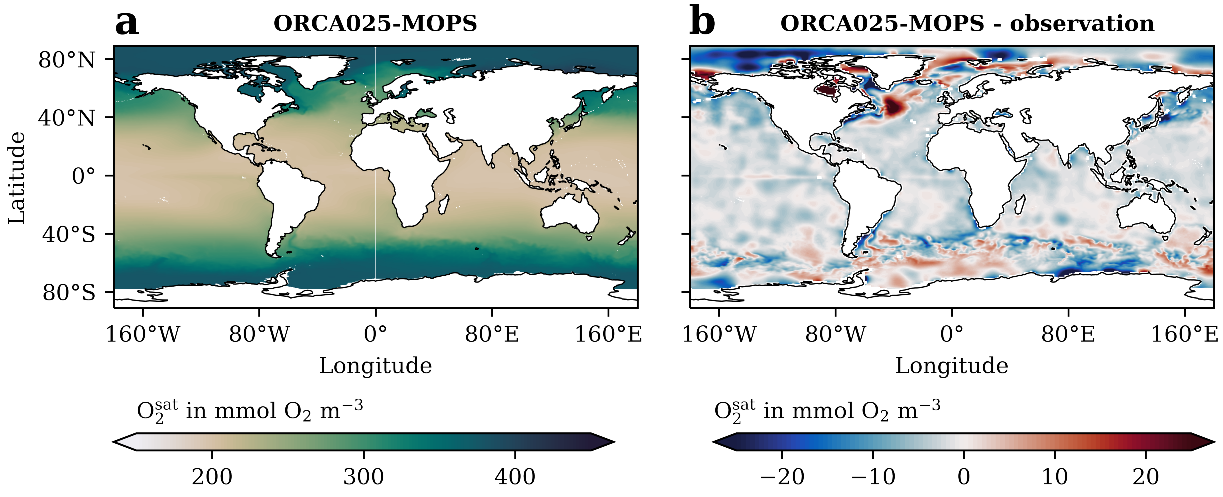

Figure S18: Ocean oxygen at saturation climatology map simulated by the model compared to observation-based data. (a) Surface ocean O_2^{sat} climatology from 1971-2000 in ORCA025-MOPS HIND and (b) the corresponding difference between model and observation-based data from the World Ocean Atlas 2023 [1], accessible at www.ncei.noaa.gov/access/world-ocean-atlas-2023/.

Supplementary tables

Table S1: Change in globally integrated oceanic O_2 and O_2^{sat} in teramoles per decade (± standard error) over 1967-1994, 2002-2018, and 1967-2018[a]. Estimates are given for the hindcast experiment (HIND) and for the sensitivity experiments WIND and HEAT-FW. Details of the linear regression results are given in Tables S5 and S6 for O_2 and O_2^{sat} respectively.

**Period**	**Experiment**	$\mathbf{O_2}$ **trend** (Tmol dec^{-1})	$\mathbf{O_2^{sat}}$ **trend** (Tmol dec^{-1})
1967-1994	HIND	-46.41 ± 4.95	-1.89 ± 2.77
	WIND	68.99 ± 4.253	36.86 ± 1.86
	HEAT-FW	-96.18 ± 4.57	-28.46 ± 2.24
2002-2018	HIND	-116.75 ± 6.58	-69.75 ± 2.72
	WIND	25.45 ± 6.10	6.86 ± 2.54
	HEAT-FW	-179.50 ± 5.70	-76.66 ± 3.79
1967-2018	HIND	-35.04 ± 2.84	-11.96 ± 2.19
	WIND	64.11 ± 1.93	27.39 ± 1.35
	HEAT-FW	-93.55 ± 2.71	-29.86 ± 1.43

[a] Note that the varying length of the different time periods, which reflect major shifts in the global ocean oxygen trend, affect the uncertainties in the reported oxygen trends, as shorter periods are more susceptible to noise and outliers at the beginning or end of the period.

Table S2: Total oxygen inventory changes (Tmol) in the whole water column, the upper 1,000 m and below 1,000 m for four time periods, with percentages indicating the contribution of each layer to the total change.

**Period**	**Full Water Column (Tmol)**	**Upper 1,000 m (Tmol)**	**Below 1,000 m (Tmol)**
1967 to 1994	-84.98	-192.92 (226.95%)	107.94 (-127.04%)
1994 to 1998	-82.83	-112.74 (136.11%)	29.91 (-36.10%)
1998 to 2002	92.10	100.17 (108.75%)	-8.06 (-8.75%)
2002 to 2018	-169.85	-57.79 (34.03%)	-112.05 (65.97%)

Table S3: Change in observation-based, globally integrated oceanic O_2 (Ito-22 [19]) and O_2^{sat} (EN4.2.2 [18]) in teramoles per decade (± standard error) over 1967-1994 and 1967-2015[a]. Intercepts for the ordinary least squares regressions are not shown.

**Period**	**Data**	**Estimate ± se**[b]	**p-value**	$\mathbf{R^2_{adj.}}$	**F-statistic (df**[c]**)**
1967-1994	Obs.-based: O_2 (Ito-22)	-108.2 ± 60.6	.086	.075	3.190 (1, 26)
	Obs.-based: O_2^{sat} (EN4)	2.28 ± 9.42	.810	-0.036	0.05883 (1, 26)
2002-2015	Obs.-based: O_2 (Ito-22)	-460.4 ± 156.9	.013	.369	8.609 (1, 12)
	Obs.-based: O_2^{sat} (EN4)	-209.8 ± 17.26	.000	.919	147.7 (1, 12)

[a] Note that the varying length of the different time periods, which reflect major shifts in the global ocean oxygen trend, affect the uncertainties in the reported oxygen trends, as shorter periods are more susceptible to noise and outliers at the beginning or end of the period.
[b] standard error
[c] degrees of freedom

Table S4: Change in the upper 1,000 m oceanic oxygen inventory in teramoles per decade (± standard error) over 1967-1994 and 2004-2015[a]. Estimates and details of the ordinary least squares regressions are given for the hindcast experiment (HIND) and observation-based data from Ito-17 [20], Ito-22 [19], and GOBAI-O_2 [21, 22]. Intercepts are not shown.

**Period**	**Data**	**Estimate ± se**[b]	**p-value**	$\mathbf{R^2_{adj.}}$	**F-statistic (df**[c]**)**
1967-1994	HIND	-86.49 ± 4.02	.000	.947	463.1 (1, 25)
	Obs.-based: Ito-17	-8.84 ± 22.26	.695	-0.033	0.1577 (1, 25)
	Obs.-based: Ito-22	-31.86 ± 15.60	.052	.108	4.153 (1, 25)
2004-2015	HIND	-28.7 ± 6.30	.001	.642	20.71 (1, 10)
	Obs.-based: GOBAI-O_2	-405.6 ± 33.9	.000	.928	143.3 (1, 10)
	Obs.-based: Ito-22	-133.28 ± 13.88	.000	.892	92.16 (1, 10)

[a] Note that the varying length of the different time periods, which reflect major shifts in the global ocean oxygen trend, affect the uncertainties in the reported oxygen trends, as shorter periods are more susceptible to noise and outliers at the beginning or end of the period.
[b] standard error
[c] degrees of freedom

Table S5: Detailed results of the ordinary least squares regression analyses for the change in globally integrated O_2 reported in Table S1. Estimates refer to fitted slopes (Tmol O_2 dec^{-1}). Intercepts are not shown.

Period	Data	Estimate ± se[a]	p-value	$R^2_{adj.}$	F-statistic (df[b])
1967-1994	HIND	-46.41 ± 4.95	.000	.770	87.86 (1,25)
	WIND	68.99 ± 4.253	.000	.910	262.9 (1,25)
	HEAT-FW	-96.18 ± 4.57	.000	.945	443.6 (1,25)
2002-2018	HIND	-116.75 ± 6.58	.000	.957	315.00 (1,13)
	WIND	25.45 ± 6.10	.001	.540	17.41 (1,13)
	HEAT-FW	-179.50 ± 5.70	.000	.986	991.5 (1,13)
1967-2018	HIND	-35.04 ± 2.84	.000	.748	152.6 (1,50)
	WIND	64.11 ± 1.93	.000	.956	1099. (1,50)
	HEAT-FW	-93.55 ± 2.71	.000	.959	1193. (1,50)

[a] standard error
[b] degrees of freedom

Table S6: Detailed results of the ordinary least squares regression analyses for the change in globally integrated O_2^{sat} reported in Table S1. Estimates refer to fitted slopes (Tmol O_2 dec^{-1}). Intercepts are not shown.

Period	Data	Estimate ± se[a]	p-value	$R^2_{adj.}$	F-statistic (df[b])
1967-1994	HIND	-1.89 ± 2.77	.503	-0.021	0.463 (1,25)
	WIND	36.86 ± 1.86	.000	.938	391.0 (1,25)
	HEAT-FW	-28.46 ± 2.24	.000	.860	160.9 (1,25)
2002-2018	HIND	-69.75 ± 2.72	.000	.979	656.2 (1,13)
	WIND	6.86 ± 2.54	.018	.310	7.293 (1,13)
	HEAT-FW	-76.66 ± 3.79	.000	.967	408.6 (1,13)
1967-2018	HIND	-11.96 ± 2.19	.000	.362	29.95 (1,50)
	WIND	27.39 ± 1.35	.000	.889	409.7 (1,50)
	HEAT-FW	-29.86 ± 1.43	.000	.895	433.6 (1,50)

[a] standard error
[b] degrees of freedom

References

1. Garcia, H. E. *et al.* World Ocean Atlas 2024, Volume 3: Dissolved oxygen, apparent oxygen utilization, and oxygen saturation. *NOAA Atlas NESDIS* **91** (ed Mishonov, A.) (2024).

2. Holte, J., Talley, L. D., Gilson, J. & Roemmich, D. An Argo mixed layer climatology and database. *Geophysical Research Letters* **44,** 5618–5626 (2017).

3. Holte, J. & Talley, L. D. A new algorithm for finding mixed layer depths with applications to Argo data and subantarctic mode water formation. *Journal of Atmospheric and Oceanic Technology* **26,** 1920–1939 (2009).

4. Moat, B. I. *et al. Atlantic meridional overturning circulation observed by the RAPID-MOCHA-WBTS (RAPID-Meridional Overturning Circulation and Heatflux Array-Western Boundary Time Series) array at 26N from 2004 to 2022 (v2022.1) [dataset]* 10.5285/04c79ece-3186-349a-e063-6c86abc0158c (British Oceanographic Data Centre - Natural Environment Research Council, UK, 2023).

5. Melin, F. *GMIS - MODIS-AQUA Monthly climatology sea surface Chlorophyll-a concentration (9km) in mg.m^{-3} [dataset]* http://data.europa.eu/89h/51b9459f-aa6c-4160-9754-3e203c9c99b8 (European Commission, Joint Research Centre (JRC), 2013).

6. Sathyendranath, S. *et al.* Carbon-to-chlorophyll ratio and growth rate of phytoplankton in the sea. *Marine Ecology Progress Series* **383,** 73–84 (2009).

7. O'Brien, T. D. & Moriarty, R. *Global distributions of mesozooplankton abundance and biomass - Gridded data product (NetCDF) - Contribution to the MAREDAT World Ocean Atlas of Plankton Functional Types [dataset]* https://doi.org/10.1594/PANGAEA.785501 (PANGAEA, 2012).

8. Bianchi, D., Galbraith, E. D., Carozza, D. A., Mislan, K. A. S. & Stock, C. A. Intensification of open-ocean oxygen depletion by vertically migrating animals. *Nature Geoscience* **6,** 545–548 (2013).

9. Bianchi, D. & Mislan, K. A. S. Global patterns of diel vertical migration times and velocities from acoustic data. *Limnology and Oceanography* **61,** 353–364 (2015).

10. Chien, C. *et al.* FOCI-MOPS v1 - integration of marine biogeochemistry within the Flexible Ocean and Climate Infrastructure version 1 (FOCI 1) earth system model. *Geoscientific Model Development* **15,** 5987–6024 (2022).

11. Farneti, R. *et al.* An assessment of Antarctic Circumpolar Current and Southern Ocean meridional overturning circulation during 1958–2007 in a suite of interannual CORE-II simulations. *Ocean Modelling* **93,** 84–120 (2015).

12. Moreno-Chamarro, E. *et al.* Impact of increased resolution on long-standing biases in HighResMIP-PRIMAVERA climate models. *Geoscientific Model Development* **15,** 269–289 (2022).

13. Ilıcak, M. *et al.* An assessment of the Arctic Ocean in a suite of interannual CORE-II simulations. Part III: Hydrography and fluxes. *Ocean Modelling* **100,** 141–161 (2016).

14. Rosenblum, E. *et al.* Surface salinity under transitioning ice cover in the Canada Basin: climate model biases linked to vertical distribution of fresh water. *Geophysical Research Letters* **48** (2021).

15. Bao, Y. & Li, Y. Simulations of dissolved oxygen concentration in CMIP5 Earth system models. *Acta Oceanologica Sinica* **35,** 28–37 (2016).

16. Rayner, D. *et al.* Monitoring the Atlantic meridional overturning circulation. *Deep Sea Research Part II: Topical Studies in Oceanography* **58,** 1744–1753 (2011).

17. Patara, L. & Hollitzer, H. A. L. *Model Output used in Hollitzer et al. (2024): Competing effects of wind and buoyancy forcing on ocean oxygen trends in recent decades [dataset]* hdl:20.500.12085/a4d451d5-a68f-401b-b58d-68792a5a0820 (GEOMAR Helmholtz Centre for Ocean Research Kiel [distributor], 2024).

18. Good, S. A., Martin, M. J. & Rayner, N. A. EN4: Quality controlled ocean temperature and salinity profiles and monthly objective analyses with uncertainty estimates. *Journal of Geophysical Research: Oceans* **118,** 6704–6716 (2013).

19. Ito, T. Optimal interpolation of global dissolved oxygen: 1965–2015. *Geoscience Data Journal* **9,** 167–176 (2022).

20. Ito, T., Minobe, S., Long, M. C. & Deutsch, C. Upper ocean O_2 trends: 1958-2015. *Geophysical Research Letters* **44,** 4214–4223 (2017).

21. Sharp, J. D. *et al.* *GOBAI-O_2: A Global Gridded Monthly Dataset of Ocean Interior Dissolved Oxygen Concentrations Based on Shipboard and Autonomous Observations [dataset]* NCEI Accession 0259304, accessed 06. April 2023 (NOAA National Centers for Environmental Information, 2022).

22. Sharp, J. D. *et al.* GOBAI-O_2: temporally and spatially resolved fields of ocean interior dissolved oxygen over nearly two decades. *Earth System Science Data* **15,** 4481–4518 (2023).
