# Supplementary material for: Competing effects of wind and buoyancy forcing on ocean oxygen trends in recent decades
Source: Nat Commun. 2024 Oct 26;15:9264. doi: 10.1038/s41467-024-53557-y (PMC11513149; doi:10.1038/s41467-024-53557-y)
Supplement: Supplementary file 2 — Peer Review File [file 41467_2024_53557_MOESM2_ESM.pdf]

nature portfolio

Peer Review File

Competing effects of wind and buoyancy forcing on ocean oxygen trends in recent decades

Corresponding Author: Ms Helene Hollitzer

This file contains all reviewer reports in order by version, followed by all author rebuttals in order by version.

Version 0:

Reviewer comments:

Reviewer #1

(Remarks to the Author)

Reviewer #2

(Remarks to the Author)
This study used a global eddy-permitting ocean biogeochemistry model to understand the causes of ocean deoxygenation in the last several decades through comparison of model simulation and gridded observation-based data products as well as performing sensitivity experiments. This is a well written paper and uses a state-of-the-art modeling tool validated with new observational products. The authors discussed the results in logical order. First, compensating role of wind and buoyancy forcing are evaluated at the global scale. Then, somewhat more detailed analyses at the regional scales are provided. Implications are discussed at the end. Adequate model descriptions are provided in the method section as well as additional model validations in the supplemental document.

Starting from the conclusion, I support the publication of this manuscript with a minor revision. My view is consistent with that of the authors that there are likely multiple causes for the mismatch between ocean hindcasts and observation-based O2 products. But the authors can link their results with another recent study, and make a stronger statement about the problem of current ocean hindcast simulations, including its model spin-up process.

It is somewhat known in the literature that hindcast models seem to underestimate the recent deoxygenation rates found in observational datasets. I support the authors' assessment that this is a common problem across many ocean hindcast simulations. I encourage the authors to consider comparing their results with Takano et al (2023, https://www.frontiersin.org/articles/10.3389/fmars.2023.1139917/full) as they focused on similar issues using OMIP1/2 models.

One of the causes in poor performance of hindcast models may be in the spin-up approach. The repeat application of O(60 year from late 20th to early 21st century) atmospheric reanalysis data causes significant accumulation of excess heat in the ocean interior. When the last cycle is used for analysis, the ocean model was initialized with unrealistic OHC, leading to weak rises in OHC together with weak deoxygenation rates. Indeed, this study also used multiple cycles of reanalysis forcing (according to method section) and this spin-up approach may have affected the resulting representation of deoxygenation trends.

Overall, this is a high-quality paper in terms of the concepts and writing, and only minor revision would be necessary in my opinion.

Reviewer #3

(Remarks to the Author)
Review of the manuscript NCOMMS-24-15163: "Competing effects of wind and buoyancy forcing on recent ocean oxygen trends" by Hollitzer et al.

Review invitation: 15-3-2024
Review accepted: 18-3-2024
Review sent: 24-3-2024

General comment:

Detecting and understanding the drivers of ocean properties changes like oxygen (O2), carbon dioxide (DIC), temperature or salinity is challenging given the climate change and associated multiple forcing (wind, circulation, warming, including volcanoes, e.g. Fay et al, 2023). For O2, the deoxygenation could lead to dramatic impacts on marine ecosystem (Breitburg et al, 2018). Thanks to the synthesis of observations, the multi-decadal change of O2 in the ocean is now estimated at global scale (e.g. Schmidtko et al, 2017; Ito et al, 2017; Roach and Bindoff, 2023; Sharp et al, 2023), although there are regions where the data are still sparse and the long-term trend uncertain, such as in the southern ocean and in deep layers (Ito et al, 2024).

How this will change in the future is a topic that can be addressed using Earth System Models (ESM, e.g. Kwiatkowski et al, 2020; Ditkovsky et al, 2023 for the Indian Ocean). Most results show that O2 will continue to decrease in the future but this is still uncertain depending the scenarios and models. What will be the main drivers of the future O2 change need to be evaluated (warming, wind, stratification, production/export, etc…). This is challenging as for the recent decades this could not be estimated directly from observations (excepted the effect of warming, e.g. Roach and Bindoff, 2023) and the internal processes or external forcing that drive O2 change in surface and at depth remain unclear. Quoting previous studies (Oschlies et al 2018): "Current models may underestimate deoxygenation because of unresolved transport processes, unaccounted for variations in respiratory oxygen demand, or missing biogeochemical feedbacks"

To investigate the drivers, here over 1958-2018 and including inter-annual variability, authors used a hindcast model (noted HM here) and compare 3 simulations in order to separate the effects of wind and heat/freshwater fluxes: (i) a full reanalysis forcing (HIND) corrected to drift, or applying part of the forcing, namely (ii) WIND or (iii) HEAT-FW simulations. The final aim, as indicated in the introduction, is to improve the prediction (and understanding) of O2 changes in the future. In this context one would expect to see what are the changes of the O2 concentrations and total O2 inventory in the oceans, not only the anomalies as presented in figures 1, 2 or 4.

Although I found attractive parts of the discussion (the comparison with ESM, figure 4), I have to admit that I am not convinced with all results (drivers analysis). The paper needs clarification, especially concerning the validation of the HM. At the end, my feeling is that, through the question addressed (focused on O2 changes in the ocean), the analysis offers interesting information concerning the origin of the bias in the HMs (discussed in the conclusion) but results on the drivers of the observed O2 change are not clear (the title of the manuscript). Authors conclude: "we stress the importance of the accurate representation of wind stress and air-sea heat and freshwater fluxes in reanalysis data for assessing past deoxygenation with GOBMs, which do not capture trends in O2 and OHC since 2002." Would it be better to write: "we stress the importance of the accurate representation of wind stress and air-sea heat and freshwater fluxes in reanalysis data before assessing past deoxygenation with GOBMs". In other words, if you know the forcing are not accurate, are the results for the drivers correct ?

For these reasons I do not recommend publication in its present form. Below are listed other specific comments and questions.

;;;;;;;;;; Specific comment and questions

C-01: Title: "Competing effects of wind and buoyancy forcing on recent ocean oxygen trends".
Word "Recent" suggest you are investigating the recent years (i.e. after 2015 or so when the observations show a dramatic decrease). Maybe change the title: "Competing effects of wind and buoyancy forcing on ocean oxygen trends".

C-02: Line 88: Section "Trends in the global ocean oxygen inventory". Authors describe the change of inventory following 4 periods (based on observed trends). Figure 1 and 2 suggests that the HM underestimates the inventory change on average. It is also shown that the HM model is not able to reproduce decadal variations, e.g. the peak around 1985 or the rapid change after 2002. Around 1985 this is probably linked to large change in the SO (Roach and Bindoff 2023); do you see that signal in the model ? After 2002, the HM is clearly stagnant (as noted by authors, lines 104-105). Given these differences how can we trust the driver analysis derived from the model (the 3 simulations described in section 2.2.1) ?

C-03: Line 134: "The acceleration of deoxygenation over the last two decades (Fig. 1) has been caused simultaneously by a smaller increase in wind stress and associated wind-driven O2 increases, and an increase in O2 losses driven by changing air-sea heat and freshwater fluxes (Fig. 1a)." I don't clearly understand this point. The model shows the acceleration for the full water column, not in the 0-1000m layer (figure 1b). Those processes impact only the deep ocean ? Please clarify.

C-04: Line 150: "The equatorial regions show an overall deoxygenation that is strongest in the 100-400 m range, consistent with the observed expansion of the tropical OMZs". Note that trends presented in figure S7 for the Indian ocean also show an increase of O2 in equatorial region; that seems coherent with those simulated in the future, i.e. an increase of O2 in the tropics around 100-1000m (Ditkovsky et al,2023).

C-05: Line 158: "During the strong El Nino conditions of 1997-98 [47], global oxygen levels fell, as simulated by the model

and shown by observations (Fig. 1).” Is the ENSO signal from the observations and model really significant ? Why this is not observed for other ENSO events, in 1982-1983 or 2015 ?

C-06: Line 186: “Indeed, we find that the insufficient deoxygenation (<700 m) in the model analysed here (ORCA025-MOPS) is also simulated by global ocean biogeochemistry models (GOBMs) participating in RECCAP2 [56] (Fig. 4a), which largely contribute to the Global Ocean Carbon Budget”. Reference 56: I think De Vries et al did not discuss the O2 budget. It might be relevant to plot the carbon flux from RECCAP2 (like the O2 and OHC shown in Figure 4). At global scale there is an increase of the carbon sink after 2000 from the data-products (e.g. De Vries et al 2023), a signal not clearly simulated by GOBMs (see also Terhaar et al, 2024). This occurred about the same period (around year 2000) where you identified a shift between ESM and GOBMs (your Figure 4). Would that be linked to the forcing fields used in GOBM as discussed in your conclusion ?

C-07: Line 209: “By contrast, coupled Earth system models (ESMs) from CMIP6, which often have the same ocean components as those used in GOBMs, simulate upper 700 m deoxygenation and ocean warming trends that are consistent with observation-based estimates within uncertainties, but at the lower end (Fig. 4)”. Given this result, would it be better to use ESM outputs for the driver analysis ?

C-08: Line 232: “The underestimation of the strong decline in oxygen and rise in OHC over the last 18 years by GOBMs raises the question of whether the GOBM-derived trends in the ocean carbon sink, as reported by the Global Carbon Budget [57], are similarly biased by systematic biases due to the atmospheric forcing or the hindcast approach in general.” After 2000 GOBMs show that the CO2 sink is low compared to the data-products (see comment C-06). This was identified in previous GCB reports as well (Friedlingstein et al, 2020, 2022). If this is due to atmospheric forcing, then I guess the forcing is not suitable to separate the drivers as discussed in your analysis.

C-09: Line 239: “In our simulations, a key region for global oxygen trends is the Southern Ocean..”.
This is also the region where long term observations are sparse to evaluate the trend accurately.

C-10: Line 241: “In this region, the recent strengthening of westerly winds, caused by increasing greenhouse gas emissions and stratospheric ozone depletion [31], has counteracted the deoxygenation caused by widespread surface warming and mid-depth freshening [64, 65].” ESM that include both ozone depletion and GHG forcing suggest different response of the carbon fluxes (e.g. Lenton et al 2009). It might be relevant later to test this for O2. Concerning the freshening, I think there are still large uncertainties in the E/P forcing. Did you test other forcing (e.g. ERA54).

C-11: Line 256: “Our study indicates that the stabilisation or reversal of the past intensification of wind stress, together with continued ocean warming, is likely to accelerate oxygen loss in the future, particularly in the Southern Hemisphere intermediate waters”. As the HM is not really reproducing the recent acceleration of O2 trends (figure 1b), can you really conclude that your results suggest the O2 decrease will be accelerate in the future due to change in wind stress ?

C-12: Line 262: “Given the centrality of atmospheric drivers in determining trends in oceanic oxygen, we stress the importance of the accurate representation of wind stress and air-sea heat and freshwater fluxes in reanalysis data for assessing past deoxygenation with GOBMs, which do not capture trends in O2 and OHC since 2002.” I agree with this suggestion; that seems the main conclusion of the analysis, i.e. the HM model is not able, at present, to reproduce observed O2 changes.

C-13: Line 288: What is Cnat ? (natural carbon, i.e. not anthropogenic)

C-14: Line 355: “Additionally, in Supplementary Text 1 and Supplementary Figs. 11-13, we compare observational climatological distributions of dissolved oxygen and Osat2 against the corresponding model outputs.” I appreciate the comparison of the model with observations. This shows large differences up to -50 or +50 mmol/m3 at regional scale. For example, in the Southern Ocean, differences reach +25 mmol/m3 at depth (1000m, figure S11f). Does that would import more O2 in the top layers in the model than in reality (i.e. a positive trend of O2 over time) ? Also, here authors compared the climatology from 1958-2018 but as there is a decrease in O2, would it be better to compare the model for a shorter period (1980-2000 or 1980-1994) and when WOA data are available (few data before 1970).

C-15: Figure 1 and others: not easy to distinguish the color of lines (also figures S3 and S6). Maybe use dashed for observations ?

C-16: Figure 1: could you add error range for the Observations (like in figure 2a for Ito-17)?

C-17: Figure 2a nicely shows the trend from Ito-17. Could you add also Ito-17 in figures S1a and S2a ?

C-18: Figure S7: Is it useful to show the trend over 1967-2018 as the trends are different depending the periods. Maybe show the same but for 2 periods.

C-19: Figure S7: is it possible to compose the same figure S7 with observations (suggestion) ?

C-20: Figure S7: why for the Indian Ocean the results are presented north of 30°N ?

C-21: It would be interesting to include maps of O2 trend in surface (0-100m) and at 500m and compare with observations.

Same could be prepared for O2 inventory trend (in 0-1000m, see Ito et al, 2024, their figure 3).

;;;;;;;;; References in this review not listed in the MS:

Ditkovsky, S., Resplandy, L., and Busecke, J.: Unique ocean circulation pathways reshape the Indian Ocean oxygen minimum zone with warming, Biogeosciences, 20, 4711–4736, https://doi.org/10.5194/bg-20-4711-2023, 2023.

Fay, A. R., McKinley, G. A., Lovenduski, N. S., Eddebbar, Y., Levy, M. N., Long, M. C., Olivarez, H. C., and Rustagi, R. R.: Immediate and Long-Lasting Impacts of the Mt. Pinatubo Eruption on Ocean Oxygen and Carbon Inventories, Global Biogeochem. Cy., 37, e2022GB007513, https://doi.org/10.1029/2022GB007513, 2023.

Friedlingstein, P., et al: Global Carbon Budget 2020, Earth Syst. Sci. Data, 12, 3269–3340, https://doi.org/10.5194/essd-12-3269-2020, 2020.

Friedlingstein, P., et al: Global Carbon Budget 2022, Earth Syst. Sci. Data, 14, 4811–4900, https://doi.org/10.5194/essd-14-4811-2022, 2022.

Ito, T., Garcia, H. E., Wang, Z., Minobe, S., Long, M. C., Cebrian, J., Reagan, J., Boyer, T., Paver, C., Bouchard, C., Takano, Y., Bushinsky, S., Cervania, A., and Deutsch, C. A.: Underestimation of multi-decadal global O2 loss due to an optimal interpolation method, Biogeosciences, 21, 747–759, https://doi.org/10.5194/bg-21-747-2024, 2024.

Lenton, A., et al, 2009. Stratospheric ozone depletion reduces ocean carbon uptake and enhances ocean acidification. Geophys. Res. Let.,36, L126061-5. https://doi.org/10.1029/2009GL038227

Roach, C. J., and N. L. Bindoff, 2023: Developing a New Oxygen Atlas of the World's Oceans Using Data Interpolating Variational Analysis. J. Atmos. Oceanic Technol., 40, 1475–1491, https://doi.org/10.1175/JTECH-D-23-0007.1.

Terhaar, J., Goris, N., Müller, J. D., DeVries, T., Gruber, N., Hauck, J., et al. (2024). Assessment of global ocean biogeochemistry models for ocean carbon sink estimates in RECCAP2 and recommendations for future studies. Journal of Advances in Modeling Earth Systems, 16, e2023MS003840. https://doi.org/10.1029/2023MS003840

;;;;;; end review

Reviewer #4

(Remarks to the Author)
I co-reviewed this manuscript with one of the reviewers who provided the listed reports. This is part of the Nature Communications initiative to facilitate training in peer review and to provide appropriate recognition for Early Career Researchers who co-review manuscripts.

Version 1:

Reviewer comments:

Reviewer #1

(Remarks to the Author)
The authors has addressed all my questions. I agree the paper to publish.

Reviewer #2

(Remarks to the Author)
This is the second review of Hollitzer et al. "Competing effects of wind and buoyancy forcing on ocean oxygen trends in recent decades". I appreciate the effort of the authors to address the previous review comments. The authors addressed all of the comments, not only mine but also for the other reviewers with in depth responses. I have no further comments at this time. I support the publication of this revised manuscript.

Reviewer #3

(Remarks to the Author)
General comment:

I am very satisfied with author's responses and the revised manuscript, including revised figures in the main text and in the Supplementary Material. Their responses to all reviewers are very well prepared.

For comparing the model with observations, authors now used the Ito-22 data-set and thus enabled to compare the full water column. In addition they mentioned significant differences between the 3 data-sets, an interesting result that opens questions

regarding the origin of these differences, a challenge for new analysis dedicated for a better evaluation of O2 changes in the ocean and thus to validate model results GBOM or ESM.

In the revision, authors described and interpret in more detail the anomalies at regional scale (e.g., North Atlantic and convection, Southern Ocean and wind stress strengthening) that would also help to interpret the changes in other properties (e.g. anthropogenic CO2 inventories).

The manuscript offers several message for the modeling community (adapt the spin-up protocol, test different forcing fields) and for the observational community (more observations especially in the Southern Ocean, add deep Argo-O2 floats in the future).

The paper is suitable for publication in its present form. See very few comments below that could be taken into account for the final version.

Specific comments:

C-01: Line 188-193: Authors write: “The highest levels of O2 short-term variability are found, for example, in strongly dynamic regions of water mass formation, frontal dynamics, and ocean-sea ice interaction (Supplementary Fig. 8). Instead, there is little variability in the centre of the subtropical gyres and in the Weddell and Ross gyres, and within the OMZs due to their inherently low oxygen concentrations”. Not sure to see that in Figure S8. The model does not show high variability in ocean-ice region but this apparently captured from observations. Also, the observations and the model both show high variability in the eastern boundaries (OMZ sector?).

C-02: Line 204-206: Authors write: “By comparing these regional trends with observation-based estimates, we find that while global deoxygenation is generally underestimated, regional trends can be captured with greater accuracy, e.g. in the North Atlantic and equatorial Pacific.” You may also notice that in the North Atlantic there is a very good comparison with GOBAI-O2 after 2010 as shown in Figure 2c (but this is somehow written on lines 223-224).

C-03: Line 259: Authors write: “Large disagreements exist also between the trends estimated by observation-based datasets in the Southern Ocean (Fig. 2e).” This is also an interesting result in this manuscript; any idea why the datasets present so large differences ? Curiosity: Is the result would be the same when changing the limit of the Southern Ocean taken at 40°S, 45°S or 50°S (here authors used 30°S).

C-04: Line 264: Authors write: “As observation-based estimates of the ocean carbon sink have also been shown to overestimate the variability in the Southern Ocean [68] as well as the trends [69], we cannot conclude with certainty if models or observation-based estimates are closer to reality.
.” What do you mean by “reality”? Surface trends or inventories ? I agree with authors, but I guess this sentence somehow mixed different topics. This depends on the property and “reality” one is looking for. For example the view of the changes (and “real changes”) of air-sea CO2 fluxes and Cant inventories in the Southern Ocean also depends on the boundary selected (see comment C-03) and selected period.

C-05: Figure S6: I guess iso-sigma plotted are the same for each basin. Maybe just indicate this in the caption (no need to revise the figure).

;;;;;;;;; end review

Reviewer #4

(Remarks to the Author)
The revised version is improved and the newly added Figure 2 and Supplementary Figures and Tables for regional oxygen change provide additional information that are helpful and of interest to the readers. The revised manuscript explains in detail the time periods and regions of discrepancy between observations and model simulations, thereby increasing the reliability of the conclusions. Therefore, I recommend its acceptance for publication.

I co-reviewed this manuscript with one of the reviewers who provided the listed reports. This is part of the Nature Communications initiative to facilitate training in peer review and to provide appropriate recognition for Early Career Researchers who co-review manuscripts.

Open Access This Peer Review File is licensed under a Creative Commons Attribution 4.0 International License, which permits use, sharing, adaptation, distribution and reproduction in any medium or format, as long as you give appropriate credit to the original author(s) and the source, provide a link to the Creative Commons license, and indicate if changes were made.
In cases where reviewers are anonymous, credit should be given to 'Anonymous Referee' and the source.
The images or other third party material in this Peer Review File are included in the article's Creative Commons license, unless indicated otherwise in a credit line to the material. If material is not included in the article's Creative Commons license and your intended use is not permitted by statutory regulation or exceeds the permitted use, you will need to obtain permission directly from the copyright holder.
To view a copy of this license, visit https://creativecommons.org/licenses/by/4.0/

Dear Reviewers,

We are very grateful for the time and effort you have put into reviewing our manuscript and thank you for your valuable feedback, which we believe has substantially improved the manuscript. We have carefully considered all the comments and have done our best to address each of them.

To address your comments, we have made several key revisions:

1. We have extended our model evaluation to include the observation-based ocean oxygen dataset developed by T. Ito (2022), highlighting the uncertainty present in observation-based products.
2. We have improved our discussion on the origin of the insufficient deoxygenation in the model, attributing it to the spin-up procedure rather than to the atmospheric forcing, and added new figures showing biases in the mean temperature and temperature trend to support our interpretation. Additionally, owing to the flawed spin-up procedure, we have excluded the years 1958 to 1967 from all four experiments in our analyses. This exclusion addresses the initial shock and associated recovery phase that the model undergoes when the forcing abruptly jumps from 2018 back to 1958.
3. We calculated the solubility-driven and non-solubility-driven components of O_2 changes from an observation-based ocean reanalysis and included these calculations in Figs. 3, S3, and S5 to provide a better understanding of how the spin-up and ocean heat content trend bias affect the different components of O_2 changes.
4. We have added new figures showing (1) oxygen changes (modelled and observation-based) in a number of sub-regions and (2) global maps of the standard deviation of the O_2 trend compared to observations, demonstrating the ability of the model to capture the variability of oxygen across different regions.

A detailed point-by-point response to the comments of reviewers 1 to 3 (following the reviewers' order in the decision letter) is given below. All line numbers and figure references in our responses refer to the revised manuscript file, unless stated otherwise. Additionally, quotations from the original or revised manuscript are in italics, and line numbers and table and figure references are in bold.

A list of the new and revised figures and how they differ from the first submission is provided at the end of this document.

We sincerely appreciate your thoughtful review and hope that the revisions we have made have improved the quality and clarity of our paper.

Yours sincerely and on behalf of all co-authors,

Helene Hollitzer

Ito, T. (2022), Optimal interpolation of global dissolved oxygen: 1965–2015, *Geoscience Data Journal*, **9**, 167–176, doi:10.1002/gdj3.130.

1 Response to reviewer 1

General Comments

The authors have done a great deal of work. I have one major concern. Figure 1 shows a notable discrepancy between the observational data and the modeled outputs. This discrepancy could significantly impact the study's outcomes. The authors have acknowledged this issue in their discussion and have devoted considerable effort to analyzing the reasons for the oxygen underestimation of the models. However, considering that the study's conclusions are heavily based on these models, it's crucial to understand the extent to which this model-data mismatch affects the research's overall findings and interpretations. I recommend that the authors provide a more detailed analysis of the implications of these discrepancies.
The flow in the results part could be improved. Currently, it is hard to follow.

Response: We thank the reviewer for the insightful comments. We have addressed their concern with a more in depth analysis of the causes and implications of the O_2 trend bias.

The steps taken to address the reviewer's comments (detailed below) are summarised here:

1. We now refer to recent studies attributing the insufficient ocean heat content (OHC) rise and deoxygenation in global ocean models to the spin-up procedure rather than to the atmospheric forcing.
2. We calculated the solubility-driven and non-solubility-driven components of O_2 changes from an observation-based ocean reanalysis to better understand how the OHC trend bias affects the different components of O_2 changes and in which time periods.
3. We extended the model evaluation to include the ocean oxygen dataset developed by T. Ito (2022). This additional analysis highlights the uncertainty present in observation-based products after 2000.
4. We extended the analysis of the regional O_2 variability and highlight the skill of the model in this respect.

Below, we provide you with more detailed information on the above points:

1. A recent study by Takano et al. (2023) concluded that the misrepresentation of present-day deoxygenation, which is a common deficiency in state-of-the-art ocean models, can be attributed to the procedure which most GOBMs (including this study) use to spin-up the model, which involves the cycling over several cycles of present-day forcing (Tsujino et al., 2020). This leads to an overestimated value of the ocean heat content (OHC) at the start of the hindcast period and makes the models less sensitive to global warming, leading to an underestimated OHC rise (Huguenin et al., 2022) and deoxygenation (Takano et al., 2023). These past studies are wholly consistent with the underestimation of OHC rise and of deoxygenation that we find in this and other GOBMs (Fig. **4**). To support this interpretation, we added an additional figure in the Supplementary Materials (Fig. **S2**), which shows biases in the mean temperature and temperature trend. The main text has been modified on lines **70-82**, **143-147**, and **295-307**.

We additionally assess that until the early 2000s the model was close to the observation-based estimates, whereas the largest discrepancies arise after 2000 (Fig. **2**), corresponding to an acceleration of the OHC rise, which the model is not able to reproduce (Fig. **4**). This finding is supported by two

new figures in the Supplementary Materials (Figs. **S3**, **S5**), discriminating between the two periods. We also discuss that the HEAT-FW experiment is likely more biased than the WIND experiment which, by construction, is not meant to capture the direct effects of global warming (but only its indirect effects manifested through wind stress changes). We discuss these aspects on lines **308-311**.

2. To assess whether the biases in the O_2 trends are predominantly driven by its solubility component or its non-solubility component, we computed the solubility-driven and non-solubility-driven O_2 components from the observation-based ocean reanalysis EN4.2.2 (Good et al., 2013). We found that the biases in the O_2 trend after 2000 are primarily due to misrepresented O_2^{sat} rather than the non-solubility-driven component. Again, we find that the bias is particularly pronounced after 2000, while it remains relatively small for the period 1967 to 1994. We have included these calculations in Figs. **3**, **S3**, and **S5**. The main text has been changed on lines **151-161** and **284-288** of the revised manuscript.

3. We included an additional observation-based dataset developed by T. Ito (2022), Ito-22, which differs from Ito et al. (2017) in terms of the underlying data and gap-filling methodology. We find that the two Ito products diverge significantly after 2000, highlighting the uncertainties associated with observation-based estimates used to evaluate ocean models. We also show that Ito-22 shows suspiciously strong deoxygenation in the Southern Ocean below 2000 m (Fig. **S6**), where sampling is known to be particularly sparse. We have included the new Ito-22 data in Figs. **2, 3, 4, S3, S5, S6,** and **S12**. We have modified the main text on lines **132-142, 155-166, 284-294**, and **440** of the revised manuscript.

4. We extended the analysis of regional patterns of change. We have computed spatial maps of O_2 variance in the model and in Ito-17, and find good agreement between the two data classes (Fig. **S8**). We now also show the temporal evolution of O_2 (as well as its solubility and non-solubility-driven components in Fig. **S13**) in four key regions for O_2 dynamics (Fig. **2**): the North Atlantic, the North Pacific, the Equatorial Pacific, and the Southern Ocean. We find good agreement between the model and observation-based estimates, particularly in the North Atlantic and Equatorial Pacific. We show that the regional O_2 variability is more influenced by non-solubility-driven changes than by solubility-driven changes (Fig. **S13**), implying that the biased O_2^{sat} after 2000 should not strongly influence the regional variability. The additional analysis also highlights the importance of the Southern Ocean in setting trends on a global scale. We have changed the main text on lines **186-275** (Section: Regional estimates), **288-289**, and **311-319** of the revised manuscript.

Finally, we worked on improving the flow of the results section.

Comment 1.1

Line 2: Climate change influences oceanic oxygen levels through a variety of processes beyond those mentioned. I understand the authors want to lead to their research topic. But this sentence sounds bias.

Response:

We modified the original sentence (lines **2-3** in the original manuscript): *"Climate change affects ocean oxygen by altering wind fields and air-sea heat and freshwater fluxes"*, to (lines **3-4**): *"Two*

major pathways through which climate change affects ocean oxygen are changes in wind fields and changes in air-sea heat and freshwater fluxes".

Comment 1.2

Lines 23-35: This paragraph described the physical and biological processes that influencing distribution of dissolved oxygen in ocean interior. This is of little relevance to this topic of ventilation processes to ocean deoxygenation in this manuscript. I suggest to delete this paragraph.

Response:

We appreciate your concerns about the paragraph's relevance and have now made the paragraph more concise and focused (lines **24-32**). We believe that this provides a valuable foundation for the broad Nature Communications audience to fully understand our analysis of ocean oxygen dynamics, which considers not only ventilation but also the contributions of solubility and respiration.

Comment 1.3

Lines 51-58: It is not necessary to spend a great deal of space demonstrating solubility-related changes have less contribution to ocean deoxygenation.

Response:

We agree and have therefore shortened the paragraph to (lines **46-48**): *"Anthropogenic climate change, manifested amongst other things by ocean warming [22] and changing wind fields [23, 24], has had far-reaching effects on ocean oxygen concentrations in recent decades. Ocean warming directly reduces oxygen solubility and accounts for about 15% of the global oxygen loss [2, 25]. [...]"*.

Comment 1.4

Lines 59-71: This paragraph is very confusing. For example, the first sentence mentioned "anthropogenic warming also intensifies near-surface stratification and modifies wind fields", the next three sentences only described the effects of ventilation on dissolved oxygen without introducing the role of wind. Moreover, the examples of the Pacific Ocean and Southern Ocean cannot explain the effects of ventilation on ocean deoxygenation.

Response:

We have restructured and edited the paragraph to improve clarity (lines **49-60**). The examples of the Pacific and Southern Oceans are intended not only to explain the effects of ventilation on ocean de-oxygenation, but also to introduce the large regional variability in the effects of climate change (particularly with respect to changing wind fields) on ocean oxygen. Although anthropogenic climate change has caused an overall loss of oxygen, there are also mechanisms and regions with the potential of mitigating global deoxygenation.

Comment 1.5

Supplementary Fig. 3: The trends of globally integrated remineralization rate (RR) anomalies for the period of 1967 –1994 need to mark in this figure, and it will be helpful to understand the result of "remineralization rates gradually decrease throughout the simulation period".

Response:

As suggested, we have added the linear fit to Fig. **S1** (Supplementary Fig. **3** in the original submission).

Comment 1.6

Line 89: Can the authors provide some insight into the methodology used to determine the four periods mentioned? The separation years seem arbitrary. In addition, the trends of global ocean oxygen inventory have been separated into four periods, including 1958–1967, 1967–1994, 1994–2002, and 2002–2018. But these four durations are not equal. Is this a meaningful comparison of trends?

Response:

The selection of the four periods was based on visual inspection to capture the main shifts in the global ocean oxygen trend, aiming to identify the underlying mechanisms that may vary across different intervals (and marked by different oxygen trends). When comparing trends between periods, we normalise changes by calculating rates (in Tmol per decade). We do not average or otherwise sum up the findings of the different time periods.

We have included a statement with each table showing the trends in oxygen or oxygen saturation for the different time periods (Tables **S1**, **S3**, and **S4**): *"Note that the varying length of the different time periods, which reflect major shifts in the global ocean oxygen trend, affect the uncertainties in the reported oxygen trends, as shorter periods are more susceptible to noise and outliers at the beginning or end of the period".*

Please also note that we now exclude the years 1958 to 1967 (first period) from all our analyses due to the initial shock and associated recovery phase that the model undergoes when the forcing abruptly jumps from 2018 back to 1958.

Comment 1.7

Figure 1: The colors used to differentiate between various time series are challenging to distinguish.

Response:

We now use more contrasting colours and thicker lines to make it easier to distinguish between the different lines.

Comment 1.8

Lines 113-115: What cause the transition from non-solubility to solubility effects becoming the primary drivers of deoxygenation in the 1990s? Further exploration and clarification are needed.

Response:

This is an important and interesting question, and we agree with the reviewer that it should be better explained. The increasing importance of the solubility effect after the early 2000s coincides with an acceleration of the rise in ocean heat content from around 2000 (see main text Fig. **4**), and these two trends are likely to be related. Table **S1** supports this idea, showing a large increase in oxygen depletion due to changes in heat and freshwater fluxes in the last period (2002-2018), whereas previously changes in solubility tended to be offset by changes in wind stress. This shift towards solubility-driven deoxygenation is consistent with observation-based estimates, which we now highlight in the manuscript (lines **116-121**): *"[...] Afterwards, the model suggests that non-solubility-driven and solubility-driven changes each contribute about half of the global oxygen depletion. This shift from predominantly non-solubility-driven deoxygenation to a roughly equal contribution from solubility is consistent with observation-based estimates (Supplementary Table 3). This change coincides with an acceleration of the rise in global Ocean Heat Content (OHC), although the model underestimates this increase, as discussed in the following section".*

Comment 1.9

Line 120: Is the "decrease" mentioned here significant or just internal variability?

Response:

The decrease in remineralisation rate is significant based on the assumptions underlying the p-value, and the results of the linear regression are now shown in Fig. **S1**. Our main point is not that there is a significant decrease, but rather that there is no increase in remineralisation rates. Our argument holds as long as remineralisation rates remain stable or do not show a significant increase. Please also see our response to comment 1.5.

Comment 1.10

Lines 126-133: The results of global drivers underlying ocean oxygen trends are confusing, especially in the contribution of buoyancy fluxes and wind stress on trend of oxygen.

Response:

We have revised the section to improve its flow and clarity. The updated section can be found on lines **167-185**, with the part on the contribution of buoyancy fluxes and wind stress on lines **177-185**.

Comment 1.11

Line 130: How did the authors conclude that the 65% of oxygen loss is attributed to the reduced ventilation?

Response:

As stated in the methods section, we split oxygen changes into solubility-driven changes and non-solubility-driven changes (i.e. oxygen changes driven by changes in respiration or ventilation). O_2 solubility in seawater was approximated by O_2^{sat}, and non-solubility-driven oxygen changes were calculated by subtracting the solubility component from the total oxygen anomaly (Ito et al., 2017). In Table **S1** we show that in the HEAT-FW experiment oxygen changed at a rate of -94 Tmol per decade and O_2^{sat} at a rate of -30 Tmol per decade (about 32% of the total - updated) during the deoxygenation period, with the remainder due to non-solubility driven changes. As respiration does not contribute to the deoxygenation trend on a global scale (see also our response to comment 1.9), we conclude that the remaining 68% is due to ventilation changes. We have added the (previously missing) reference to Table **S1** to the manuscript (line **172**).

Comment 1.12

Lines 138-139: This sentence is very confusing. What is the increase in oxygen decreased by about 60%?

Response:

We agree that this wording is confusing and have therefore changed the original sentence (lines **138-139** in the original manuscript): *"At the same time, the increase in oxygen due to increased wind stress decreased by about 60% (Supplementary Table 1)"*, to (lines **182-185**): *"At the same time, although there is still some increase in ocean oxygen due to wind stress-driven processes, this increase is only about 40% the magnitude of the wind stress-driven oxygen increase estimated between 1967 and 1994 (Supplementary Table 1)"*.

Comment 1.13

Lines 157-163: In the equatorial Pacific, the declining oxygen content was dominated by non-solubility-driven changes due to wind stress. The non-solubility-driven changes in this manuscript refer to the change in upward transport of low-O_2 waters and intensity of upwelling. This is inconsistent with "the terms" non-solubility-driven changes" and" ventilation-driven changes" are used synonymously hereinafter.", which was mentioned above. In addition, the context is contrary to the results on the effect of upwelling on oxygen trends. In the Introduction, the weak upwelling could mitigate deoxygenation by reducing oxygen-consuming respiration of organic matter due to less nutrient-rich deeper waters and biological production in. In the result of "Regional drivers", the less intense upwelling eventually result in the decline of the oxygen inventory.

Response:

We agree that the generalisation from non-solubility-driven changes to ventilation-driven changes is oversimplified, especially on a regional scale, and therefore no longer use these words as synonyms. We have also added the missing process to the introduction (lines **49-54**).

Comment 1.14

Line 166: Given that the authors mentioned that PDO greatly affect the oxygen in the North Pacific, did the authors compare this effect with the other examined processes such as wind, solubility in the paper?

Response:

In response to the reviewer's comment, we have extended our analysis to include regional time series of oxygen inventory anomalies, including the North Pacific Ocean (Fig. **2**). We also decompose the total oxygen changes in each sub-region into solubility-driven and non-solubility-driven components (Fig. **S13**). In the North Pacific, both solubility and non-solubility effects contribute to the simulated oxygen changes, with buoyancy forcing largely influencing the solubility-driven changes and wind stress largely influencing the non-solubility-driven changes.

Regarding the influence of the Pacific Decadal Oscillation (PDO) on oxygen levels in the North Pacific, we found no clear correlation between our North Pacific oxygen time series and the PDO time series. This is in line with expectations given the complex nature of the PDO pattern and the horseshoe pattern of warm and cold anomalies. Our sub-regional cut-out includes parts of both warm and cold anomalies due to the large area covered (25°N to 60°N; 110°E to 100°W), which is not designed to separate the PDO pattern in the Pacific. Therefore, while the PDO certainly affects oxygen levels in the North Pacific, its effect may be masked by the averaging over a large sub-region and the simultaneous influence of other drivers such as wind stress.

Comment 1.15

Lines 169-172: It is unclear about the depths of water masses at densities between about $\gamma^n =$ 26.6 kg kg m^{-3} and about $\gamma^n = 27.6$ kg kg m^{-3}, which would make it difficult to understand these results.

Response:

We acknowledge that specifying the depths associated with specific water masses enhances clarity. To address this, we have now included the approximate maximum depths of the neutral density layers discussed, thereby facilitating a better understanding of our results. However, we note that describing oxygen changes in relation to specific water masses (via neutral density surfaces) can be more informative than using depth alone. Therefore, we have retained the description via density and supplemented it with depth estimates for completeness (line **212** and line **216**).

Comment 1.16

Lines 184-187: I understand the decadal variability of oxygen is driven by buoyancy fluxes by water mass formation and intensity of meridional overturning circulation (MOC). So, why the global MOC is driven by wind stress on interannual time scales?

Response:

In the revised version of the manuscript, we have removed the analysis of correlations that distinguish between interannual and decadal time scales. We have therefore removed the above sentence from the manuscript.

To nonetheless provide an answer to the above question, we refer to the paper by Polo et al. (2014), which focuses on the Atlantic MOC (AMOC). In their paper, they find that wind forcing dominates the short-term variability of the AMOC through its effect on Ekman currents and coastal upwelling. Buoyancy forcing becomes important on longer time scales (multiannual and decadal) through the delayed oceanic adjustment (via planetary waves or advection) to changing air-sea heat fluxes and convection.

Comment 1.17

Discussions: The authors may need to discuss about the influence of other processes that were not examined in this paper, especially the chemical and biological processes.

Response:

To address the reviewer's comment, we have added a figure comparing modelled and observed estimates of phytoplankton and zooplankton biomass (Fig. **S14**). We have also expanded the section on potential biases due to misrepresentation of biogeochemical processes in the model, which can be found on lines **376-393**.

Good, S. A., M. J. Martin, and N. A. Rayner (2013), EN4: Quality controlled ocean temperature and salinity profiles and monthly objective analyses with uncertainty estimates, *J. Geophys. Res. Oceans*, **118**, 6704–6716, doi:10.1002/2013JC009067.

Huguenin, M. F., R. M. Holmes, and M. H. England (2022), Drivers and distribution of global ocean heat uptake over the last half century, *Nat. Commun.*, **13**, 4921, doi:10.1038/s41467-022-32540-5.

Ito, T., S. Minobe, M. C. Long, and C. Deutsch (2017), Upper ocean O_2 trends: 1958–2015, *Geophys. Res. Lett.*, **44**, 4214–4223, doi:10.1002/2017GL073613.

Ito, T. (2022), Optimal interpolation of global dissolved oxygen: 1965–2015, *Geoscience Data Journal*, **9**, 167–176, doi:10.1002/gdj3.130.

Polo, I., J. Robson, R. Sutton, and M. A. Balmaseda (2014), The Importance of Wind and Buoyancy Forcing for the Boundary Density Variations and the Geostrophic Component of the AMOC at 26°N, *J. Phys. Oceanogr.*, **44**, 2387–2408, doi:10.1175/JPO-D-13-0264.1.

Takano, Y., T. Ilyina, J. Tjiputra, Y. A. Eddebbar, S. Berthet, L. Bopp, E. Buitenhuis, and others (2023), Simulations of ocean deoxygenation in the historical era: insights from forced and coupled models, *Front. Mar. Sci.*, **10**, 1139917, doi:10.3389/fmars.2023.1139917.

Tsujino, H., S. Urakawa, S. M. Griffies, G. Danabasoglu, A. J. Adcroft, A. E. Amaral, T. Arsouze, and others (2020), Evaluation of global ocean-sea-ice model simulations based on the experimental protocols of the Ocean Model Intercomparison Project phase 2 (OMIP-2), *Geoscientific Model Development*, **13**, 3643-3708, doi:10.5194/gmd-13-3643-2020

2 Response to reviewer 2

General Comments

Starting from the conclusion, I support the publication of this manuscript with a minor revision. My view is consistent with that of the authors that there are likely multiple causes for the mismatch between ocean hindcasts and observation-based O_2 products. But the authors can link their results with another recent study, and make a stronger statement about the problem of current ocean hindcast simulations, including its model spin-up process.

It is somewhat known in the literature that hindcast models seem to underestimate the recent deoxygenation rates found in observational datasets. I support the authors' assessment that this is a common problem across many ocean hindcast simulations. I encourage the authors to consider comparing their results with Takano et al (2023, https://www.frontiersin.org/articles/10.3389/fmars.2023.1139917/full) as they focused on similar issues using OMIP1/2 models.

One of the causes in poor performance of hindcast models may be in the spin-up approach. The repeat application of O(60 year from late 20th to early 21st century) atmospheric reanalysis data causes significant accumulation of excess heat in the ocean interior. When the last cycle is used for analysis, the ocean model was initialised with unrealistic OHC, leading to weak rises in OHC together with weak deoxygenation rates. Indeed, this study also used multiple cycles of reanalysis forcing (according to method section) and this spin-up approach may have affected the resulting representation of deoxygenation trends.

Response: We thank the reviewer for their valuable feedback and for suggesting the comparison with Takano et al. (2023), which indeed helped us to better understand the origin of the deoxygenation bias in the model. In addition to referring to the papers by Takano et al. (2023) and Huguenin et al. (2022), we have extended our analysis to support this interpretation and to constrain which parts of our O_2 estimates can be considered more or less robust. Below is a summary of the major changes made to the manuscript:

1. As mentioned by the reviewer, the study by Takano et al. (2023) concluded that the misrepresentation of present-day deoxygenation can be attributed to the procedure which most GOBMs (including this study) use to spin-up the model, which involves the cycling over several cycles of present-day forcing (Tsujino et al., 2020). This leads to an overestimated value of the ocean heat content (OHC) at the start of the hindcast period and makes the models less sensitive to global warming, leading to an underestimated OHC rise (Huguenin et al., 2022) and deoxygenation (Takano et al., 2023). These past studies are wholly consistent with the underestimation of OHC rise and of deoxygenation that we find in this and other GOBMs (Fig. **4**). To support this interpretation, we added an additional figure in the Supplementary Materials (Fig. **S2**), which shows biases in the mean temperature and temperature trend. The main text has been modified on lines **70-82**, **143-147**, and **295-307**.

2. We additionally assess that until the early 2000s the model was close to the observation-based estimates, whereas the largest discrepancies arise after 2000 (Fig. **2**), corresponding to an acceleration of the OHC rise, which the model is not able to reproduce (Fig. **4**). This finding is supported by two new figures in the Supplementary Materials (Figs. **S3**, **S5**), discriminating between the two periods.

3. To assess whether the biases in the O_2 trends are predominantly driven by its solubility component or its non-solubility component, we computed the solubility-driven and non-solubility-driven O_2 components from the observation-based ocean reanalysis EN4.2.2 (Good et al., 2013). We found that the biases in the O_2 trend after 2000 are primarily due to misrepresented O_2^{sat} rather than the non-solubility-driven component. Again, we find that the bias is particularly pronounced after 2000, while it remains relatively small for the period 1967 to 1994. We have included these calculations in Figs. **3**, **S3**, and **S5**. The main text has been changed on lines **151-161** and **284-288** of the revised manuscript.

Good, S. A., M. J. Martin, and N. A. Rayner (2013), EN4: Quality controlled ocean temperature and salinity profiles and monthly objective analyses with uncertainty estimates, *J. Geophys. Res. Oceans*, **118**, 6704–6716, doi:10.1002/2013JC009067.

Huguenin, M. F., R. M. Holmes, and M. H. England (2022), Drivers and distribution of global ocean heat uptake over the last half century, *Nat. Commun.*, **13**, 4921, doi:10.1038/s41467-022-32540-5.

Takano, Y., T. Ilyina, J. Tjiputra, Y. A. Eddebbar, S. Berthet, L. Bopp, E. Buitenhuis, and others (2023), Simulations of ocean deoxygenation in the historical era: insights from forced and coupled models, *Front. Mar. Sci.*, **10**, 1139917, doi:10.3389/fmars.2023.1139917.

Tsujino, H., S. Urakawa, S. M. Griffies, G. Danabasoglu, A. J. Adcroft, A. E. Amaral, T. Arsouze, and others (2020), Evaluation of global ocean-sea-ice model simulations based on the experimental protocols of the Ocean Model Intercomparison Project phase 2 (OMIP-2), *Geoscientific Model Development*, **13**, 3643-3708, doi:10.5194/gmd-13-3643-2020

3 Response to reviewer 3

General Comments Part 01

[...] To investigate the drivers, here over 1958-2018 and including inter-annual variability, authors used a hindcast model (noted HM here) and compare 3 simulations in order to separate the effects of wind and heat/freshwater fluxes: (i) a full reanalysis forcing (HIND) corrected to drift, or applying part of the forcing, namely (ii) WIND or (iii) HEAT-FW simulations. The final aim, as indicated in the introduction, is to improve the prediction (and understanding) of O_2 changes in the future. In this context one would expect to see what are the changes of the O_2 concentrations and total O_2 inventory in the oceans, not only the anomalies as presented in figures 1, 2 or 4.

Response:

We thank the reviewer for their valuable feedback. We have reworded and clarified the description of the aim of our study to set the right expectations. Specifically, we modified the description of the study's aim to (lines **83-84**): *"In this study, we investigate the interannual to decadal variability of O_2 over the period 1967-2018 using a GOBM at 0.25° horizontal resolution, run under historical atmospheric forcing"*. Additionally, we removed the sentence *"By decomposing oceanic O_2 trends according to their drivers and analysing global and regional changes in O_2, we aim at improving the mechanistic understanding of O_2 changes in the ocean and thereby the ability to understand future changes in dissolved oxygen"* on lines **83-86** of the original manuscript.

General Comments Part 02

Although I found attractive parts of the discussion (the comparison with ESM, figure 4), I have to admit that I am not convinced with all results (drivers analysis). The paper needs clarification, especially concerning the validation of the HM. At the end, my feeling is that, through the question addressed (focused on O_2 changes in the ocean), the analysis offers interesting information concerning the origin of the bias in the HMs (discussed in the conclusion) but results on the drivers of the observed O_2 change are not clear (the title of the manuscript). Authors conclude: "we stress the importance of the accurate representation of wind stress and air-sea heat and freshwater fluxes in reanalysis data for assessing past deoxygenation with GOBMs, which do not capture trends in O_2 and OHC since 2002." Would it be better to write: "we stress the importance of the accurate representation of wind stress and air-sea heat and freshwater fluxes in reanalysis data before assessing past deoxygenation with GOBMs". In other words, if you know the forcing are not accurate, are the results for the drivers correct?

Response: We thank the reviewer for their insightful comments. In addition to referring to recent papers attributing the ocean heat content (OHC) and deoxygenation bias to the spin-up procedure used, we performed a more in depth analysis of the causes and implications of the O_2 trend bias. This allowed us to better constrain which parts of the simulated O_2 estimates can be considered more or less robust.

The steps taken to address the reviewer's comments (detailed below) are summarised here:

1. We now refer to recent studies attributing the insufficient OHC rise and deoxygenation in global ocean models to the spin-up procedure rather than to the atmospheric forcing.
2. We calculated the solubility-driven and non-solubility-driven components of O_2 changes from an observation-based ocean reanalysis to better understand how the OHC trend bias affects the different components of O_2 changes and in which time periods.
3. We extended the model evaluation to include the ocean oxygen dataset developed by T. Ito (2022). This additional analysis highlights the uncertainty present in observation-based products after 2000.
4. We extended the analysis of the regional O_2 variability and highlight the skill of the model in this respect.

Below, we provide you with more detailed information on the above points:

1. A recent study by Takano et al. (2023) concluded that the misrepresentation of present-day deoxygenation, which is a common deficiency in state-of-the-art ocean models, can be attributed to the procedure which most GOBMs (including this study) use to spin-up the model, which involves the cycling over several cycles of present-day forcing (Tsujino et al., 2020). This leads to an overestimated value of the ocean heat content (OHC) at the start of the hindcast period and makes the models less sensitive to global warming, leading to an underestimated OHC rise (Huguenin et al., 2022) and deoxygenation (Takano et al., 2023). These past studies are wholly consistent with the underestimation of OHC rise and of deoxygenation that we find in this and other GOBMs (Fig. **4**). To support this interpretation, we added an additional figure in the Supplementary Materials (Fig. **S2**), which shows biases in the mean temperature and temperature trend. The main text has been modified on lines **70-82**, **143-147**, and **295-307**.

We additionally assess that until the early 2000s the model was close to the observation-based estimates, whereas the largest discrepancies arise after 2000 (Fig. **2**), corresponding to an acceleration of the OHC rise, which the model is not able to reproduce (Fig. **4**). This finding is supported by two new figures in the Supplementary Materials (Figs. **S3**, **S5**), discriminating between the two periods. We also discuss that the HEAT-FW experiment is likely more biased than the WIND experiment which, by construction, is not meant to capture the direct effects of global warming (but only its indirect effects manifested through wind stress changes). We discuss these aspects on lines **308-311**.

2. To assess whether the biases in the O_2 trends are predominantly driven by its solubility component or its non-solubility component, we computed the solubility-driven and non-solubility-driven O_2 components from the observation-based ocean reanalysis EN4.2.2 (Good et al., 2013). We found that the biases in the O_2 trend after 2000 are primarily due to misrepresented O_2^{sat} rather than the non-solubility-driven component. Again, we find that the bias is particularly pronounced after 2000, while it remains relatively small for the period 1967 to 1994. We have included these calculations in Figs. **3**, **S3**, and **S5**. The main text has been changed on lines **151-161** and **284-288** of the revised manuscript.

3. We included an additional observation-based dataset developed by T. Ito (2022), Ito-22, which differs from Ito et al. (2017) in terms of the underlying data and gap-filling methodology. We find that the two Ito products diverge significantly after 2000, highlighting the uncertainties associated with observation-based estimates used to evaluate ocean models. We also show that Ito-22 shows suspiciously strong deoxygenation in the Southern Ocean below 2000 m (Fig. **S6**), where sampling is known to be particularly sparse. We have included the new Ito-22 data in Figs. **2, 3, 4, S3, S5, S6,** and **S12**. We have modified the main text on lines **132-142, 155-166, 284-294**, and **440** of

the revised manuscript.

4. We extended the analysis of regional patterns of change. We have computed spatial maps of O_2 variance in the model and in Ito-17, and find good agreement between the two data classes (Fig. **S8**). We now also show the temporal evolution of O_2 (as well as its solubility and non-solubility-driven components in Fig. **S13**) in four key regions for O_2 dynamics (Fig. **2**): the North Atlantic, the North Pacific, the Equatorial Pacific, and the Southern Ocean. We find good agreement between the model and observation-based estimates, particularly in the North Atlantic and Equatorial Pacific. We show that the regional O_2 variability is more influenced by non-solubility-driven changes than by solubility-driven changes (Fig. **S13**), implying that the biased O_2^{sat} after 2000 should not strongly influence the regional variability. The additional analysis also highlights the importance of the Southern Ocean in setting trends on a global scale. We have changed the main text on lines **186-275** (Section: Regional estimates), **288-289**, and **311-319** of the revised manuscript.

Comment 3.1

Title: "Competing effects of wind and buoyancy forcing on recent ocean oxygen trends". Word "Recent" suggest you are investigating the recent years (i.e. after 2015 or so when the observations show a dramatic decrease). Maybe change the title: "Competing effects of wind and buoyancy forcing on ocean oxygen trends".

Response:

Whilst we agree that 'recent' may be misleading, we still feel that it is useful to provide some temporal categorisation. We have therefore changed the title to: Competing effects of wind and buoyancy forcing on ocean oxygen trends in recent decades.

Comment 3.2

Line 88: Section "Trends in the global ocean oxygen inventory". Authors describe the change of inventory following 4 periods (based on observed trends). Figure 1 and 2 suggests that the HM underestimates the inventory change on average. It is also shown that the HM model is not able to reproduce decadal variations, e.g. the peak around 1985 or the rapid change after 2002. Around 1985 this is probably linked to large change in the SO (Roach and Bindoff 2023); do you see that signal in the model? After 2002, the HM is clearly stagnant (as noted by authors, lines 104-105). Given these differences how can we trust the driver analysis derived from the model (the 3 simulations described in section 2.2.1)?

Response:

Our rationale for the robustness of the driver analysis, despite the bias in the global deoxygenation

trend since 2000, is detailed in our response to the reviewers' general comments above.

Regarding the peak around 1985 mentioned by Roach and Bindoff (2023) and found in the Ito-22 dataset (Fig. **2e**), we would like to point out two aspects:

1. The model may indeed have difficulties in accurately simulating the decadal variability in the Southern Ocean. It is possible that the mismatch in interannual variability in the early period is due to the fact that the precipitation dataset does not include interannual variability prior to 1979 (Tsujino et al., 2018), and even after that, precipitation is less well constrained than other atmospheric observations. Water mass transformation in the Southern Ocean is known to be driven more by freshwater fluxes than heat fluxes (Karstensen and Lorbacher, 2011), so poor constraint on freshwater fluxes is more important in the Southern Ocean than, for example, in the North Atlantic. This discussion is included on lines **247-252**.

2. Globally-regridded observation-based data sets are known to be very imprecise in reproducing the decadal variability of air-sea CO_2 fluxes (Gloege et al., 2021) and oxygen (Ito et al., 2024). This uncertainty is related to the sparseness of the observational sampling (which is particularly critical in the Southern Ocean) and to the different statistical methods used to fill the gaps in space and time. An example of the uncertainty associated with observation-based datasets is shown in our paper: while Ito-22 does show the 1985 peak in oxygen, the Ito-17 dataset shows much more year-to-year variability and no clear peak around 1985. This discussion has been included on lines **256-265** and **288-294**.

Comment 3.3

Line 134: "The acceleration of deoxygenation over the last two decades (Fig. 1) has been caused simultaneously by a smaller increase in wind stress and associated wind-driven O_2 increases, and an increase in O_2 losses driven by changing air-sea heat and freshwater fluxes (Fig. 1a)." I don't clearly understand this point. The model shows the acceleration for the full water column, not in the 0-1000m layer (figure 1b). Those processes impact only the deep ocean ? Please clarify.

Response:

Indeed, the model attributes the acceleration of deoxygenation to changes below 1,000 m. Also, comparing Figs. **S3** and **S5**, one can see that there is a bulge of negative anomalies below 1,000 m between 2002 and 2015, which is not present between 1967 and 1994. This aspect has been included on lines **103-110**.

Comment 3.4

Line 150: "The equatorial regions show an overall deoxygenation that is strongest in the 100-400 m range, consistent with the observed expansion of the tropical OMZs". Note that trends presented in figure S7 for the Indian ocean also show an increase of O_2 in equatorial region; that seems coherent with those simulated in the future, i.e. an increase of O_2 in the tropics around 100-1000m (Ditkovsky et al,2023).

Response:

We thank the reviewer for pointing out this paper, which helps interpret the trends in the tropical areas. This aspect has been included on lines **236-239**.

Comment 3.5

Line 158: "During the strong El Nino conditions of 1997-98 [47], global oxygen levels fell, as simulated by the model and shown by observations (Fig. 1)." Is the ENSO signal from the observations and model really significant ? Why this is not observed for other ENSO events, in 1982-1983 or 2015 ?

Response:

We thank the reviewer for raising this important issue. We agree that it needs to be discussed more thoroughly.

The strong drop in O_2 seen in 1998 is not as marked during the 1982-1983 and 2015-2016 El Niño events. In the model, the 1994-2002 oxygen low is dominated by non-solubility-driven changes due to wind stress (Fig. **1**). Analysing the 1994-1998 wind stress anomalies against the long-term mean (1967-2018), we find that the global oxygen decrease coincides with reduced wind stress in the North Atlantic and equatorial Pacific Ocean. Conversely, during the 1998-2002 global oxygen increase, wind stress in these regions increased relative to the long-term mean, or at least relative to the 1994-1998 period.

Indeed, these wind stress anomaly patterns are reflected in the oxygen time series in the respective sub-regions, with both the equatorial Pacific and (to a lesser total extent) the North Atlantic showing a dip in oxygen levels over the corresponding period 1994-2002.

Yet, the oxygen low in 1998 in the equatorial Pacific is not shown in the observational data (see Fig. **2**, but note that Ito-22 is a 5-year running mean). We have modified the main text accordingly (lines **240-246**).

Comment 3.6

Line 186: "Indeed, we find that the insufficient deoxygenation (<700 m) in the model analysed here (ORCA025-MOPS) is also simulated by global ocean biogeochemistry models (GOBMs) participating in RECCAP2 [56] (Fig. 4a), which largely contribute to the Global Ocean Carbon Budget". Reference 56: I think De Vries et al did not discuss the O_2 budget. It might be relevant to plot the carbon flux from RECCAP2 (like the O_2 and OHC shown in Figure 4). At global scale there is an increase of the carbon sink after 2000 from the data-products (e.g. De Vries et al 2023), a signal not clearly simulated by GOBMs (see also Terhaar et al, 2024). This occurred about the same period (around year 2000) where you identified a shift between ESM and GOBMs (your Figure 4). Would that be linked to the forcing fields used in GOBM as discussed in your conclusion ?

Response:

We believe that the difference between observation-based and simulated carbon uptake and the difference between observation-based and simulated ocean deoxygenation are related. Huguenin et al. (2022) have shown that the spin-up procedure of GOBMs in RECCAP2 and the Global Carbon Budget leads to a too warm ocean with too little oxygen. As a consequence, the ocean warming is too small and solubility-driven deoxygenation is too weak. In addition, a too warm ocean is also likely too stratified already, causing too weak ventilation reductions and hence too little deoxygenation.

The effect of the spin-up procedure on the carbon uptake is similar but not the same. Too weak ventilation would result into too little additional anthropogenic carbon uptake. At the same time, the too warm ocean would lead to too little carbon in the ocean and to a higher uptake capacity (lower Revelle factor). Furthermore, too weak heat uptake leads to too little loss of natural carbon. As the three effects of the spin-up on the carbon sign have opposite sign (and not the same sign as in the case of deoxygenation), the effect of the spin-up on carbon uptake is smaller, leading to a smaller disagreement between simulated and observation-based carbon uptake than the disagreement between simulated and observation-based deoxygenation. However, the existing literature does not yet allow to assess with certainty the effect of the spin-up on the carbon uptake. At the same time, Hauck et al. (2023) have recently shown that not only the simulated carbon uptake is imperfect but also the unequal spacing in space and time lead to a bias towards too high carbon uptake in the observation-based ocean carbon uptake estimates.

While there are other parts that play a role for the simulated carbon uptake (see Terhaar et al., 2024), we believe that the spin-up procedure is the most important one for the ocean deoxygenation as both effects of the the spin-up (too stratified ocean and too little warming) affect the ocean deoxygenation with the same sign (and not with opposite sign as for the carbon uptake).

Comment 3.7

Line 209: "By contrast, coupled Earth system models (ESMs) from CMIP6, which often have the same ocean components as those used in GOBMs, simulate upper 700 m deoxygenation and ocean warming trends that are consistent with observation-based estimates within uncertainties, but at the lower end (Fig. 4)". Given this result, would it be better to use ESM outputs for the driver analysis?

Response:

Because they lack the bias introduced by an inconsistent spin-up procedure, ESMs do indeed provide a better simulation of global oxygen trends. However, there are a number of reasons why a high-resolution global ocean biogeochemistry model, such as the one used here, is preferred for the current study: (1) ESMs do not capture the timing of internal climate variability, whereas hindcast simulations allow to analyse the impact of recent climate variability on ocean deoxygenation, as these simulations are forced with historical atmospheric reanalysis data. (2) Fully coupled ESMs do not allow to isolate the effects of wind and buoyancy forcing, as the atmosphere always evolves as a whole. (3) ESMs often have too coarse a resolution to explicitly simulate mesoscale eddies. High-resolution models have often shown improved realism in ocean circulation and ventilation, which are critical for accurate representation of regional oxygen variability.

Therefore, there seems to be no perfect solution at the moment. Here, we decided to analyse the historical variability in a high-resolution ocean model spun-up using a standard procedure (Tsujino et al., 2020), while thoroughly discussing the causes and implications of the biases. In the future, we recommend that the protocol for the spin-up procedure for ocean models should take into account the recipe proposed by Huguenin et al., 2022 (lines **306-307**).

Advantages and disadvantages of this choice are discussed in lines **70-82** and **311-319**.

Comment 3.8

Line 232: "The underestimation of the strong decline in oxygen and rise in OHC over the last 18 years by GOBMs raises the question of whether the GOBM-derived trends in the ocean carbon sink, as reported by the Global Carbon Budget [57], are similarly biased by systematic biases due to the atmospheric forcing or the hindcast approach in general." After 2000 GOBMs show that the CO_2 sink is low compared to the data-products (see comment C-06). This was identified in previous GCB reports as well (Friedlingstein et al, 2020, 2022). If this is due to atmospheric forcing, then I guess the forcing is not suitable to separate the drivers as discussed in your analysis.

Response:

As described in the response to the reviewers' general comments and in comment 3.6, we find that it is the spin-up strategy, rather than the atmospheric forcing, that produces the large deoxygenation bias.

Comment 3.9

Line 239: "In our simulations, a key region for global oxygen trends is the Southern Ocean..". This is also the region where long term observations are sparse to evaluate the trend accurately.

Response:

We agree with the reviewer that the Southern Ocean (SO) is a region where oxygen observations are sparse, making it difficult to assess the simulated oxygen changes accurately.

We now discuss this issue more thoroughly by pointing out two aspects:

1. The model may indeed have difficulties in accurately simulating the decadal variability in the SO. It is possible that the mismatch in interannual variability in the early period is due to the fact that the precipitation dataset does not include interannual variability prior to 1979 (Tsujino et al., 2018), and even after that, precipitation is less well constrained than other atmospheric observations. Water mass transformation in the Southern Ocean is known to be driven more by freshwater fluxes than heat fluxes (Karstensen and Lorbacher, 2011), so poor constraint on freshwater fluxes is more important in the SO than, for example, in the North Atlantic. This discussion is included on lines **247-252**.

2. Globally-regridded observation-based data sets are known to be very imprecise in reproducing the decadal variability of air-sea CO_2 fluxes (Gloege et al., 2021) and oxygen (Ito et al., 2024). This uncertainty is related to the sparseness of the observational sampling (which is particularly critical in the SO) and to the different statistical methods used to fill the gaps in space and time. An example of the uncertainty associated with observation-based datasets is shown in our paper: while Ito-22 does show the 1985 peak in oxygen, the Ito-17 dataset shows much more year-to-year variability and no clear peak around 1985. This discussion has been included on lines **256-265** and **288-294**.

Comment 3.10

Line 241: "In this region, the recent strengthening of westerly winds, caused by increasing greenhouse gas emissions and stratospheric ozone depletion [31], has counteracted the deoxygenation caused by widespread surface warming and mid-depth freshening [64, 65]." ESM that include both ozone depletion and GHG forcing suggest different response of the carbon fluxes (e.g. Lenton et al 2009). It might be relevant later to test this for O_2. Concerning the freshening, I think there are still large uncertainties in the E/P forcing. Did you test other forcing (e.g. ERA54).

Response:

We agree that the large uncertainties in the E/P forcing hinder an accurate simulation of the Southern Ocean circulation and its variability, especially before 1979 (see previous response).

In a previous study (Patara et al., 2021), we compared the time evolution of the Southern Ocean ventilation using two different atmospheric forcing data sets, specifically the JRA55-do (Tsujino et

al., 2018), used in this study, and the COREv.2 (Large and Yeager, 2009). We find that the two data sets differ significantly in the westerly wind trend, and that this has an impact on the Southern Ocean ventilation trend. We have not tested ERA54.

We thank the reviewer for pointing out the study by Lenton et al. (2009), which we now refer to in lines **322-324**.

Comment 3.11

Line 256: "Our study indicates that the stabilisation or reversal of the past intensification of wind stress, together with continued ocean warming, is likely to accelerate oxygen loss in the future, particularly in the Southern Hemisphere intermediate waters". As the HM is not really reproducing the recent acceleration of O_2 trends (figure 1b), can you really conclude that your results suggest the O_2 decrease will be accelerate in the future due to change in wind stress?

Response:

While the model does not show an acceleration of deoxygenation in the upper 1,000 m with respect to the previous decades, we do see an acceleration over the entire water column.

We now provide a more detailed assessment of the causes and consequences of the deoxygenation bias since the 2000s. Based on our new analysis (explained in detail in the response to the reviewers' general comments), we argue that the HEAT-FW experiment is likely to be more biased than the WIND experiment, which is not designed to capture the direct effects of global warming (but only its indirect effects manifested through changes in wind stress). For these reasons, we still believe that our results point to an accelerated decline in O_2 in the future due to the predicted reduction in wind stress. However, we have toned down the statement and changed *"indicates"* to *"raises the question of whether"* (line **338**).

Comment 3.12

Line 262: "Given the centrality of atmospheric drivers in determining trends in oceanic oxygen, we stress the importance of the accurate representation of wind stress and air-sea heat and freshwater fluxes in reanalysis data for assessing past deoxygenation with GOBMs, which do not capture trends in O_2 and OHC since 2002." I agree with this suggestion; that seems the main conclusion of the analysis, i.e. the HM model is not able, at present, to reproduce observed O_2 changes.

Response:

In the revised manuscript, we attribute the underestimated deoxygenation to the procedure by which GOBMs are typically spun-up, rendering them less sensitive to the global warming that has occurred

since the 1990s. The spin-up protocol we used for this series of experiments is a widely accepted one (Tsujino et al., 2020) and we believe that this study, building on the work of Huguenin et al. (2022) and Takano et al. (2023), contributes to raising awareness in the scientific community of the urgent need to adapt the spin-up protocol accordingly.

We agree with the reviewer that the causes and implications of this bias need to be carefully considered and an assessment made of which results are more or less robust. Our analysis has shown that the largest uncertainty is associated with the trends since 2000, and this uncertainty is mostly associated with the O_2^{sat} component of the O_2 changes. However, the three observation-based data sets considered here also diverge strongly over this period, making it difficult to quantify the model underestimation. We also attribute most of the trend bias to processes occurring in the Southern Ocean, where both model and observation-based estimates of decadal variability are known to be highly uncertain.

While acknowledging these biases, we also highlight the aspects of the simulated oxygen changes that are well simulated. These include: (1) the pre-2002 trends, which are less affected by the recent OHC increase, (2) the trends in the WIND experiment, which by design does not capture the direct effects of global warming (but only its indirect effects manifested through changes in wind fields), (3) the regional patterns of year-to-year variability (which are little affected by the O_2^{sat} bias), and in particular the decadal variability in the North Atlantic, which relies heavily on an accurate representation of the ocean circulation in response to the year-to-year variability in air-sea heat fluxes. In particular, for regional oxygen variability, the use of a relatively high-resolution model, such as the one used here, is clearly an advantage over coarse-resolution ESMs.

For these reasons, we maintain our statement: *"This analysis identifies the drivers of deoxygenation and their regional patterns. It contributes to a much needed improved mechanistic understanding of O_2 changes [...]"* (lines **348-349**).

Comment 3.13

Line 288: What is Cnat ? (natural carbon, i.e. not anthropogenic)

Response:

Yes, this has now been corrected to pre-industrial carbon (line **368**), i.e. not affected by the increase in anthropogenic carbon in the atmosphere.

Comment 3.14

Line 355: "Additionally, in Supplementary Text 1 and Supplementary Figs. 11-13, we compare observational climatological distributions of dissolved oxygen and Osat2 against the corresponding model outputs." I appreciate the comparison of the model with observations. This shows large differences up to -50 or +50 mmol/m3 at regional scale. For example, in the Southern Ocean, differences reach +25 mmol/m3 at depth (1000m, figure S11f). Does that would import more O_2 in the top layers in the model than in reality (i.e. a positive trend of O_2 over time)? Also, here authors compared the climatology from 1958-2018 but as there is a decrease in O_2, would it be better to compare the model for a shorter period (1980-2000 or 1980-1994) and when WOA data are available (few data before 1970).

Response:

In Supplementary Text S2 we give some speculations on the possible origins of the bias: *"The positive bias in deep waters, which are dominated by North Atlantic Deep Water (NADW), may result from overestimated mixed layer depths at subpolar latitudes of the North Atlantic (with respect to ARGO observations) increasing the injection of O_2 into the deep ocean. [...] The positive O_2 anomaly in NADW may also propagate into the Southern Ocean, where it might contribute to the overestimated O_2 concentrations in the deep water masses and, once upwelled at the subpolar divergence, to the overestimates in the subpolar upwelling regions (Fig. S12)"* and now add: *"This may limit the simulated deoxygenation of the Southern Ocean in the model"*.

We agree with the reviewer that it is difficult to interpret mean O_2 values in the presence of drifts. Therefore, we now use a shorter time period, the 1971-2000 climate normal, as provided by the World Ocean Atlas 2023 (for oxygen the shortest period available in the WOA23) for comparison. This period is more suitable for comparison as it excludes the acceleration of deoxygenation when model and observations start to diverge.

Comment 3.15

Figure 1 and others: not easy to distinguish the color of lines (also figures S3 and S6). Maybe use dashed for observations ?

Response:

To make it easier to distinguish between the different lines, we now use more contrasting colours and thicker lines.

Comment 3.16

Figure 1: could you add error range for the Observations (like in figure 2a for Ito-17)?

Response:

The uncertainty estimate we provide for the model data is the range between the minimum and maximum estimates of the two sets of experiments. Therefore, no corresponding uncertainty can be presented for the observational data sets. A note on the observational data sets: For Ito-17, no estimate of uncertainty is provided (see Ito et al., 2017). In the case of GOBAI-O_2, an uncertainty estimate is provided. This uncertainty consists of three separate sources: measurement, gridding and algorithm (see Sharp et al., 2023 for details). Ito-22 includes uncertainty estimates from mapping errors, unresolved small-scale and high-frequency variability (see T. Ito, 2022 for details). We believe that the presentation of these uncertainties in main text Fig. **2** (Fig. **1** in the original submission) would be misleading, as the uncertainty would not be comparable to the uncertainty of our model estimates. We now include the information on the uncertainties in the obs.-based data in the figure caption of Fig. **2**.

In Fig. **3** (Fig. **2** in the original submission), the shading in the line plots indicates the standard error of the estimated slopes and could therefore be calculated equally for model and observation-based data.

Comment 3.17

Figure 2a nicely shows the trend from Ito-17. Could you add also Ito-17 in figures S1a and S2a ?

Response:

As suggested, we have updated and included observation-based estimates in Supplementary Figs. **S3** and **S5** (Figs. **S1** and **S2** in the original submission). We now use Ito-22 as the observational dataset instead of Ito-17, as Ito-22 covers the entire water column, allowing us to compare our model with observations for the entire depth range (see also main text Fig. **3**). We also now provide observational estimates for O_2^{sat}. Unfortunately, neither Ito-17 nor Ito-22 provide an estimate for O_2^{sat}. Instead, we have used the observation-based ocean reanalysis EN4 (version 4.2.2, https://www.metoffice.gov.uk/hadobs/en4/), which provides temperature and salinity datasets, to calculate O_2^{sat}.

Comment 3.18

Figure S7: Is it useful to show the trend over 1967-2018 as the trends are different depending the periods. Maybe show the same but for 2 periods.

Response:

In Fig. **S6** (Supplementary Fig. **7** in the original submission), we now show trends between 1967 and 2015, and additionally show observed trends using the observation-based data product Ito-22 (temporal coverage: 1965-2015). We do not show trends between 1958 and 1967, as we decided not to include this initial period in our analyses due to the initial shock and associated recovery phase that the model undergoes when the forcing jumps from 2018 back to 1958 (see Methods).

Comment 3.19

Figure S7: is it possible to compose the same figure S7 with observations (suggestion)?

Response:

We updated Fig. **S6** (Supplementary Fig. **7** in the original submission) to additionally show observations.

Comment 3.20

Figure S7: why for the Indian Ocean the results are presented north of 30°N ?

Response:

Indeed, the areas were mistakenly filled with zeros, appearing as off-white in our colormap. We have corrected this error and updated Fig. **S6** (Supplementary Fig. **7** in the original submission) accordingly.

Comment 3.21

It would be interesting to include maps of O_2 trend in surface (0-100m) and at 500m and compare with observations. Same could be prepared for O_2 inventory trend (in 0-1000m, see Ito et al, 2024, their figure 3).

Response:

As suggested, we now show maps of observed and modelled O_2 inventory trends for the depth ranges 0-300 and 0-1000 metres (Fig. **S12**).

Gloege, L., G. A. McKinley, P. Landschützer, A. R. Fay, T. L. Frölicher, J. C. Fyfe, and others (2021), Quantifying errors in observationally based estimates of ocean carbon sink variability, *Global Biogeochem. Cycles*, **35**, e2020GB006788, doi:10.1029/2020GB006788

Good, S. A., M. J. Martin, and N. A. Rayner (2013), EN4: Quality controlled ocean temperature and salinity profiles and monthly objective analyses with uncertainty estimates, *J. Geophys. Res. Oceans*, **118**, 6704–6716, doi:10.1002/2013JC009067

Hauck, J., C. Nissen, P. Landschützer, C. Rödenbeck, S. Bushinsky, and A. Olsen (2023), Sparse observations induce large biases in estimates of the global ocean CO_2 sink: an ocean model subsampling experiment, *Phil. Trans. R. Soc.*, A.38120220063, doi:10.1098/rsta.2022.0063

Huguenin, M. F., R. M. Holmes, and M. H. England (2022), Drivers and distribution of global ocean heat uptake over the last half century, *Nat. Commun.*, **13**, 4921, doi:10.1038/s41467-022-32540-5

Ito, T., S. Minobe, M. C. Long, and C. Deutsch (2017), Upper ocean O_2 trends: 1958–2015, *Geophys. Res. Lett.*, **44**, 4214–4223, doi:10.1002/2017GL073613

Ito, T. (2022), Optimal interpolation of global dissolved oxygen: 1965–2015, *Geoscience Data Journal*, **9**, 167–176, doi:10.1002/gdj3.130

Ito, T. and H. E. Garcia, Z. Wang, S. Minobe, M. C. Long, J. Cebrian, and others (2024), Underestimation of multi-decadal global O_2 loss due to an optimal interpolation method, *Biogeosciences*, **21**, 747-759, doi:10.5194/bg-21-747-2024

Karstensen, J. and K. Lorbacher (2011), A practical indicator for surface ocean heat and freshwater buoyancy fluxes and its application to the NCEP reanalysis data, *Tellus A*, **63**, 338-347, doi:10.1111/j.1600-0870.2011.00510.x

Large, W. G., S. G. Yeager (2009), The global climatology of an interannually varying air–sea flux data set, *Clim. Dyn.*, **33**, 341–364, doi:10.1007/s00382-008-0441-3

Lenton, A., F. Codron, L. Bopp, N. Metzl, P. Cadule, A. Tagliabue, and J. Le Sommer (2009), Stratospheric ozone depletion reduces ocean carbon uptake and enhances ocean acidification, *Geophys. Res. Lett.*, **36**, L12606, doi:10.1029/2009GL038227

Patara, L., C. W. Böning, and T. Tanhua (2021), Multidecadal changes in Southern Ocean ventilation since the 1960s driven by wind and buoyancy forcing, *J. Climate*, **34**, 1485–1502, doi:10.1175/JCLI-

D-19-0947.1

Polo, I., J. Robson, R. Sutton, and M. A. Balmaseda (2014), The Importance of Wind and Buoyancy Forcing for the Boundary Density Variations and the Geostrophic Component of the AMOC at 26°N, *J. Phys. Oceanogr.*, **44**, 2387–2408, doi:10.1175/JPO-D-13-0264.1

Roach, C. J., and N. L. Bindoff (2023), Developing a new oxygen atlas of the world's oceans using data interpolating variational analysis, *J. Atmos. Oceanic Technol.*, **40**, 1475–1491, doi:10.1175/JTECH-D-23-0007.1

Sharp, J. D., A. J. Fassbender, B. R. Carter, G. C. Johnson, C. Schultz, and J. P. Dunne (2023), GOBAI-O_2: temporally and spatially resolved fields of ocean interior dissolved oxygen over nearly 2 decades, *Earth Syst. Sci. Data*, **15**, 4481–4518, doi:10.5194/essd-15-4481-2023

Takano, Y., T. Ilyina, J. Tjiputra, Y. A. Eddebbar, S. Berthet, L. Bopp, E. Buitenhuis, and others (2023), Simulations of ocean deoxygenation in the historical era: insights from forced and coupled models, *Front. Mar. Sci.*, **10**, 1139917, doi:10.3389/fmars.2023.1139917

Terhaar, J., N. Goris, J. D. Müller, T. DeVries, N. Gruber, J. Hauck, and others (2024), Assessment of global ocean biogeochemistry models for ocean carbon sink estimates in RECCAP2 and recommendations for future studies, *J. Adv. Model. Earth Sy.*, **16**, e2023MS003840, doi:10.1029/2023MS003840

Tsujino, H., S. Urakawa, H. Nakano, R. J. Small, W. M. Kim, S. G. Yeager, and others (2018), JRA-55 based surface dataset for driving ocean–sea-ice models (JRA55-do), *Ocean Modelling*, **130**, 79–139, doi:10.1016/j.ocemod.2018.07.002

Tsujino, H., S. Urakawa, S. M. Griffies, G. Danabasoglu, A. J. Adcroft, A. E. Amaral, T. Arsouze, and others (2020), Evaluation of global ocean-sea-ice model simulations based on the experimental protocols of the Ocean Model Intercomparison Project phase 2 (OMIP-2), *Geoscientific Model Development*, **13**, 3643-3708, doi:10.5194/gmd-13-3643-2020

4 Figure Changes

Table 1: Summary of the new figures and the changes in the revised figures with respect to the first submission. Minor changes that only affect the appearance (e.g. changes to the colour scheme) are not explicitly listed.

**Fig. No.**	**Previous**[a]	**Changes**
Fig. 1	unchanged	(i) we shifted the comparison with the observation-based estimates to Fig. 2, (ii) we removed the percentage contributions of solubility and non-solubility-driven components to the overall trends, (iii) we removed years 1958-1967
Fig. 2	Fig. 1	(i) we added the obs.-based Ito-22 data set, (ii) we added oxygen inventory time series for four sub-regions
Fig. 3	Fig. 2	(i) we substituted Ito-17 with the Ito-22 data set, which extends below 1,000 m depth, (ii) we added observation-based O_2^{sat} estimates based on the EN4.2.2 data set, (iii) we use the combination of both new obs.-based data sets to estimate the non-solubility-driven component of oxygen changes in observations
Fig. 4	unchanged	we added the obs.-based Ito-22 data set
Fig. 5	Fig. 3	R^2 is now calculated for 1967-2018 instead of 1958-1967
Fig. S1	Fig. S3	now includes linear fit for globally integrated remineralisation rate in the hindcast experiment
Fig. S2	new figure	—
Fig. S3	Fig. S2	(i) we added the observation-based Ito-22 data set (ii) we added observation-based O_2^{sat} estimates based on the EN4.2.2 data set (iii) we use the combination of both new obs.-based data sets to estimate the non-solubility-driven component of oxygen changes in observations
Fig. S4	new figure	—
Fig. S5	Fig. S1	same as Fig. S3
Fig. S6	Fig. S7	(i) we added the observation-based Ito-22 data set (and thus show trends over 1967-2015 instead of 1967-2018), (ii) we added contour lines showing neutral density surfaces surfaces
Fig. S7	Fig. S4	unchanged
Fig. S8	new figure	—
Fig. S9	new figure	—
Fig. S10	Fig. S6	added observations of AMOC stream function from RAPID
Fig. S11	Fig. S5	unchanged
Fig. S12-14	new figure	—
Fig. S15	Fig. S10	unchanged
Fig. S16	Fig. S11	we now use a shorter time period, the 1971-2000 climate normal (using the WOA23 instead of the WOA18), for comparison
Fig. S17	Fig. S12	same as Fig. S16
Fig. S18	Fig. S13	same as Fig. S16

[a] figure number at first submission

Dear Reviewers,

We would like to thank all of you for your thoughtful and constructive feedback on our manuscript. We appreciate the time and effort you have put into reviewing our work and are pleased that the revisions have addressed your comments. We are grateful for your recognition of these efforts and your support for publication. Your detailed reviews and recommendations have been instrumental in bringing this manuscript to its final form.

Yours sincerely and on behalf of all co-authors,
Helene Hollitzer

1 Response to reviewer 3

General Comments

I am very satisfied with author's responses and the revised manuscript, including revised figures in the main text and in the Supplementary Material. Their responses to all reviewers are very well prepared.

For comparing the model with observations, authors now used the Ito-22 data-set and thus enabled to compare the full water column. In addition they mentioned significant differences between the 3 data-sets, an interesting result that opens questions regarding the origin of these differences, a challenge for new analysis dedicated for a better evaluation of O2 changes in the ocean and thus to validate model results GBOM or ESM.

In the revision, authors described and interpret in more detail the anomalies at regional scale (e.g., North Atlantic and convection, Southern Ocean and wind stress strengthening) that would also help to interpret the changes in other properties (e.g. anthropogenic CO2 inventories).

The manuscript offers several message for the modeling community (adapt the spin-up protocol, test different forcing fields) and for the observational community (more observations especially in the Southern Ocean, add deep Argo-O2 floats in the future).

The paper is suitable for publication in its present form. See very few comments below that could be taken into account for the final version.

Response:

We thank the reviewer for their positive evaluation and the additional constructive and helpful comments. We have taken each comment into account, provide responses to each point below, and adapted the manuscript accordingly.

Comment 3.1

Line 188-193: Authors write: "The highest levels of O2 short-term variability are found, for example, in strongly dynamic regions of water mass formation, frontal dynamics, and ocean-sea ice interaction (Supplementary Fig. 8). Instead, there is little variability in the centre of the subtropical gyres and in the Weddell and Ross gyres, and within the OMZs due to their inherently low oxygen concentrations". Not sure to see that in Figure S8. The model does not show high variability in ocean-ice region but this apparently captured from observations. Also, the observations and the model both show high variability in the eastern boundaries (OMZ sector?).

Response:

We have rephrased "frontal dynamics" to "strong currents" to include also the Eastern Boundary Currents as areas of high short-term variability, and removed the OMZs as areas of low short-term variability, as suggested by the reviewer.

However, we have not changed the part about the sea ice regions. In the observations, zones of high short-term variability in the sea-ice zone are larger due to the small number of observations, especially in the Southern Ocean. Thus, high variability in a small zone is extrapolated to large areas, especially below the sea ice where there are almost no observations. In the high-resolution model, however, the variability of each cell is simulated and the variability is actually located where the inter-annual variability of the sea ice is high. These locations can be seen as a small band around the Southern Ocean and as small spots in the Nordic Seas. In the revised manuscript we now write:

"The spatial extent of regions of high O_2 variability in observation-based products often appears to be larger than modelled, especially in the Southern Ocean. This relatively large spatial extent results from the scarcity of observations that have to be extrapolated over large spatial scales each year. As a consequence, the O_2 values in each cell of the observational product are not always derived from observations at the same location, and the high variability regions of the observation-based product are stretched. The high-resolution model, on the other hand, correctly captures only the inter-annual variability at specific locations and does not need to rely on spatial extrapolation."

Comment 3.2

Line 204-206: Authors write: "By comparing these regional trends with observation-based estimates, we find that while global deoxygenation is generally underestimated, regional trends can be captured with greater accuracy, e.g. in the North Atlantic and equatorial Pacific." You may also notice that in the North Atlantic there is a very good comparison with GOBAI-O2 after 2010 as shown in Figure 2c (but this is somehow written on lines 223-224).

Response:

As suggested by the reviewer, we have changed the sentence to: "By comparing these regional trends with observation-based estimates, we find that while global deoxygenation is generally underestimated,

regional trends can be captured with greater accuracy (Fig. 2). In the North Atlantic, for example, the O2 estimates from Ito-17 and GOBAI-O2 are very similar to the O2 time series simulated by the model after 1990 and 2010, respectively (Fig. 2c)."

Comment 3.3

Line 259: Authors write: "Large disagreements exist also between the trends estimated by observation-based datasets in the Southern Ocean (Fig. 2e)." This is also an interesting result in this manuscript; any idea why the datasets present so large differences ? Curiosity: Is the result would be the same when changing the limit of the Southern Ocean taken at 40°S, 45°S or 50°S (here authors used 30°S).

Response:

We do not have a definite answer, but we have included some hypotheses in the manuscript:

"Large disagreements exist also between the trends estimated by observation-based datasets in the Southern Ocean (Fig. 2e). The differences between Ito-22 and Ito-17 can be attributed to three main factors [42]: data sources, interpolation methods, and mapping parameters. Ito-22 relies solely on WOD bottle O_2 data, while Ito-17 combines both bottle and CTD data, resulting in different spatial coverage and data density. The restricted availability of source data, particularly in Ito-22, results in substantial data gaps that must be interpolated [42], introducing significant uncertainty, especially in the under-sampled Southern Ocean. In comparison, GOBAI-O_2 uses a broader data set, including both GLODAP bottle O_2 data and Argo float measurements, and employs machine learning techniques, including random forest regression and feed-forward neural networks, for data set development [43]. The large discrepancies in the Southern Ocean are therefore likely to reflect the sensitivity of these observation-based datasets to different data sources, and the challenges of interpolation in poorly sampled regions such as the Southern Ocean."

We also recalculated the trends using different boundaries for the Southern Ocean, but found no significant changes in the results (see Figure below). As expected, with a more southerly boundary, the differences between datasets decrease due to the reduced volume being integrated. However, the relative differences remain consistent across these boundary adjustments.

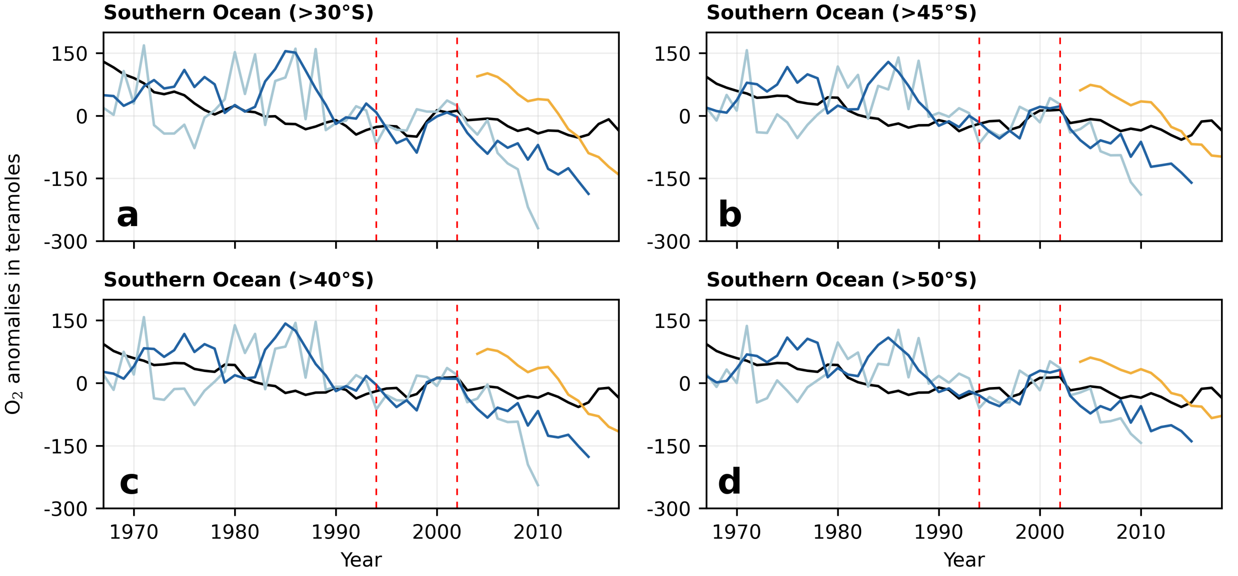

Figure 1: **Change in Southern Ocean oxygen inventory in the hindcast experiment and comparable observation-based data.** Time series (1967-2018) of (a) Southern Ocean oxygen inventory anomalies simulated by ORCA025-MOPS HIND (black) and observation-based data from Ito-17 (Ito et al., 2017, light blue), Ito-22 (T. Ito, 2022, dark blue) and GOBAI-O_2 (Sharp et al., 2023, yellow). Subpanels (b-d) show the same as (a), but with different latitudinal boundaries of the Southern Ocean. For each dataset, the data are mean centred using the long-term mean calculated over the time span plotted.

Comment 3.4

C-04: Line 264: Authors write: "As observation-based estimates of the ocean carbon sink have also been shown to overestimate the variability in the Southern Ocean [68] as well as the trends [69], we cannot conclude with certainty if models or observation-based estimates are closer to reality. ." What do you mean by "reality"? Surface trends or inventories ? I agree with authors, but I guess this sentence somehow mixed different topics. This depends on the property and "reality" one is looking for. For example the view of the changes (and "real changes") of air-sea CO2 fluxes and Cant inventories in the Southern Ocean also depends on the boundary selected (see comment C-03) and selected period.

Response:

The word reality referred to the time series of O_2 in the real world, which models and observational estimates seek to quantify. As the reviewer pointed out, the sentence mixed up different issues, carbon and oxygen, and we have clarified this in the revised manuscript:

"The accuracy of observation-based estimates of interior O_2 changes in the Southern Ocean is likely to be low, given that even observation-based estimates of the air-sea CO_2 flux, which are based on a larger number of surface ocean observations than estimates of interior O_2 change, have also been shown to overestimate variability in the Southern Ocean [68] as well as trends [69]. Due to the shortcomings of model and observation-based estimates of O_2 inventory changes in the Southern Ocean, we cannot conclude with certainty whether model or observation-based estimates are closer to the true O_2 inventory changes in this ocean basin."

Comment 3.5

C-05: Figure S6: I guess iso-sigma plotted are the same for each basin. Maybe just indicate this in the caption (no need to revise the figure).

Response:

The iso-sigma values shown were global averages. We have adjusted the figure to show the neutral density surfaces for each individual basin (simulated by the hindcast HIND). We agree with the reviewer that this should be indicated in the caption for clarity and have therefore adjusted the caption accordingly.
